# Efficacy and safety of bispecific antibodies vs. immune checkpoint blockade combination therapy in cancer: a real-world comparison

**DOI:** 10.1186/s12943-024-01956-6

**Published:** 2024-04-16

**Authors:** Linyan Cheng, Lujun Chen, Yuan Shi, Weiying Gu, Weidong Ding, Xiao Zheng, Yan Liu, Jingting Jiang, Zhuojun Zheng

**Affiliations:** 1 Department of Hematology, The Third Affiliated Hospital of Soochow University, Changzhou, Jiangsu Province China; 2 Department of Tumor Biological Treatment, the Third Affiliated Hospital of Soochow University, Changzhou, Jiangsu Province China; 3 Jiangsu Engineering Research Center for Tumor Immunotherapy, Changzhou, China; 4 Institute for Cell Therapy of Soochow University, Changzhou, China; 5 Laboratory of Hematology, The Third Affiliated Hospital of Soochow University, Changzhou, China; 6 Department of Hematology, Beijing Hospital, National Center of Gerontology, Institute of Geriatric Medicine, Chinese Academy of Medical Sciences, Beijing, China; 7 Graduate School of Peking Union Medical College, Chinese Academy of Medical Sciences, Beijing, China

**Keywords:** Bispecific antibodies, Immune checkpoint inhibitors, Cancer, Efficacy, Safety

## Abstract

Emerging tumor immunotherapy methods encompass bispecific antibodies (BSABs), immune checkpoint inhibitors (ICIs), and adoptive cell immunotherapy. BSABs belong to the antibody family that can specifically recognize two different antigens or epitopes on the same antigen. These antibodies demonstrate superior clinical efficacy than monoclonal antibodies, indicating their role as a promising tumor immunotherapy option. Immune checkpoints are also important in tumor immunotherapy. Programmed cell death protein-1 (PD-1) is a widely acknowledged immune checkpoint target with effective anti-tumor activity. PD-1 inhibitors have demonstrated notable therapeutic efficacy in treating hematological and solid tumors; however, more than 50% of patients undergoing this treatment exhibit a poor response. However, ICI-based combination therapies (ICI combination therapies) have been demonstrated to synergistically increase anti-tumor effects and immune response rates. In this review, we compare the clinical efficacy and side effects of BSABs and ICI combination therapies in real-world tumor immunotherapy, aiming to provide evidence-based approaches for clinical research and personalized tumor diagnosis and treatment.

**Supplementary Information:**

The online version contains supplementary material available at 10.1186/s12943-024-01956-6.

## Introduction

Cancer is one of the most serious diseases, posing a major threat to human life and health. A 2022 cancer statistics report in the United States reveals that nearly 1,700 people continue to succumb to various cancers daily, even in an era of remarkably advanced treatment [1]. Among cancer therapies, immunotherapies that activate immune components—immune checkpoint inhibitors (ICIs), therapeutic antibodies, cancer vaccines, and immune cell therapy, are gaining prominence. Immunotherapy has largely revolutionized cancer treatment in the last few years, with patients with cancer who receive immunotherapy often exhibit superior tolerability and have shown significant improvements in long-term survival [2], underscoring its pivotal role for oncology treatment.

In recent years, bispecific antibodies (BSABs) have emerged as a novel strategy in tumor immunotherapy. BSABs combine two distinct antigen targets within a single antibody molecule, potentially enhancing clinical efficacy and safety. This dual targeting approach has accelerated the development and widespread adoption of BSABs [3]. The following are main types of BSABs are currently available. (a) Effector cell engagers: One end of the BSAB recruits T or natural killer (NK) cells via specific receptors, while the other end recognizes the tumor-associated antigen, leading to the redirection of effector cells to tumor tissue to kill the tumor cells. Furthermore, the T-cell activation-induced release of cytokines facilitates the recruitment of other immune cells, thereby enhances the immune response to the tumor [4]. (b) Tumor-targeted immunomodulators: These BSABs simultaneously target tumor antigens and immunomodulatory receptors to activate the immune response in the tumor microenvironment (TME), causing enhanced selective killing by effector cells and reduced side effects of systemic immune activation. (c) Dual immunomodulators: In these BSABs, different immunomodulatory targets are combined to achieve overlapping or synergistic antitumor effects. (d) Dual tumor-targeted antibodies: The mechanism of these BSABs involves inhibiting tumor proliferation, metastasis, and angiogenesis by targeting different oncogenic signaling pathways [2, 5]. In summary, BSABs eliminate tumor cells via the following primary mechanisms. (1) BSABs recruit and activate immune cells to infiltrate tumor tissues, thereby amplifying their tumor-killing efficacy. (2) BSAB molecules block the various signaling pathways of tumor development, promote tumor cell apoptosis, inhibit tumor proliferation and metastasis, and suppress tumor angiogenesis. (3) Lastly, BSABs target different cell surface antigens or epitopes in the tumor or its microenvironment, block immune escape signals of the tumor, and enhance the specific binding between cells and tumor, leading to direct tumor cell death [5, 6].

Immune checkpoints are surface receptor proteins on immune cells that regulate the activation or inhibition of the immune response. ICIs enhance antitumor effects by amplifying the immune cell activation at different stages of the immune cycle [7]. Classical ICIs, such as programmed cell death protein-1 (PD-1), PD-L1, cytotoxic T-lymphocyte-associated antigen 4 (CTLA-4), and lymphocyte activation gene-3 (LAG-3) inhibitors, have been approved for the treatment of various cancers. These ICIs have improved long-term survival and quality of life for certain patients. However, many patients exhibit limited response and poor clinical efficacy following ICI therapy. Such limitation has motivated researchers to explore the therapeutic strategy of combining various ICIs to achieve synergistic antitumor effects by targeting different immunoregulatory pathways in the TME [8]. Both preclinical models and clinical studies have shown that combination therapy often outperforms monotherapy in terms of immune response and survival rates. However, ICI combination therapies carry a higher risk of adverse reactions, posing serious challenges in clinical practice [9].

PD-1 is a prevalent receptor on the surface of tumor-infiltrated T, B, and NK cells. Within the TME, the effector and exhausted T cells exhibit high PD-1 expression, whereas its ligand PD-L1 is commonly found on various tumor surfaces [10]. The binding of PD-1 with PD-L1 dampens immune responses by curtailing cell proliferation, cytokine secretion, and the cytotoxicity of effector immune cells [11], thus playing a crucial role in immune surveillance evasion. CTLA-4 (CD152) is predominantly expressed on the surface of activated T cells as well as in regulatory T cells (Tregs) and PD-1^+^ CD4^+^/CD8^+^ tumor-infiltrating lymphocytes (TILs). The binding of CTLA-4 to the T-cell’s B7 receptor (CD80/86) reduces interleukin-2 (IL-2) production, impedes T-cell proliferation, and triggers cell cycle arrest [12]. Moreover, PD-1 and CTLA-4 expression levels are higher in TILs than in normal tissues and peripheral blood mononuclear cells [9]. Correspondingly, the simultaneous inhibition of the CTLA-4 and PD-1 signaling pathways has demonstrated synergistic activity in colon cancer and melanoma animal transplantation models [13, 14]. This blockade of the CTLA-4 and PD-1 signaling pathways can synergistically enhance the anti-tumor immune responses in patients, thereby improving their immune response rates. Treg-infiltrated tumor tissues preferentially and consistently express LAG-3. Its co-expression with other immune checkpoint molecules (PD-1, TIGIT, and TIM3) results in T-cell exhaustion, a typical tumor immune escape mechanism [15]. LAG-3 and PD-1 have been detected to be co-expressed on CD4^+^ and CD8^+^ TILs in preclinical mouse tumor models, suggesting that their co-blockade of the LAG-3 and PD-1 signaling pathways can enhance the proliferation of tumor-specific CD8^+^ T cells and cytokine release [16]. Additionally, in patients with ovarian cancer, LAG-3 and PD-1 co-expression is associated with the dysfunction or depletion of CD8^+^ T cells [17]. These findings suggest that a combined anti-LAG-3 and anti-PD-1 strategy could thus be a pivotal tumor immunotherapy approach, more effectively reversing T-cell exhaustion. TIGIT, a T-cell immunosuppressive receptor, is predominantly expressed on T and NK cell surfaces [18] and has shown pronounced upregulation in tumors, including non-small cell lung cancer (NSCLC) and colon cancer. Such elevated expression is associated with advanced disease status and poor prognosis [19]. TIGIT contributes to tumor immune evasion through various immunity mechanisms, including inhibiting NK cell-mediated cytotoxicity, suppressing T-cell proliferation, restricting CD8^+^ T cell activation in the TME, and promoting inflammatory CD4^+^ T cell responses to impede tumor apoptosis [20]. The combined inhibition of the TIGIT and PD-1/PD-L1 signaling pathways can also lead to the synergistic enhancement of the proliferation and function of anti-tumor CD8^+^ T cells, thus boosting anti-tumor efficacy and ultimately improving overall patient survival. In congruence with this notion, numerous preclinical models have shown that administering anti-TIGIT antibodies alongside anti-PD-1 or PD-L1 inhibitors results in nearly complete tumor remission, whereas treatment with anti-TIGIT antibodies alone elicits limited efficacy [19].

**Fig. 1:**
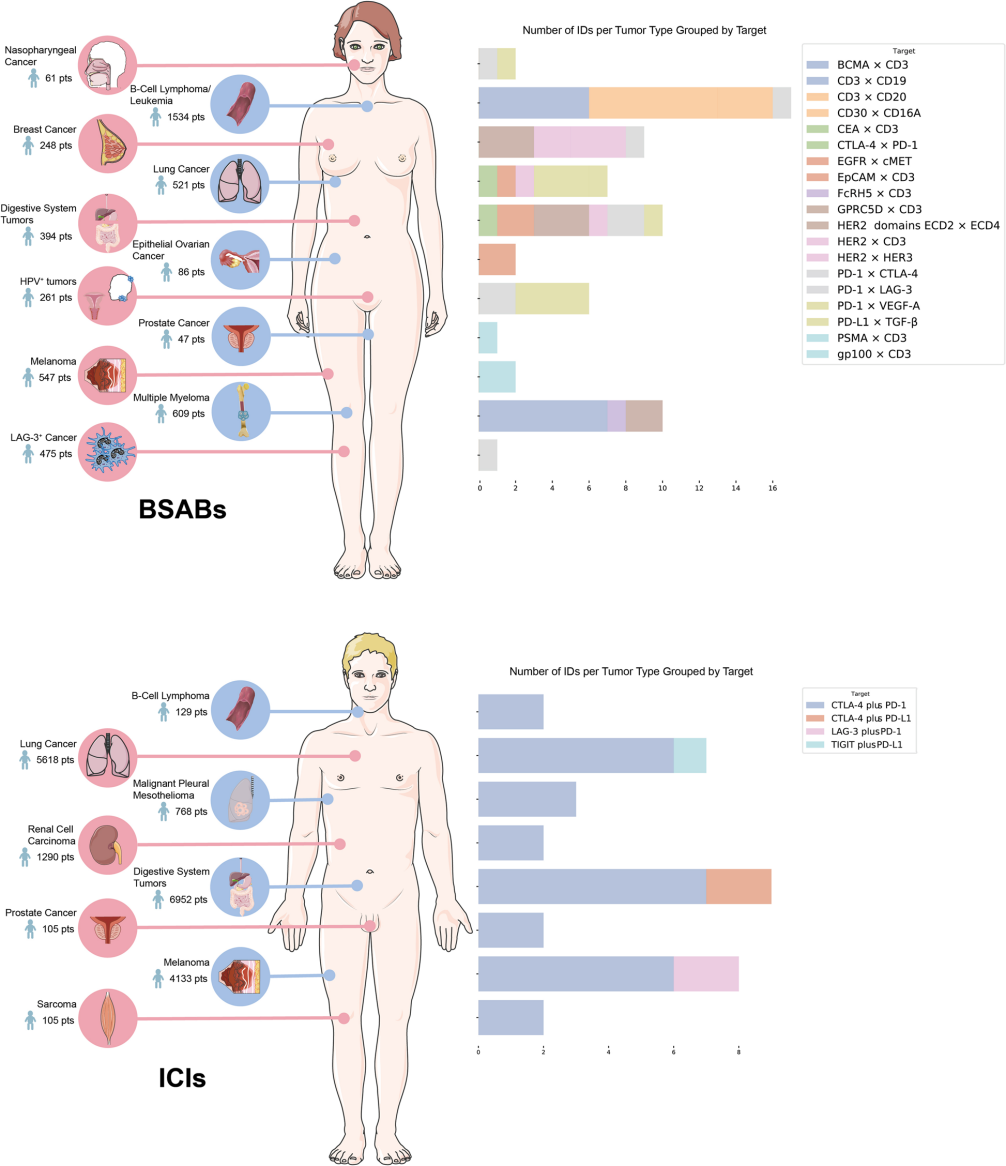
Schematic diagram of the clinical application of bispecific antibodies and ICI combination.** a **Landscape of clinical trials of BSABs in human cancers. LAG-3, lymphocyte activation gene-3. **b **Landscape of Clinical trials of ICI combination therapies in human cancers

Numerous research centers worldwide have extensively investigated the combined use of BSABs and ICI therapies in treating hematological malignancies and solid tumors. In this review, we aim to summarize and compare the efficacy and adverse reactions of these immunotherapies across various tumor types (This review includes data from 23, 883 patients, comprising 4, 783 in clinical trials of BSAB and 19, 100 in ICIs) (Fig. 1a, b). Furthermore, we aspire to lay the groundwork for clinical treatment approaches and proposed novel personalized precision therapeutic strategies for the patients with various tumor types.

## B-cell lymphoma/leukemia

Human B-cell lymphoma/leukemia typically originates from the germinal center or post-germinal center B cells and is characterized by frequent chromosomal ectopic events in the immunoglobulin (Ig) gene loci, alongside robust cell proliferation [21]. The prognosis is particularly poor in adult patients with relapsed or refractory (R/R) precursor B-cell acute lymphoblastic leukemia (B-ALL). Salvage treatments in such cases have been shown to produce complete remission (CR) in 30–45% of patients, with a median overall survival (mOS) of 5–9 months [22, 23]. Furthermore, allogeneic hematopoietic stem cell transplantation (HSCT) from a matched donor is currently the only treatment option for adult patients with R/R acute lymphoblastic leukemia (ALL). However, achieving CR prior to allogeneic HSCT is crucial. Therefore, novel therapeutic approaches are required to increase the chances of attaining CR, thereby enhancing the likelihood of successful allogeneic HSCT and achieving long-term cure [24]. B-cell non-Hodgkin’s lymphoma (NHL) is a mature B-cell malignancy, with diffuse large B-cell lymphoma (DLBCL) and follicular lymphoma (FL) being the most common subtypes. Although DLBCL is a curable disease, many patients, especially those with early relapsed or primary refractory disease, rarely achieve prolonged progression-free survival (PFS). This outcome is particularly significant for patients who have received CD20 monoclonal antibodies (Mabs) due to the lack of available effective salvage options [25]. FL is considered an incurable disease, can nevertheless achieve long-term remission through the administration of anti-CD20 Mabs or standard alkylating agents. However, approximately 10–20% of patients with early relapsed or primary refractory FL experience poor prognostic outcomes or early mortality [26]. Lastly, in the case of Hodgkin’s lymphoma (HL), first-line therapy may enable patients to obtain long-term remission or cure. However, 10–30% of them may experience disease progression or relapse, with second-line therapy yielding a cure rate of < 50% [27].

### Bispecific antibodies

CD19 is a crucial antigen target with high expression in most B-cell lymphomas/leukemias, including NHL, ALL, chronic lymphocytic leukemia, and hairy cell leukemia [28]. Blinatumomab, an anti-CD3/CD19 BSAB, is a T-cell engaging therapy that redirects cytotoxic T cells to tumor cells exhibiting high CD19 expression by targeting CD3. These redirected cytotoxic T cells then release perforin/granzyme B to eliminate the tumor cells [29]. Blinatumomab was initially developed for Philadelphia chromosome-negative patients with R/R B-ALL. A phase II trial by Topp et al. demonstrated that blinatumomab treatment (9 µg/day for 7 days, followed by 28 µg/day; over 2–4 weeks every 6 weeks, for up to five cycles) achieved CR in 33% of the patients and an mOS of 6.1 months after two cycles, along with a median PFS (mPFS) of 6.9 months after two cycles in patients with CR. However, blinatumomab treatment also caused side effects, including grade ≥ 3 adverse events (AE) in 82% of the patients, dose-dependent neurotoxicity (such as encephalopathy, confusional state, somnolence, and cognitive disorder) in 13%, and cytokine release syndrome (CRS) in 2% [30]. A subsequent phase III trial comparing blinatumomab treatment with chemotherapy revealed that blinatumomab administration led to a significantly longer mOS than chemotherapy (7.7 months vs. 4.0 months), as well as significantly a better CR rate and mPFS and fewer side effects after transplantation than the chemotherapy arm. Additionally, while blinatumomab treatment resulted in a lower incidence of myelosuppression, it caused more serious AEs than chemotherapy, particularly in terms of neurological events (9.4% vs. 8.3%) and CRS incidence (4.9% vs. 0%) [31]. Blinatumomab received approval for the treatment of relapsed refractory B-ALL in 2014.

Blinatumomab has also been employed in treating NHL with high CD19 expression, exhibiting promising therapeutic efficacy. Goebeler et al. recruited 76 patients with NHL for a phase I trial, including individuals with DLBCL (*n* = 14), FL (*n* = 28), mantle cell lymphoma (*n* = 24), and other NHLs (*n* = 10). The trial results revealed that the maximum tolerated dose (MTD, 60 µg/m^2^) of blinatumomab achieved an overall response rate (ORR) of 69% among all NHL subgroups and an ORR of 55% in the DLBCL subgroup, with 20% of the patients experienced severe neurological events, primarily comprising encephalopathy, aphasia, and headache [32]. In light of Blinatumomab’s remarkable response rate in DLBCL, a sequential phase II trial of blinatumomab for treating R/R DLBCL was conducted by enrolling patients into either a dose escalation or flat-dosing regimen. However, the cohort of patients receiving a flat dosage was closed due to severe adverse reactions. Subsequently, an ORR of 42% (CR, 19%) was demonstrated among all evaluable patients, with four patients being discontinued from the study due to serious neurological toxicity events (encephalopathy, somnolence, epilepsy, or aphasia). Nevertheless, appropriate blinatumomab dosing for DLBCL should be continued to be explored to reduce the occurrence of early treatment interruption due to drug-related adverse reactions [33]. Coyle et al. further explored blinatumomab as a secondary salvage treatment for managing patients with aggressive B-cell NHL (B-NHL), with 83% of patients having DLBCL. The study showed favorable efficacy rates, with an ORR of 37% and a complete metabolic response (CMR) rate of 22%. Notably, the CMR rate was higher in patients who relapsed after first-line therapy than in those refractory to it (39% vs. 14%), suggesting that the early administration of blinatumomab as a salvage treatment may benefit patients with R/R aggressive B-NHL [34]. Another study evaluated the effectiveness of blinatumomab combined with lenalidomide in treating R/R NHL patients. This regimen involved initial administration of blinatumomab combined with lenalidomide, followed by up to 6 cycles of consolidation therapy and lenalidomide maintenance therapy for 2 years. The patients exhibited an ORR of 83% (CR, 50%), with an mPFS of 8.3 months. Moreover, this treatment regimen showed good safety, with only 5.5% of the patients experiencing grade 3 neurotoxicity and no cases of grade 3/4 CRS or treatment-related deaths [35].

CD20/CD3 BSABs are also used for managing indolent and aggressive NHL. In vitro and in vivo targeting of CD20 via BSABs has been reported to stimulate highly cytotoxic activity against CD20-expressing B cells, including primary leukemia and lymphoma cells. Furthermore, these BSABs can induce B-cell depletion and activation as well as the proliferation of CD4^+^/CD8^+^ T cells and cytokine release [36]. Mosunetuzumab is a full-length humanized IgG1 BSAB against CD20/CD3 designed to target B-cell lymphoma. Budde et al. conducted a phase I dose-escalation trial of mosunetuzumab in heavily pretreated patients with R/R B-NHL. The study found that the dose-escalation strategy reduced CRS occurrence, leading to a CRS incidence of 27.4% (grade ≥ 3 CRS, 1.0%) within the first cycle mainly. Additionally, the ORRs of patients with aggressive and indolent B-NHL were 34.9% (CR, 19.4%) and 66.2% (CR, 48.5%), respectively. Moreover, in patients with aggressive and indolent B-NHL who achieved CR, the median duration of response (mDoR) was 22.8 and 20.4 months, respectively. Lastly, the trial estimated a recommended dose of 1/2/60/60/30 g for phase II trials [37]. Subsequently, an expanded phase II trial was performed in patients with FL, wherein 90 patients received 1 mg (day 1 of cycle 1, D1C1), 2 mg (D8C1), 60 mg (D15C1 and D1C2), and 30 mg (D1C3) over a 3-week cycle. The trial results showed that 60% of the patients achieved CR, while 44% exhibited CRS (grade ≥ 3 CRS, 5%). The most common grade ≥ 3 AEs were neutropenia, hypophosphatemia, hyperglycemia, and anemia [38]. Other CD20/CD3 BSABs have also shown excellent efficacy in alleviating NHL, including in relapsed or CAR-T-resistant NHL (Supplementary Table S1) [39–43]. Another BSAB, epcoritamab, has exhibited significantly higher efficacy than other CD3/CD20 BSABs in patients with DLBCL. Epcoritamab, when administered at doses of ≥ 48 mg, attained an ORR of 91% (CR, 55%) in patients with DLBCL, while patients with FL also experienced significant benefits (ORR, 90%; CR, 50%). Additionally, the CRS observed in this treatment was grade 1 or 2, with neurotoxicity events of grade ≥ 3 occurring in only 3% of patients [41]. A phase I trial of glofitamab combined with obinutuzumab pretreatment in a population with refractory aggressive B-NHL indicated mitigation of CRS occurrence. Glofitamab also exhibited favorable activity (ORR, 53.8%; CR, 36.8%) at low CD20 expression levels, along with durable responses (an mDoR of 5.5 and 10.8 months and an mPFS of 2.9 and 11.8 months in patients with aggressive NHL and FL, respectively) [44].

As mentioned earlier, LAG-3 and PD-1 are inhibitory receptors on immune cells that can synergistically aid tumor evasion [16]. In patients with DLBCL, LAG-3 is highly expressed on CD4^+^ Tregs and CD8^+^ TILs, while its co-expression with PD-1 and TIM-3 has also been observed on certain B-cell lymphomas. Studies have indicated that the high expression of LAG-3/PD-L1 in tumor tissue results in lower survival rates among patients with DLBCL [45]. Tebotelimab (MGD013) is a BSAB that targets PD-1 and LAG-3. In patients with R/R DLBCL, MGD013 treatment yielded an ORR of 50% (CR, 14%; PR, 36%), with fever being the primary adverse reaction. In addition, researchers analyzed samples of patients with relapsed DLBCL after CAR-T therapy and found that the LAG-3 and PD-1 expressions on tumor-infiltrating T cells and B-cell lymphoma increased after CAR-T therapy. An increase in effector T cells and enhanced tumor lysis were also observed following MGD013 administration [46].

Classical HL is primarily characterized by the presence of Reed–Sternberg cells (also known as Hodgkin and Reed–Sternberg [HRS] cells), usually expressing CD15 and CD30. Early studies employing CD30 Mabs to ameliorate HL did not reveal effective anti-tumor activity, possibly due to the inhibition of antibody-dependent cellular cytotoxicity (ADCC) and antibody-dependent cellular phagocytosis in heavily pretreated and immunosuppressed patients with HL [47]. Brentuximab vedotin (BV) is an antibody-drug conjugate that targets CD30. A phase II trial using BV for patients with R/R HL reported an ORR of 75% (CR, 35%), particularly in those who had progressed after autologous stem cell transplantation (ASCT) [48]. However, most patients treated with BV have been found to experience tumor progression. AFM13 is a BSAB against CD30/CD16A that induces cytotoxicity of NK cells and stimulates macrophages to promote innate immune responses. In the setting of R/R HL, AFM13 has demonstrated good tolerability and safety (dose ≥ 1.5 mg/kg; ORR, 23%), including in BV-refractory patients. The main side effects associated with this regimen were injection-related adverse reactions (68%), usually resolved by standard therapeutic measures [27]. A phase II study involving 25 patients with R/R HL treated with BV and PD-1 inhibitors reported similar clinical outcomes (ORR, 16.6%; CR, 4.2%) [49]. A study by Green et al. observed that the amplification of chromosome 9p24.1 represents a reproducible genetic abnormality in nodular sclerosis classical HL. This 9p24.1 amplification leads to the overexpression of PD-L1, PD-L2, and JAK2 genes, enabling HRS cells to escape the immune response via the PD-1/PD-L1 axis [50]. Thus, blocking the PD-1/PD-L1 axis may be a useful immunotherapy strategy for patients with HL. Considering their high response rates (69% each), nivolumab and pembrolizumab have been approved for managing R/R HL after ASCT and BV failure [51, 52]. Additionally, a phase Ib trial showed that a combination of AFM13 and pembrolizumab in patients who were relapsed or refractory to BV resulted in an early high response rate (ORR, 83%; CR, 37%) without additional toxicity [53].

### ICI combination therapies

HRS cells are extensively surrounded by inflammatory and immune cells, with CTLA-4 being the most abundantly expressed immune checkpoint receptor in the TME of HL. Furthermore, CTLA-4^+^ T cells often congregate around HRS cells, promoting the immune evasion capability of HRS cells [54]. The CheckMate-039 trial evaluated the efficacy of a regimen of nivolumab (3 mg/kg) in combination with ipilimumab (1 mg/kg) in a cohort that had not previously undergone anti-PD-1 or ASCT therapy. Although the trial yielded a high response rate (ORR, 74%; CR, 19%), it was not significantly higher than that of PD-1 inhibitors [55]. In another phase I trial, the combined treatment of ICI combination therapies and BV administration demonstrated higher response rates and better long-term remission (ORR, 82%; CR, 73%; 2-year OS rate, > 80%) than either monotherapy in early follow-up (including in patients who previously underwent ASCT). Although this study found evidence of higher immunotherapeutic toxicity, the researchers suggested that nivolumab combined with BV or triple therapy may improve HL prognosis. Therefore, long-term follow-up of these therapies is still required, along with the exploration of optimal therapeutic biomarkers [56].

**Fig. 2:**
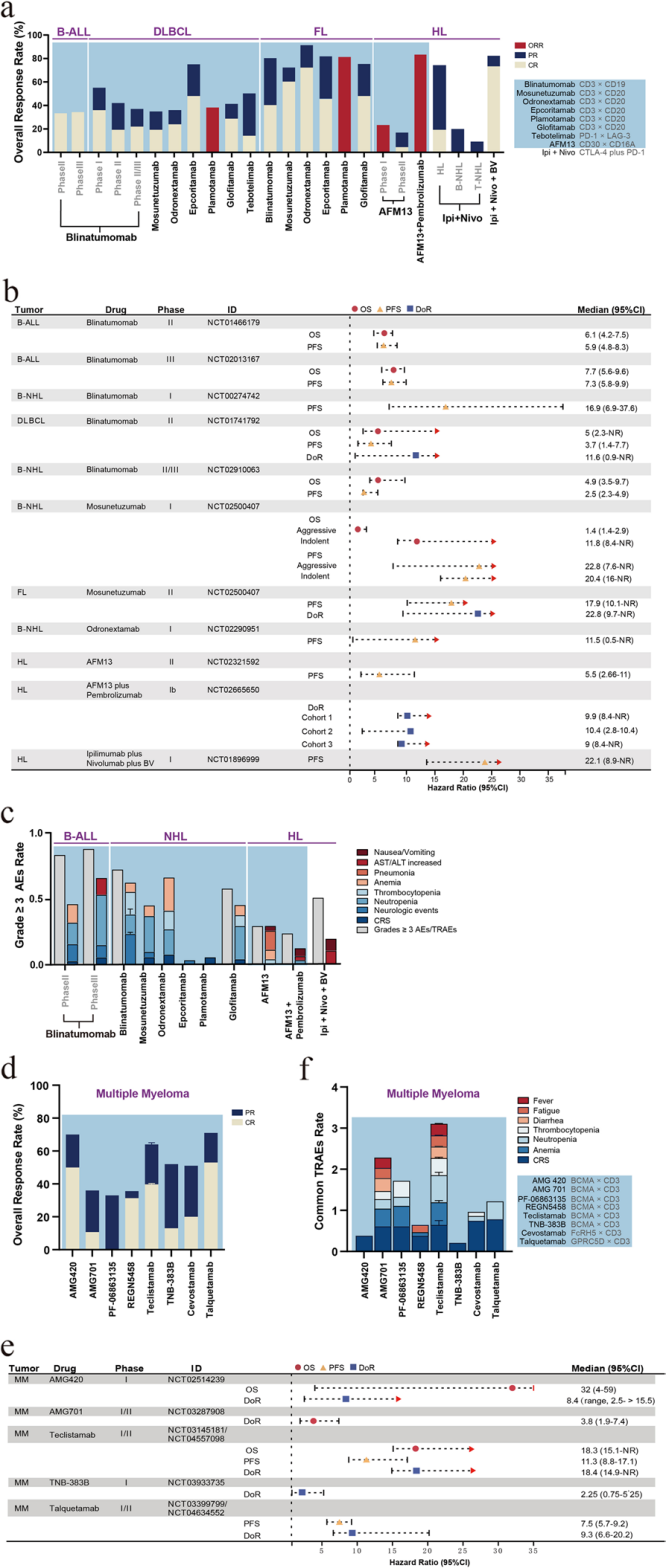
Efficacy, adverse events, and survival outcome of bispecific antibody treatment and ICI combination therapies in patients with hematological malignancies. **a** A histogram depicting the antitumor efficacy of BSABs and ICIs combination therapies as applied to patients with B-cell lymphoma/leukemia. The ORR, defined as a sum of CR and PR. ORR, overall response rate; CR, complete remission; PR, partial response; Ipi, Ipilimumab (anti-CTLA-4); Nivo, Nivolumab (anti-PD-1); BV, Brentuximab vedotin. **b** A forest plot charting the survival outcomes (unit is month) of BSABs and ICIs combination therapies in patients with B-cell lymphoma/leukemia. OS, overall survival; PFS, progression-free survival; DoR, duration of response; NR, not reached. **c** A histogram depicting the incidence of grade ≥ 3 adverse events (AEs), as well as the major compositions of grade ≥ 3 AEs or treatment-related adverse events (TRAEs) with BSABs and ICIs combination therapies in patients with B-cell lymphoma/leukemia. A bar with a value of 0 means not mentioned in the article. **d** A histogram depicting the antitumor efficacy of BSABs and ICIs combination therapies as applied to patients with multiple myeloma. **e** A forest plot charting the survival outcomes of BSABs and ICIs combination therapies in patients with multiple myeloma. **f** A histogram depicting the incidence of the composition of major grade ≥ 3 AEs for the treatment of BSABs and ICIs combination therapies for multiple myeloma, with cytokine release syndrome (CRS, all-grade), while other adverse events were graded as tertiary or higher

In summary, BSABs were first applied in hematologic malignancies, showing significant clinical efficacy and achieving CR in most patients. In patients with R/R NHL, CD19/CD3 and CD20/CD30 BSABs demonstrated better treatment efficacy in indolent FL than in DLBCL (ORR, 66–91% vs. 35–75%) [32, 37–39, 41, 43, 44]. The early clinical trial results of BSABs against PD-1/LAG-3 also offer new therapeutic prospects for managing patients with R/R DLBCL, albeit with a slightly lower CR (9%) [46]. In the case of HL, improved response rates were associated with the treatment strategies of CD30/CD16A BSABs combined with pembrolizumab (ORR, 83%), ICI combination therapies (ORR, 74%), or ICI combination therapies accompanied with BV administration (ORR, 82%) in patients with R/R HL (Fig. 2a and Supplementary Table S1) [27, 49, 53, 56]. Additionally, BSABs targeting CD20/CD3 were also able to attain good therapeutic efficacy in heavily pretreated patients with NHL (ORR, 33%), including those who had relapsed or exhibited resistance to CD20 Mabs or CAR-T therapy. Furthermore, compared to CD19/CD3 BSABs, CD20/CD3 BSABs may provide greater survival benefits, particularly in terms of PFS. Moreover, the survival data of patients with HL treated with ICI combination therapies and CD30/CD16A BSABs revealed that ICI combination therapies might facilitate longer PFS (Fig. 2b and Supplementary Table S1). In the case of treatment-related complications, CRS and neurotoxicity constitute the notable adverse reactions observed after treatment using CD19/CD3 and CD20/CD30 BSABs for B-ALL and NHL. Nevertheless, improving the antibody structure and adjusting the dose should decrease the incidence of these AEs. In addition, the incidence of AEs caused by BSAB administration in patients with HL is lower than that caused by ICI combination therapies, with BSAB treatment leading to AEs such as pneumonia, elevated liver enzyme levels, and nausea/vomiting, while ICI combination therapies were linked with AEs including high liver enzyme levels and nausea/vomiting (Fig. 2c and Supplementary Table S1).

## Multiple myeloma

The hallmark of multiple myeloma (MM) is the proliferation of malignant plasma cells (PCs) within the bone marrow (BM), resulting in the excessive production of monoclonal Igs in the patient’s blood and urine, as well as the development of clinically evident osteolytic bone lesions [57]. The survival outcome of MM has improved owing to the emergence of immunomodulatory drugs (IMiDs) and proteasome inhibitors (PI). However, MM eventually develops resistance to these two drug classes, with a study demonstrating an mOS of 11.2 months in patients refractory to IMiDs or PI alone and an mOS of 5.6 months in those refractory to both medications (penta-refractory patients). Therefore, MM recurrence is an inevitable outcome, indicating that the current foremost therapeutic strategy for MM involves exploring novel targets to prolong the survival of patients with MM [58].

### Bispecific antibodies

B-cell maturation antigen (BCMA) is preferentially expressed in mature B lymphocytes. Low expression levels of BCMA on plasmacytoid dendritic cells have been shown to promote the survival of malignant PCs within the BM microenvironment. Additionally, overexpression of BCMA in MM enhances tumor proliferation, activates osteoclasts, and promotes angiogenesis, metastasis, and immunosuppression-related gene expression. Further, serum BCMA can form complexes that inhibit B cell-activating factor of the TNF family (BAFF) activity, leading to immunodeficiency in patients with MM [59]. AMG420 (BI 836,909), a BSAB targeting BCMA/CD3, has demonstrated efficient and selective killing of BCMA^+^ MM cells in in vitro and in vivo experiments. Moreover, its activity remains unaffected by BM stromal cells and serum BCMA [60]. A study by Topp et al. investigated the effects of AMG420 administration in patients with R/R MM, including those refractory to IMiD and PI treatment. The study results showed that AMG420 treatment (400 µg/day) achieved an ORR of 70%, with 38% of the patients experiencing CRS (one with grade 3 CRS). Moreover, AMG420 administration attained a better mPFS (23.5 months) than BCMA-CAR-T therapy for MM (mPFS < 12 months), indicating comparatively significant and durable biological activity. Additionally, the safety profile was manageable, mainly including symptoms such as anemia, diarrhea, fatigue, and fever [61, 62]. Other BSABs against BCMA have also demonstrated favorable ORRs (36–65%; Supplementary Table S2) in treating patients with R/R MM, including those refractory to three types of drugs [63–68].

Fc receptor-homolog 5 (FcRH5), a type I transmembrane protein containing Ig domains, is exclusively expressed in the B-cell lineage and is retained in PC expression profiles. Compared to normal B cells, PCs and MM cells exhibit elevated expressions of FcRH5. Cevostamab (BFCR4350A) is a BSAB against FcRH5/CD3 that binds to the proximal membrane domain of FcRH5 on MM cells, leading to their targeted killing [69]. Preliminary data suggests that patients who respond to cevostamab treatment demonstrate pronounced T-cell expansion in the peripheral blood and an increased proportion of CD8^+^ tumor-infiltrating T cells [70]. In a trial investigating the preliminary clinical activity and safety of cevostamab, cevostamab monotherapy showed potential efficacy in a large cohort of pretreated patients with R/R MM. In particular, cevostamab administration was linked with substantial and sustained responses in patients with high-risk cytogenetics as well as in those with triple-class refractory MM. Cevostamab regimen at an initial/target dose of ≥ 3.6/20 mg achieved an ORR of 51.7%, with a CRS incidence of 74.5% [71]. Furthermore, the addition of 8 mg/kg of tocilizumab was found to reduce CRS incidence (35.7%) without any significant negative effect on the anti-tumor activity [72].

G protein-coupled receptor family C group 5 member D (GPRC5D) exhibits high selective expression on MM cell surfaces, and it remains unaffected by various anti-tumor therapies, such as IMiDs, PI, and CD38 Mabs [73]. Talquetamab (a GPRC5D/CD3 BSAB) was investigated in a phase I trial conducted by Krishnan et al. involving 137 patients treated with either intravenous or subcutaneous injection of talquetamab. The confirmed recommended phase II dose (RP2D) was 800 µg/kg/week of subcutaneous talquetamab, attaining an ORR of 71% (≥ very good partial response [VGPR] rate, 53%). Common adverse reactions included anemia, neutropenia, and lymphopenia, with CRS (mostly grade 1 or 2) occurring in 47% of the patients [74].

In summary, BSABs employed in MM are mainly associated with T cells, including BSABs targeting BCMA/CD3, FcRH5/CD3, and GPRC5D/CD3. Among the BCMA/CD3 BSABs, AMG420 led to a high clinical response rate and improved long-term survival; however, it was discontinued due to its requirement for continuous infusion, a procedure that is difficult to perform in clinics. Research on AMG701 has also been halted owing to its high adverse reaction rate. Compared to other BCMA/CD3 BSABs, teclistamab has demonstrated higher efficacy and longer survival (ORR, 63%; CR, 39.4%; mOS, 18.3 months) [67] in triple-refractory patients and has therefore been approved for marketing by the FDA. Although FcRH5/CD3 and GPRC5D/CD3 BSABs also produce higher clinical response rates (ORR, 51.7% and 71%, respectively) than BCMA/CD3 BSABs, the currently available survival data indicates no significant improvement compared to teclistamab (Fig. 2d, e and Supplementary Table S2) [71, 74]. Moreover, despite the higher adverse reactions, including myelosuppression, diarrhea, and fever, associated with teclistamab treatment, the incidence of grade > 3 CRS was only 0.6%. In contrast, the main AEs of FcRH5/CD3 and GPRC5D/CD3 BSAB treatment were CRS, which occurred at a higher frequency than that in BCMA/CD3 BSAB therapy. Considering these findings, follow-up studies are required to further assess the balance between clinical efficacy and therapeutic toxicity of BSABs for MM (Fig. 2f and Supplementary Table S2).

## Lung cancer

Lung cancer is the most common cause of cancer deaths worldwide, accounting for an estimated 1.6 million deaths per year. Approximately 85% of all lung cancers occur as NSCLCs. The targeted therapy in NSCLCs with epidermal growth factor receptor (EGFR) and anaplastic lymphoma kinase mutations has shown significant improvement in the disease prognosis (ORR, 58–83%). However, drug resistance may still develop in certain patients [75]. For example, EGFR exon 20 insertion (ex20ins) mutations hinder the binding of tyrosine kinase inhibitors (TKI) to EGFR, leading to drug resistance [76], with such mutations representing 12% of all EGFR mutations [77]. Although platinum-based chemotherapy is the standard first-line therapy for NSCLC with EGFR ex20ins mutations, it is associated with a poor prognosis, the ORR is 20%, with an mPFS ranges from 4.5 to 5.7 months, and an mOS is 17 months. Furthermore, for patients who progress after platinum-based chemotherapy, the second-line therapy yields an ORR of 14%, with an mOS of 11.5 months and an mPFS of 3.3 months [78]. In the case of patients with NSCLC having no targetable mutations, the first-line therapy is primarily platinum-based combination chemotherapy. However, patients do not exhibit a durable response to chemotherapy (mPFS, 4.3 months; mOS, 13.9 months), with approximately 50% mortality among responsive patients within a year [79]. Small-cell lung cancer (SCLC) comprises an estimated 14% of all lung cancers, with most presenting with widespread metastases and a short survival period. Furthermore, SCLC has been reported to show poor response to second-line therapy, while its response to drugs is also not durable [80].

### Bispecific antibodies

Amivantamab is a BSAB that inhibits EGFR mutations and the cMet signaling pathway. A preclinical study by Moores et al. suggested that amivantamab induces receptor internalization of EGFR and cMET, thereby inhibiting their related downstream signaling pathways and suppressing tumor proliferation. Additionally, amivantamab may enhance the anti-tumor activity of immune cells via increased interferon-γ (IFN-γ) secretion by tightly binding to the FcγRIIIa fragment. Moreover, these anti-tumor mechanisms of amivantamab are more effective than those of TKIs and cetuximab [81]. In an extension cohort study, Park et al. demonstrated that amivantamab had a robust and durable efficacy in patients with NSCLC exhibiting disease progression after platinum-based chemotherapy. The study reported on patients having NSCLC with EGFR ex20ins mutations who were treated with the RP2D of 1050 mg of amivantamab (1400 mg for those weighing ≥ 80 kg; once per week [qw] during the first 4 weeks, followed by once every 2 weeks [q2w] starting from the 5th week). The treatment regimen achieved an ORR of 40%, with an mOS of 22.8 months. AEs associated with the targeted EGFR inhibition included rash (86%), paronychia (45%), and stomatitis (21%), whereas hypoalbuminemia (27%; grade 3: 3%) and peripheral edema (18%) were related to the inhibition of the cMet signaling pathway [82]. Consequently, amivantamab was approved for treating adult patients having locally advanced or metastatic NSCLC with EGFR ex20ins mutations. A real-world analysis of amivantamab therapy in patients with NSCLC presenting with disease progression after platinum-based chemotherapy demonstrated a significantly improved ORR compared to other anticancer treatments (40% vs. 16%), including platinum-based chemotherapy, immunotherapy, and TKI therapy [83]. Transforming growth factor β (TGF-β) upregulates PD-L1 gene transcription via the phosphorylation of Smad2, with PD-L1 expression in NSCLC showing a positive correlation with Smad2. Bintrafusp alfa (M7824), a TGF-β/PD-L1 bifunctional fusion protein, has been found to weaken TGF-β1-mediated epithelial-mesenchymal transition (EMT) and block PD-L1-dependent immunosuppression in NSCLC. Moreover, bintrafusp alfa was revealed to increase NSCLC sensitivity to chemotherapy by inhibiting TGF-β signaling [84]. Paz-Ares et al. also conducted a study investigating the efficacy of bintrafusp alfa treatment in patients with advanced or metastatic NSCLC that had progressed after standard first-line therapy. The research determined that a 1200 mg dosage of the drug elicited an ORR of 25.0% (*n* = 10), with an mOS of 15.6 months. Furthermore, bintrafusp alfa demonstrated significant efficacy, particularly in patients who showed high expression of PD-L1 (≥ 80%), achieving an ORR of 85.7%. However, 69% of the patients experienced treatment-related AEs (TRAEs), of which 17.5% had immune-related AEs (two AEs were grade 4) and 8.8% had TGF-β-mediated skin reactions [85].

KN046 is a novel BSAB that inhibits the interaction of PD-L1/CTLA-4 with CD80/CD86. A phase II trial evaluated the efficacy and safety of KN046 administration in patients with metastatic NSCLC. In this trial, patients received 3 mg/kg or 5 mg/kg of KN046 q2w. The results demonstrated that the dose of 3 mg/kg led to an ORR and disease control rate (DCR) of 10.7% and 82.1%, respectively, while the dose of 5 mg/kg attained an ORR and DCR of 15.6% and 62.5%, respectively. Moreover, the efficacy was more prominent in patients with squamous cell carcinoma (mPFS, 7.3 months; 9-month OS rate, 88.2%) [86].

Lung cancer can lead to the overexpression of vascular endothelial growth factors (VEGFs), resulting in the promotion of tumor metastasis and invasion and EMT, as well as the secretion of different VEGF isoforms. Additionally, VEGF plays a key role in regulating the immune response within the TME by reducing immune infiltration, inducing the proliferation of Tregs and myeloid-derived suppressor cells, and promoting T-cell exhaustion [87]. Anti-angiogenic drugs have been shown to stimulate the immune response by inducing tumor vascular normalization and directly affecting immune cells [88]. Furthermore, immunotherapy combined with anti-VEGF drugs has demonstrated potential as an effective strategy for managing lung cancer. In a mouse model of lung cancer, bevacizumab (a VEGF inhibitor) and cytokine-induced killer (CIK) cells synergistically inhibited tumor growth while promoting CIK cell infiltration [89]. A clinical trial by Zhou et al. investigated the use of AK112 (a VEGF/PD-1 BSAB; dosage, > 10 mg/kg) in patients with advanced NSCLC. Their findings revealed an ORR of 42.9% across all patients and an ORR of 56.3% in those with high PD-L1 expression, indicating encouraging outcomes [90]. Subsequently, Zhao et al. recruited a cohort of patients with advanced NSCLC for a phase II trial, wherein patients received AK112 combined with chemotherapy (10 mg/kg or 20 mg/kg every 3 weeks). The early results indicated a PR of 63% and a DCR of 92.3% among all evaluable cohorts. Moreover, patients with squamous cell carcinoma exhibited more pronounced benefits (ORR, 77.8%; 6-month PFS rate, 83.3%). The combination treatment also achieved an ORR of 68.4% and an mPFS of 8.2 months in patients having advanced non-squamous NSCLC with EGFR mutations who had failed EGFR-TKI therapy. Similarly, an ORR of 40% and an mPFS of 6.6 months were obtained in patients with advanced NSCLC that had progressed after treatment with PD-1 inhibitors combined with platinum-based chemotherapy. In the case of adverse reactions, treatment-emergent AEs occurred in 86.5% of the patients, with 28.6% experiencing grade ≥ 3 AEs (including two deaths). The most common AE included elevated liver enzyme levels and epistaxis. Overall, AK112 treatment demonstrated favorable safety and was not associated with severe bleeding or perforation AEs observed in VEGF target-related AE. Additionally, compared to the combined treatment of PD-1 or PD-L1 inhibitors with chemotherapy +/− anti-angiogenic drugs, AK112 combined with chemotherapy exhibited significant antitumor activity across different patient populations with advanced NSCLC [91].

Neuregulin-1 (NRG1) gene fusions are an emerging oncogenic driver commonly associated with the human epidermal growth factor receptor 3 (HER3), a member of the receptor tyrosine kinase family. This NRG1-HER3 interaction promotes the binding of HER2 with HER3 and participates in the downstream signaling pathways involved in cell proliferation and growth, ultimately leading to tumorigenesis [92]. A previous research study reported NRG1 fusions in lung cancers, particularly invasive mucinous adenocarcinoma [93]. Zenocutuzumab (MCLA-128) is a HER2/HER3-targeting BSAB that functions by docking and blocking the structural domains of these proteins, thereby preventing the binding of NRG1-HER3 ligands and NRG1 fusion proteins and subsequently disrupting the downstream signaling pathways to produce an anti-tumor effect. Zenocutuzumab has also been shown to induce enhanced ADCC activity [94]. A preclinical modeling study indicated that administering zenocutuzumab in patients with NRG1-positive cancer could lead to persistent clinical responses [95]. The eNRGy study by Schram et al. evaluated the efficacy and safety of zenocutuzumab treatment in patients with NRG1^+^ solid tumors. In the NSCLC cohort (41 patients) of this study, an ORR of 35% (34% for NRG1^+^ tumors) was obtained, with < 5% of the patients experiencing ≥ grade 3 adverse reactions. The trial also assessed zenocutuzumab treatment outcomes in other NRG1^+^ tumors, including pancreatic and breast cancer (see their details in the corresponding sections below) [96].

### ICI combination therapies

Studies have demonstrated that nivolumab can also be used in patients with pretreated advanced NSCLC, improving their OS and providing durable survival benefits (mOS, 14.9 months; 1-year OS rate, 56%; 3-year OS rate, 27%) [97]. A multicohort trial in untreated advanced NSCLC reported that the treatment regimen of 3 mg/kg of nivolumab (q2w) in combination with 1 mg/kg of ipilimumab (q6w) elicited manageable tolerability and a sustained response rate [98]. A subsequent phase III trial was conducted by Hellmann et al. utilizing the same regimen as the first-line therapy for patients with advanced NSCLC. The study findings revealed that the ICI combination therapies (nivolumab and ipilimumab) were more effective than chemotherapy, regardless of the PD-L1 expression levels (mOS, 17 vs. 12.2–14.9 months). Furthermore, in patients with a high tumor mutation burden (TMB, ≥ 10 mutations per megabase [mut/Mb]), the ICI combination therapies achieved greater clinical benefits than chemotherapy (ORR, 45.3% vs. 26.9%; mPFS, 7.2 vs. 5.5 months) [99]. Another phase III study by Rizvi et al. also suggested that patients with a blood TMB ≥ 20 mut/Mb who received combined anti-PD-1 and anti-CTLA-4 therapy experienced improved OS compared to those who underwent chemotherapy (21.9 vs. 10 months) [100]. The phase III study by Hellmann et al. also provided long-term follow-up data that indicated that patients who received ICIs combination therapies (nivolumab and ipilimumab) continued to exhibit sustained clinical benefits even after discontinuing their immunotherapy compared to the patients who underwent chemotherapy (5-year OS rate, 24% vs. 14%) [101]. Based on these phase III research results of Hellmann et al., the combined treatment of nivolumab and ipilimumab was approved by the FDA in May 2020 as a first-line treatment for patients with metastatic NSCLC who are negative for driver genes and have a PD-L1 expression level of ≥ 1%. In a phase II trial of patients with advanced PD-L1^+^ NSCLC, tiragolumab (anti-TIGIT) plus atezolizumab (anti-PD-L1) treatment demonstrated significant improvement compared to atezolizumab monotherapy (ORR, 37.3% vs. 20.5%; mPFS, 5.6 vs. 3.9 months). In terms of AEs, the treatment-related toxicity was similar (grade ≥ 3 TRAEs, 14.9% [tiragolumab plus atezolizumab] vs. 19.1% [atezolizumab]), mainly consisting of skin rash and infusion-related adverse reactions [102].

Multiple studies have indicated that chemotherapy can stimulate the immune system, damage tumor suppression mechanisms, and enhance the tumor-killing effectiveness of ICIs [103]. Paz-Ares et al. further investigated the efficacy of combining nivolumab and ipilimumab with two chemotherapy cycles in patients with NSCLC. This phase III trial found that this ICI combination therapy plus chemotherapy provided greater durable survival benefits than chemotherapy alone (mOS, 15.6 vs. 10.9 months; 3-year OS rate, 27% vs. 19%) [104–106]. Additionally, Cascone et al. assessed the efficacy of ICI combination therapies (nivolumab plus ipilimumab) as a neoadjuvant treatment during the perioperative period of patients with operable NSCLC. In this study, patients received nivolumab (3 mg/kg, days 1/15/29) in combination with ipilimumab (1 mg/kg, day 1). The results showed that the ICI combination therapy facilitated the recruitment of an increased number of immune cells to infiltrate the tumor and enhance pathological response (nivolumab + ipilimumab vs. nivolumab; major pathologic response [MPR] rate, 38% vs. 22%; pathologic complete remission [pCR] rate, 29% vs. 9%) [107].

In the SCLC cohort of the Checkmate-032 study, the combination regimen of 1 mg/kg of nivolumab (q2w) with 3 mg/kg of ipilimumab (q3w) for four cycles, followed by 3 mg/kg of nivolumab (q2w), demonstrated early clinical benefits (ORR, 25%; 1-year OS rate, 42%) [108, 109]. Furthermore, patients with a high tumor burden had an extended survival period (combination regimen vs. nivolumab alone; mOS, 10.7 vs. 6.6 months) [110].

**Fig. 3:**
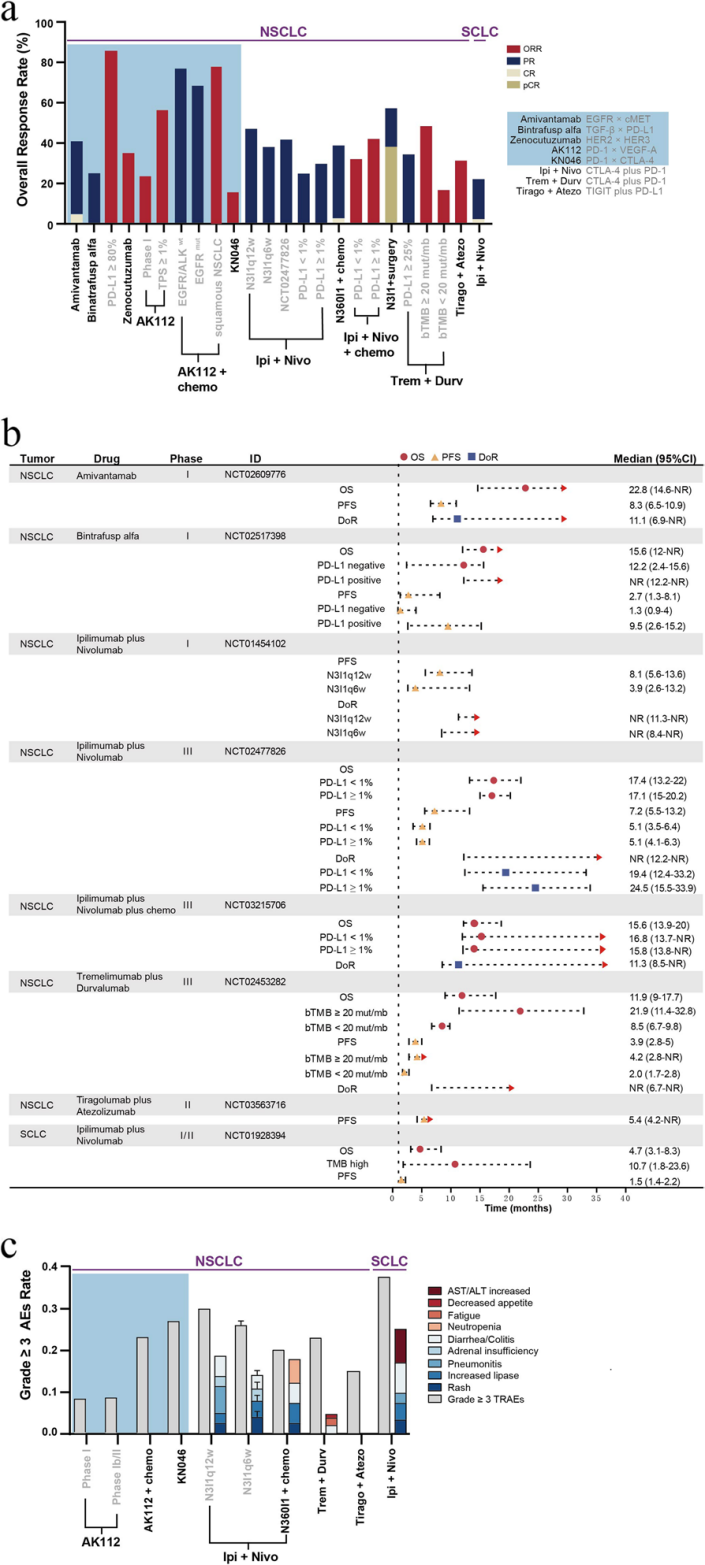
Efficacy, adverse events, and survival outcome of bispecific antibody treatment and ICI combination therapies in patients with Lung cancer. **a** A histogram depicting the antitumor efficacy of BSABs and ICIs combination therapies in patients with lung cancer, including various subgroups. The ORR, defined as a sum of CR and PR. pCR, pathological CR; ALK, anaplastic lymphoma kinase; wt, wild-type; mut, mutation; N3I1q12w, 3 mg/kg Nivo q2w plus 1 mg/kg Ipi q12w; N3I1q6w, 3 mg/kg Nivo q2w plus 1 mg/kg Ipil q6w; N1I3, 1 mg/kg Nivo plus 3 mg/kg Ipi; bTMB, blood tumor mutation burden; mut/mb, mutations per megabase; chemo, chemotherapy; Trem, Tremelimumab (anti-CTLA-4); Durv, Durvalumab (anti-PD-1); Triago, Tiragolumab (anti- TIGIT); Atezo, Atezolizumab (anti- PD-L1). **b** A forest plot charting the survival outcomes of BSABs and ICIs combination therapies in patients with lung cancer. **c** A histogram depicting the incidence of grade ≥ 3 AEs, as well as major compositions of grade ≥ 3 TRAEs in patients with lung cancer treated with BSABs and ICIs combination therapies. A bar with a value of 0 means not mentioned in the article

Amivantamab treatment has shown significant antitumor activity in patients with EGFR ex20ins mutations compared to other antitumor therapies. In lung cancer, the research population for the investigation of BSABs mainly consists of patients with advanced or metastatic lung cancer who have progressed after first-line chemotherapy, with all of them demonstrating a potential response rate. Bintrafusp alfa and AK112 have better outcomes in patients with high PD-L1 expression levels (ORR, 85.7% and 56.3%, respectively) [85, 91]. Patients with lung squamous cell carcinoma undergoing AK112 and KN046 treatment exhibited greater survival benefits (AK112: ORR, 77.8%, 6-month PFS rate, 83.3%; KN026: mPFS, 7.3 months, 9-month OS rate, 88.2%) [86, 91]. Similarly, the ICI combination therapies that inhibited the CTLA-4 and PD-1 signaling pathways also resulted in improved response rates and survival benefits (ORR, 37.3–45.3%; 5-year OS rate, 19–24%), particularly in patients with high TMB (ORR, 45.3%; mOS, 21.9 months) [99–102]. ICI combination therapies combined with limited chemotherapy (mOS, 15.6 months; 3-year OS rate, 27%) as well as ICI combination therapy regimens as neoadjuvant therapies (MPR rate, 38%; pCR rate, 29%) have shown promising therapeutic efficacy [104–107]. ICI combination therapies have also exhibited enhanced clinical effectiveness in treating SCLC (ORR, 25%; 1-year OS rate, 42%), with relatively greater benefits in patients with high tumor burden (mOS, 10.7 months) [110]. Moreover, the anti-tumor efficacy of ICI combination therapies is higher than that of early treatment with BSABs (Fig. 3a, b and Supplementary Table S3). This finding may be attributed to the differences in the enrolled patient populations. The research on ICI combination therapies primarily included patients with advanced cancer and not previously received treatment. In contrast, the patient population in BSAB therapy studies comprised patients who harbored driver genes and had multiple treatment failures. Furthermore, bintrafusp alfa showed promising efficacy in treating NSCLC in the early stage. However, it could not provide similar effectiveness as PD-1 inhibitors in subsequent analyses, leading to the discontinuation of its further development. Nevertheless, research on blocking the PD-L1/TGF-β signaling pathway in lung cancer treatment remains a promising strategy. The AEs associated with BSABs have been shown to have a lower incidence rate than ICI combination therapies, and they primarily consist of target-related AEs. Conversely, the main AEs associated with ICI combination therapies include elevated lipase levels, rash, diarrhea/enteritis, and injection-related AEs (across all grades) (Fig. 3c and Supplementary Table S3).

## Digestive system tumors

### Peritoneal carcinoma

Peritoneal carcinomas commonly occur as a relapse or metastasis of gastric cancer, often resulting in poor survival rates and deterioration in quality of life. Currently, no effective treatments are available for most cases of advanced peritoneal carcinoma [111].

#### Bispecific antibodies

The epithelial cell adhesion molecule (EpCAM) participates in cell adhesion, proliferation, and differentiation. EpCAM expression has been established in various normal epithelial tissues and cancers, with its overexpression being particularly observed in colorectal, gastric, ovarian, and prostate cancers. Considering that peritoneal cells originate from the mesothelium and consequently do not express EpCAM, EpCAM may serve as a potential therapeutic target for peritoneal cavity tumors [112]. Catumaxomab is a trifunctional BSAB that targets EpCAM and CD3, acting as a bridge between the tumor and T cells. Furthermore, its retained Fc domain can activate other immune cells to participate in eliminating the tumor [113]. Catumaxomab has been approved for treating malignant ascites in patients with EpCAM^+^ cancer. Goere et al. investigated the effects of catumaxomab on the leukocytes in malignant ascites and found that its administration elicited anti-tumor activity by enhancing T-cell activation, promoting tumor cell death, and synergizing with oxaliplatin to strengthen the anti-tumor effect [114]. Moreover, a phase I/II trial by Ströhlein et al. determined the MTD of catumaxomab for treating patients with pancreatic cancer (10/20/50/200 mg at day 0/3/7/10, respectively). Among the patients enrolled, 65% showed no progression (*n* = 11/17; ORR, 23.5%; CR, one patient; PR, three patients), while the mOS from the diagnosis of pancreatic cancer was 16.7 months. In the case of AEs, the most common adverse reactions associated with catumaxomab were gastrointestinal reactions caused by CRS and injection-site reactions. Additionally, most patients received subsequent chemotherapy. Ströhlein et al. further conducted a paired analysis with patients who only received palliative chemotherapy and reported an mOS of 6 months in this patient group. Furthermore, patients treated with catumaxomab demonstrated a significant survival benefit compared to those who received palliative chemotherapy (hazard ratio [HR], 0.421) [115]. A research study by Knödler et al. suggested that combining catumaxomab with systemic chemotherapy might be a treatment approach worth exploring for patients with advanced gastric cancer, with the study reporting an mCR of 27% and an mOS of 13.2 months in this patient population [116]. The strategy of using catumaxomab for treating patients with EpCAM^+^ and platinum-resistant epithelial ovarian cancer remains under investigation. In phase II and IIa trials, catumaxomab combined with cytoreductive surgery or monotherapy exhibited certain activity in platinum-resistant ovarian cancer (24-month OS rate, 85%; mOS, 185 days) [117, 118].

### Gastric, esophageal, and GEJ adenocarcinomas

The prognosis for patients with esophageal cancer is generally poor, with most patients already in the advanced stage (5-year survival rate < 10%) [119]. Advanced gastric cancer is primarily treated with sequential chemotherapy regimens, which have improved the survival rate and quality of life of patients in the advanced stage (mOS < 1 year). However, treating gastric cancer remains a therapeutic challenge. Currently, targeted therapies for patients with locally advanced unresectable or metastatic gastric cancer mainly consist of anti-HER2 Mabs, anti-angiogenic drugs, and PD-1 inhibitors [120]. Moreover, patients with gastric/gastroesophageal junction (G/GEJ) adenocarcinoma are often diagnosed at an advanced or metastatic stage. The first-line therapies for patients with G/GEJ adenocarcinoma typically involve platinum-based chemotherapy, anti-angiogenic drugs, and ICIs, which have shown ORRs ranging from 25 to 56.7% and mOS varying from 10.4 to 17.5 months. However, the recurrence rates following these therapies remain high [121].

#### Bispecific antibodies

Preclinical studies have revealed that TGF-β expression can induce EMT in esophageal cancer. Additionally, elevated TGF-β expression was associated with advanced stage esophageal adenocarcinoma [122]. In patients with advanced esophageal adenocarcinoma treated with bintrafusp alfa (a PD-L1/TGF-β receptor II [TGF-βRII] BSAB), 83.3% of the tumors exhibited an immune-excluded phenotype, along with a promising clinical response (ORR, 20.0%; 12-month OS rate, 32.1%). In contrast, the phase II and III trials of pembrolizumab Mab for esophageal adenocarcinoma have reported lower ORRs of 3–18% and 12-month OS rates of 22–24%. Thus, bintrafusp alfa represents a potential new treatment option for patients with platinum-resistant esophageal adenocarcinoma [123].

Carcinoembryonic antigen (CEA) is highly expressed in many gastrointestinal cancers, such as colorectal, pancreatic, and gallbladder cancers [124]. The elevated levels of soluble CEA are linked with cancer progression, making CEA a key target antigen for the development of colon cancer and other tumor antigens. MEDI-565 is a CEA/CD3 BSAB that has shown anti-tumor activity in preclinical experiments, including inducing cytotoxic T-cell killing of tumors in the presence of high levels of free CEA [125]. A phase I trial of MEDI-565 identified an MTD of 5 mg in patients with gastric adenocarcinoma, with the best clinical response attained in 28% of the patients with stable disease. The researchers further hypothesized that the intermittent infusion of MEDI-565 may result in decreased drug concentration. In light of this hypothesis, a continuous infusion protocol is currently being evaluated (Supplementary Table S4) [126].

HER2 overexpression/amplification is frequently detected in digestive system tumors (particularly in G/GEJ cancer) and is correlated with a poor prognosis [127]. Thus, HER2 may serve as a potential driver and biomarker of gastric cancer. Moreover, the combined treatment of trastuzumab (a HER2 Mab) with chemotherapy has been shown to improve the OS (13.8 months) and ORR (47%) of patients with advanced G/GEJ cancer [128]. Based on these results, trastuzumab has been approved as a first-line treatment for HER2^+^ patients with gastric cancer. KN026 is a BSAB targeting the HER2 binding domains (extracellular domain 2 [ECD2] × ECD4), which can enhance antibody binding to HER2. In a study of 30 patients with G/GEJ cancer who had failed first-line therapies, an ORR of 55.6% was demonstrated in those with high HER2 expression, while an ORR and mOS of 44.4% and 5.6 months and 22.2% and 9.6 months were observed in those who had previously received HER2-targeted therapy and those with low HER2 expression, respectively [129]. A subsequent phase II trial of the patients with high HER2 expression (as mentioned above) revealed an mOS of 16.3 months and an mPFS of 8.3 months. Furthermore, patients with low HER2 expression had a final ORR of 14%. However, treatment-related adverse effects occurred at a higher incidence (82%, grade ≥ 3 AEs: 8%), mainly characterized by AEs such as elevated liver enzyme levels, rash, and anemia [130]. Another BSAB, zanidatamab (ZW25), which targets the same antigenic epitope as KN026, was applied in a solid tumor cohort of 11 patients with G/GEJ cancer. Zanidatamab exhibited some anti-tumor activity (DCR, 57%; PR, 43%) in these patients [131]. The combined treatment regimen of chemotherapy and tislelizumab with zanidatamab as a first-line therapy has also demonstrated good anti-tumor activity (ORR, 72.7%; mPFS, 10.9 months) in patients with locally advanced or metastatic G/GEJ adenocarcinoma [132]. Furthermore, early studies have highlighted a synergistic effect between ICIs and chemotherapy, leading to improved survival in patients with advanced cancer. The researchers observed good therapeutic efficacy after administering AK104 (a PD-1/CTLA-4 BSAB) in combination with XELOX (capecitabine plus oxaliplatin) or modified XELOX in patients with unresectable advanced G/GEJ cancer. The therapeutic outcomes included an ORR of 65.9% (*n* = 96; CR, 2.3%; PR, 63.6%), mPFS of 7.10 months, and mOS of 17.41 months [133].

#### ICI combination therapies

A treatment regimen involving nivolumab in conjunction with chemotherapy or ipilimumab was investigated in a patient population with untreated advanced esophageal squamous cell carcinoma (ESCC). Both combination treatments exhibited favorable clinical benefits and sustained response rates compared to chemotherapy alone (nivolumab + chemotherapy, nivolumab + ipilimumab, and chemotherapy alone; ORR: 47%, 28%, and 27%; ≥ 12-month DoR rates, 39%, 48%, and 23%). The results of these treatment strategies present a potential avenue for developing first-line therapies for advanced ESCC [134, 135]. GEJ adenocarcinoma is usually associated with chronic inflammation, high microsatellite instability, high TMB, and excessive expression of immune checkpoint proteins, with related research suggesting that ICIs may be a viable therapeutic strategy in this cancer type [136]. Another study also compared the clinical benefits of nivolumab monotherapy with those of nivolumab plus ipilimumab in patients with locally advanced or metastatic GEJ cancer. The study findings demonstrated that the combined treatment of nivolumab (1 mg/kg) with ipilimumab (3 mg/kg) achieved higher ORR and survival benefits than nivolumab administration alone (ORR, 24% vs. 12%; mOS, 6.9 vs. 6.2 months). However, the combined treatment led to a higher incidence of grade 3/4 AEs than nivolumab monotherapy, with the AEs mainly including diarrhea and elevated liver enzyme levels [137]. In contrast, a multicenter phase III study evaluating the use of combined nivolumab (1 mg/kg) and ipilimumab (3 mg/kg), chemotherapy alone, or nivolumab combined with chemotherapy as a first-line treatment in patients with advanced GEJ adenocarcinoma reported no significant improvement in the OS and ORR (nivolumab + ipilimumab vs. chemotherapy: 2-year OS rate, 25% vs. 19%; ORR, 23% vs. 47%). Nevertheless, the ICI combination therapy did result in an extended mDoR compared to chemotherapy alone (13.8 vs. 6.8 months). The contradiction in the outcomes between the two previously mentioned studies may be attributed to multiple factors. Further subgroup analysis revealed that ICI combination therapies (nivolumab plus ipilimumab) in patients with microsatellite instability-high (MSI-H) adenocarcinoma yielded longer mOS (HR, 0.28) and higher ORR (70% vs. 57%) than chemotherapy in patients with MSI-H tumor. However, these results were not reflected in PD-L1-positive patients, with no improvement in the ORRs between the ICI combination therapy and chemotherapy groups (27% for ICI combination therapy vs. 47% for chemotherapy) [138].

A study by André et al. employed nivolumab plus ipilimumab for the perioperative treatment of patients with deficient mismatch repair (dMMR)/MSI-H G/GEJ adenocarcinoma. The research findings indicated that the combined treatment of nivolumab plus ipilimumab as a neoadjuvant therapy is feasible in this patient population, with no unexpected toxicity and a high pathological response rate in the tumor tissue (pCR rate, 58.6%) [139].

### Biliary tract cancer

The primary treatment for biliary tract cancer (BTC) is surgical intervention. However, the early symptoms of this cancer are not obvious, resulting in most patients being diagnosed at an advanced stage. This delayed diagnosis leads to many missed opportunities for surgery. Consequently, the OS of patients with advanced or metastatic BTC is low (mOS, 2.5–4.5 months). These patients predominantly undergo combination chemotherapy with gemcitabine and platinum (mOS, 11.2 months) [140]. However, the therapeutic options for patients who experience disease progression after first-line chemotherapy are limited. Moreover, the ORR of the second-line chemotherapy regimen has been reported to be < 10%, with an mOS of < 6 months [141].

#### Bispecific antibodies

A study by Mondaca et al. revealed that approximately 5.4% of patients with BTC exhibit HER2 overexpression/amplification, with these patients tending to have faster tumor progression [141]. The early clinical data of zanidatamab (ZW25, a BSAB against HER2 domains ECD2 and ECD4) at a dose of 20 mg/kg (q2w) for treating patients with BTC with high HER2 expression showed promising results. The achieved ORR was 47%, while TRAE incidence was 70%, along with a favorable DCR [142].

### Hepatocellular carcinoma

Hepatocellular carcinoma (HCC) poses considerable diagnostic challenges and frequently progresses to the advanced stage, where transplantation or resection is unfeasible. Approximately 50% of the patients with HCC require systemic therapy, which is compounded by an increasing risk of poor prognosis, morbidity, and mortality over time [143, 144]. Currently, the first-line treatment, predominantly involving sorafenib or lenvatinib administration, achieves an mOS of 11–14 months, whereas the second-line therapies yield an OS of 8–11 months [145]. Other research studies have revealed that immunotherapy may benefit patients with HCC. For example, recent investigations have discovered the efficacy of combining ICIs with multi-kinase inhibitors in patients with advanced HCC. Additionally, PD-1 inhibitors have shown good curative efficacy in treating advanced liver cancer (mOS, 13.9 months) and have been approved as a second-line therapy [144].

#### Bispecific antibodies

Zhou et al. proposed a treatment protocol involving the administration of 6 mg/kg of AK104 (a PD-1/CTLA-4 BSAB) in combination with lenvatinib as a first-line therapy for patients diagnosed with unresectable advanced HCC. The study showed that this protocol had promising anti-tumor efficacy (ORR, 44.4%; DCR, 77.8%) and manageable drug toxicity (grade ≥ 3 TRAEs, 26.7%) [146].

#### ICI combination therapies

A trial was mainly conducted in patients with advanced liver cancer who had failed sorafenib treatment. The results indicated that 1 mg/kg of nivolumab plus 3 mg/kg of ipilimumab (q3w for four doses) followed by 240 mg of nivolumab (q2w) facilitated a higher OS, with no association with the disease etiology (ORR, 32%; mOS, 22.2 months; 36-month OS rate, 42%). However, TRAE incidence was high (grade ≥ 3 TRAEs, 53%), presenting as rash, hepatitis, and adrenal insufficiency [147, 148]. This treatment approach has received approval in the United States as a second-line therapy for advanced HCC. Another study examined the use of a single dose of tremelimumab (300 mg) combined with durvalumab (1500 mg) as a maintenance treatment to reduce treatment toxicity, and the results showed an ORR of 24% and an mOS of 18.7 months. In the case of AEs, the main treatment toxicities observed were elevated liver enzyme levels, increased lipase concentration, and diarrhea (grade ≥ 3 TRAEs, 37.8%). Moreover, this therapeutic combination reduced the need for glucocorticoid treatment[149]. Subsequently, this treatment regimen was employed in the phase III trial of the HIMALAYA study, which included 1,171 patients with advanced HCC. These trial findings further confirmed the superior efficacy and durable survival benefit correlated with combining a single dose of tremelimumab with durvalumab compared to sorafenib (ORR, 20.1% vs. 5.1%; 36-month OS rate, 30.7% vs. 20.2%) [150].

### Pancreatic vancer

The 5-year survival rate for advanced pancreatic ductal adenocarcinoma is currently low at 8.5%. However, the existing treatment strategies have not yet improved the survival rate, indicating that new treatment methods are urgently required [151].

#### Bispecific antibodies

Although NRG1 gene rearrangement is uncommon in pancreatic cancer, it tends to be enriched in younger patients with KRAS wild-type pancreatic cancer [152]. In the eNRGy trial conducted by Schram et al., a cohort of 18 patients with pancreatic cancer received treatment with zenocutuzumab (MCLA-128), resulting in an ORR of 39% (34% ORR in NRG1^+^ tumors) [96]. Currently, zenocutuzumab is designated as an orphan drug for pancreatic ductal adenocarcinoma and is on a fast-track approval process for use as standard therapy in advanced NRG1^+^ cancers.

### Colorectal cancer

Patients with dMMR/MSI-H metastatic colorectal cancer (mCRC) have a worse prognosis after conventional chemotherapy. Their OS is shorter than that of patients with proficient mismatch repair (pMMR) mCRC (mOS, 13.6 vs. 16.8 months) [153]. Furthermore, most pMMR/microsatellite stable (pMMR/MSS) mCRCs exhibit immune exclusion with intrinsic resistance to ICIs [154].

#### ICI combination therapies

Studies have revealed that dMMR/MSI-H colorectal cancers show elevated mutational burden, tumor neoantigen burden, and increased immune infiltration of CD8^+^ T cells[155]. These characteristics indicate the potential for immune checkpoint-targeted immunotherapy in tumors with dMMR. In line with this notion, pembrolizumab, which blocks the PD-1 signaling pathway, was employed in a phase II trial in patients with dMMR mCRC. The trial reported clinical benefits associated with pembrolizumab treatment (ORR, 50%; DCR, 89%; 24-month OS rate, 66%; 24-month PFS rate, 61%) [156]. Checkmate 124, a multicenter phase II study, explored the application of ICIs, i.e., nivolumab monotherapy or nivolumab in combination with ipilimumab, in patients with dMMR/MSI-H mCRC. The study primarily included patients with dMMR/MSI-H mCRC who experienced disease progression after first-line therapies, yielding an ORR of 55% and 31.1% in the combination therapy and nivolumab monotherapy groups, respectively [157, 158]. The 5-year follow-up analysis of these two therapies reported a significant and durable survival benefit for the combination of nivolumab and ipilimumab compared to nivolumab monotherapy (ORR, 65% vs. 39%; 48-month OS rate, 71% vs. 49%). Additionally, the combination of nivolumab (3 mg/kg, q2w) with low-dose ipilimumab (1 mg/kg, q6w) demonstrated improved antitumor response rate and safety compared to nivolumab alone (ORR, 71% vs. 39%, grade ≥ 3 TRAEs, 27% vs. 20%). Consequently, the researchers suggest that the combined treatment regimen of nivolumab with ipilimumab may serve as a first-line therapy for patients with dMMR/MSI-H mCRC [159].

The methylation of the O6-methylguanine-DNA methyltransferase (MGMT) promoter is an early event in colorectal cancer, occurring in approximately 40% of patients with colorectal cancer. Temozolomide treatment in patients with this MGMT promoter methylation has demonstrated modest activity (ORR, 10%) [160]. Furthermore, researchers postulate that the hypermutation induction by temozolomide provides an opportunity for immunotherapy in patients with pMMR/MSS and MGMT-silenced mCRCs. The MAYA trial implemented an ICI combination therapy comprising temozolomide initiation followed by low-dose ipilimumab and nivolumab in patients with pMMR/MSS and MGMT-silenced mCRCs. The trial results indicated sustained clinical effectiveness of this therapy, with an ORR of 45% and an mOS of 18.4 months. Moreover, the grade ≥ 3 immune-related AEs primarily consisted of rash, colitis, and hypophysitis [154].

**Fig. 4:**
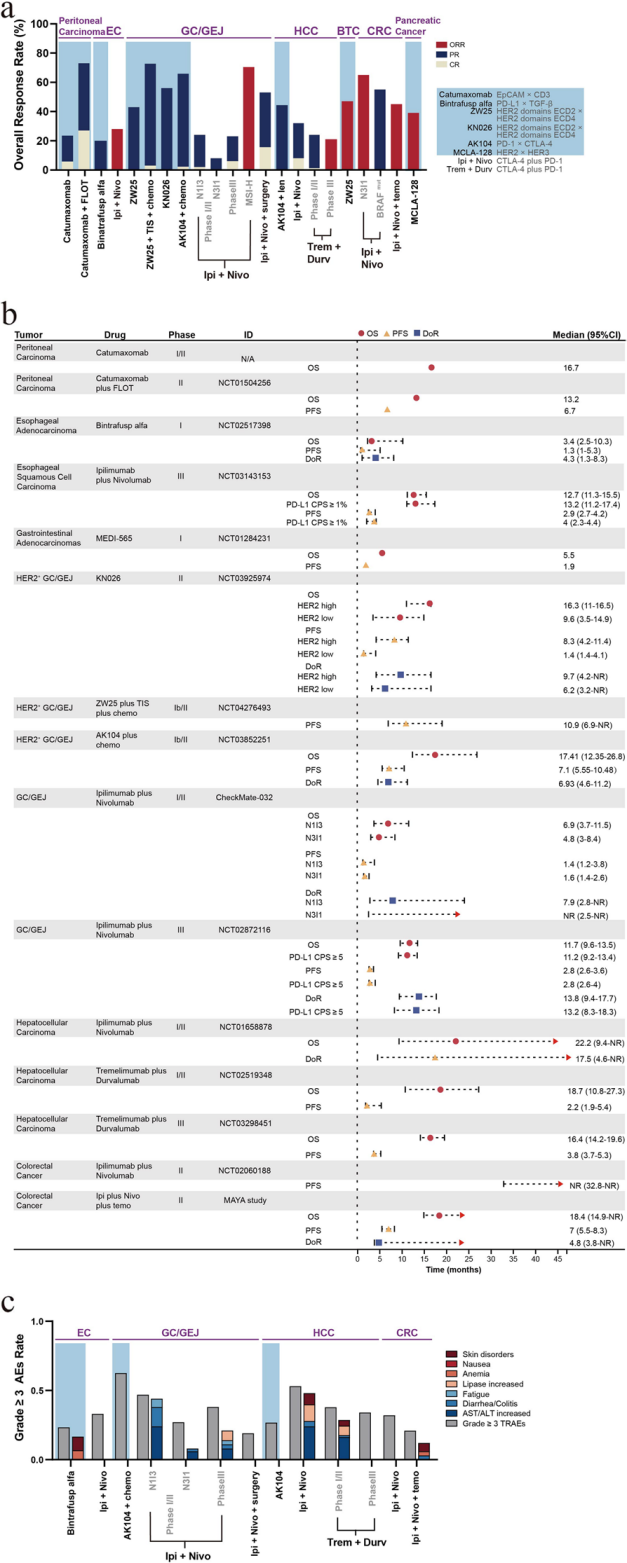
Efficacy, adverse events, and survival outcome of bispecific antibody treatment and ICI combination therapies in patients with Digestive system tumors. **a** A histogram depicting the antitumor activity of BSABs and ICIs combination therapies in in patients with digestive system tumors. EC, esophageal cancer; HCC, hepatocellular carcinoma; BTC, biliary tract cancer; CRC, colorectal cancer; AK104, Cadonilimab; MCLA-128, Zenocutuzumab; ZW25, Zanidatamab. The ORR, defined as a sum of CR and PR. **b** A forest plot charting the survival outcomes of BSABs and ICIs combination therapies in patients with digestive system tumors. PD-L1 CPS, PD-L1 combined positive score. **c** A histogram depicting the incidence of grade ≥ 3 TRAEs, as well as major compositions of grade ≥ 3 TRAEs with BSABs and ICIs combination therapies in patients with digestive system tumors. A bar with a value of 0 means not mentioned in the article

Overall, the primary targets of BSABs in gastrointestinal cancers are EpCAM, CEA, HER2, immune checkpoints, and TGF-β. The application of catumaxomab or catumaxomab combined with chemotherapy has improved the survival of patients with advanced peritoneal cancer (mOS, 13.2–16.7 months) [115, 116]. In the case of patients with esophageal cancer, ICI combination therapies demonstrated a higher antitumor response rate than bintrafusp alfa (ORR, 47% vs. 20%). However, the efficacy of BSABs against PD-L1/TGF-β still requires further evaluation [123]. HER2 is overexpressed/amplified in certain patients with gastrointestinal tumors. The application of HER2-targeting BSABs has exhibited promising efficacy in patients with G/GEJ cancers (ORR, 44.4–55.6%) or BTC (ORR, 47%) who presented with high HER2 expression or previously received HER2-targeted therapy [129–131, 142]. ICI combination therapies can elicit a significant response rate (ORR, 70%) and prolonged DoR in patients with advanced G/GEJ cancer, particularly in MSI-H patients [137, 138]. However, the survival improvement may not be as efficient as that obtained with HER2 BSABs (mOS, 4.8–11.7 vs. 16.3–17.4 months) [129, 130, 133]. Furthermore, ICI combination therapies as neoadjuvant therapy have demonstrated certain clinical efficacy in resectable dMMR/MSI-H G/GEJ adenocarcinoma (pCR rate, 58.6%)[139]. ICI combination therapies that inhibit the CTLA-4 and PD-1 signaling pathways have achieved remarkable efficacy in patients with HCC (ORR, 20.1–32%; mOS, 18.7–22.2 months; 36-month OS rate, 30.7–42%) [147–150]. However, the response rate (ORR, 44.4%) of CTLA-4/PD-1 BSABs combined with lenvatinib was higher than that of the ICI combination therapies. ICI combination therapies also exhibited improved prognosis in patients with dMMR/MSI-H mCRC (ORR, 55–71%; 48-month OS rate, 71–72%), while ICI combination therapies accompanied with temozolomide administration produced sustained clinical efficacy (ORR, 45%; mOS, 18.4 months) in patients with pMMR/MSS mCRC (Fig. 4a, b and Supplementary Table S4) [154]. Additionally, the incidence of grade 3 AEs associated with BSAB treatment in HCC and colorectal cancer requires further evaluation. Considering this, some researchers have examined the adjustment of a combined dose of ICIs to mitigate treatment-related toxicities (grade ≥ 3 TRAEs, 19–53%), particularly reducing the dose of CTLA-4 Mab (grade ≥ 3 TRAEs, 34–37.8% in HCC; grade ≥ 3 TRAEs, 20% in colorectal cancer) [149, 150, 159]. Among the AEs, elevated liver enzyme levels, increased lipase concentration, and skin rash are mainly observed (Fig. 4c and Supplementary Table S4).

### Breast cancer

Early diagnosis and comprehensive therapeutic strategies for breast cancer (BC) have improved prognoses among these patients. However, metastatic BC treatment is limited to palliative interventions, with a 5-year OS rate of only 25% [161]. The mOS is 17–20 months after the first observation of metastasis, thus indicating the need to develop novel therapeutic approaches to enhance the survival rates of patients with metastatic BC and failed HER2-targeted therapy [162].

#### Bispecific antibodies

HER2/neu, a member of the epidermal growth factor family, is highly expressed in approximately 25–30% of BC cases, with its increased expression being associated with tumor aggressiveness and poor prognosis [162]. HER2 overexpression has been reported to elicit cell proliferation, transformation, and tumor growth, as well as inhibit tumor apoptosis [163]. Trastuzumab combined with chemotherapy yields notable clinical benefits in HER2-positive metastatic BC, extending the mOS to 25.1 months [164]. Ertumaxomab is a BSAB that targets HER2 and CD3. In vitro studies have shown that ertumaxomab at low concentrations can kill tumor cells at a rate of 97–99%, even in the presence of elevated trastuzumab levels. Furthermore, ertumaxomab can eliminate tumor cells where HER2/neu expression is low (1+), potentially offering a novel therapeutic strategy for patients with BC who are unsuitable candidates for trastuzumab treatment [165]. Considering these results, a study examined the effects of ertumaxomab administration in patients with metastatic BC who expressed HER2/neu. The research determined the MTD as 100 µg, while the clinical benefit rate (CBR) was 33%. Moreover, the toxicity and side effects of ertumaxomab treatment were controllable and safe, with AEs predominantly characterized by fever, generalized rigidity, and headache [166]. Zanidatamab (ZW25) is a BSAB targeting the extracellular and membrane domains of HER2, i.e., ECD4 and ECD2. Compared to Mabs, such as trastuzumab and pertuzumab, zanidatamab can enhance the binding affinity between the antibody and HER2. Additionally, this BSAB can reduce the internalization and downregulation of HER2 receptors. Furthermore, zanidatamab exhibits ADCC and retains similar activity even in patients with low HER2 expression levels [167]. In a population of patients with BC who have undergone various treatments, zanidatamab treatment led to a DCR of 54% [131]. In another study of patients with advanced BC, zanidatamab combined with 75 mg/m^2^ of docetaxel as first-line therapy resulted in an ORR of 86.4% and a 6-month PFS rate of 90.9%, highlighting its significant anti-tumor activity [168]. Furthermore, KN026, a BSAB targeting the same epitope as zanidatamab, demonstrated encouraging antitumor activity in 63 patients with metastatic BC who failed HER2-targeted therapy (KN026 dose: 20 mg/kg, q2w and 30 mg/kg, q3w), with a DCR of 76.8% and an ORR of 32.1% [169].

NRG1 rearrangements have been revealed to occur in patients with BC [93]. A phase I trial of zenocutuzumab (MCLA-128) showed promising clinical activity (CBR, 70%) in a cohort of patients with NRG1^+^ metastatic BC. In the case of AEs, fatigue, anemia, and diarrhea AEs were mainly observed, with a rare occurrence of grade 3/4 events [170]. Furthermore, an estrogen receptor ER^+^/HER2-low BC model has revealed the presence of a bidirectional crosstalk between the ER and HER2/HER3 axis, which can result in resistance to endocrine therapy (ET). Moreover, the activation of the NRG1-HER3 ligand and HER2/HER3 was found to cause ER phosphorylation, consequently upregulating HER2 and HER3 expression. The study also suggested that compared to ET alone, zenocutuzumab combined with ET may elicit a superior antitumor efficacy [171]. Based on this finding, a phase II trial was conducted for patients with endocrine-resistant ER^+^/HER2-low BC that had progressed after cyclin-dependent kinase 4 and 6 inhibitor (CDK4/6i) therapy. In this trial, a combination treatment of zenocutuzumab with ET (fulvestrant or aromatase inhibitors) was administered in 42 patients, achieving a clinical response rate of 45% (PR, two patients; stable disease [SD], 17 patients) [172]. Furthermore, a triple therapy comprising zenocutuzumab in combination with trastuzumab and vinorelbine also yielded promising efficacy in patients with metastatic BC who have undergone multiple treatments (DCR, 77%) [173].

**Fig. 5:**
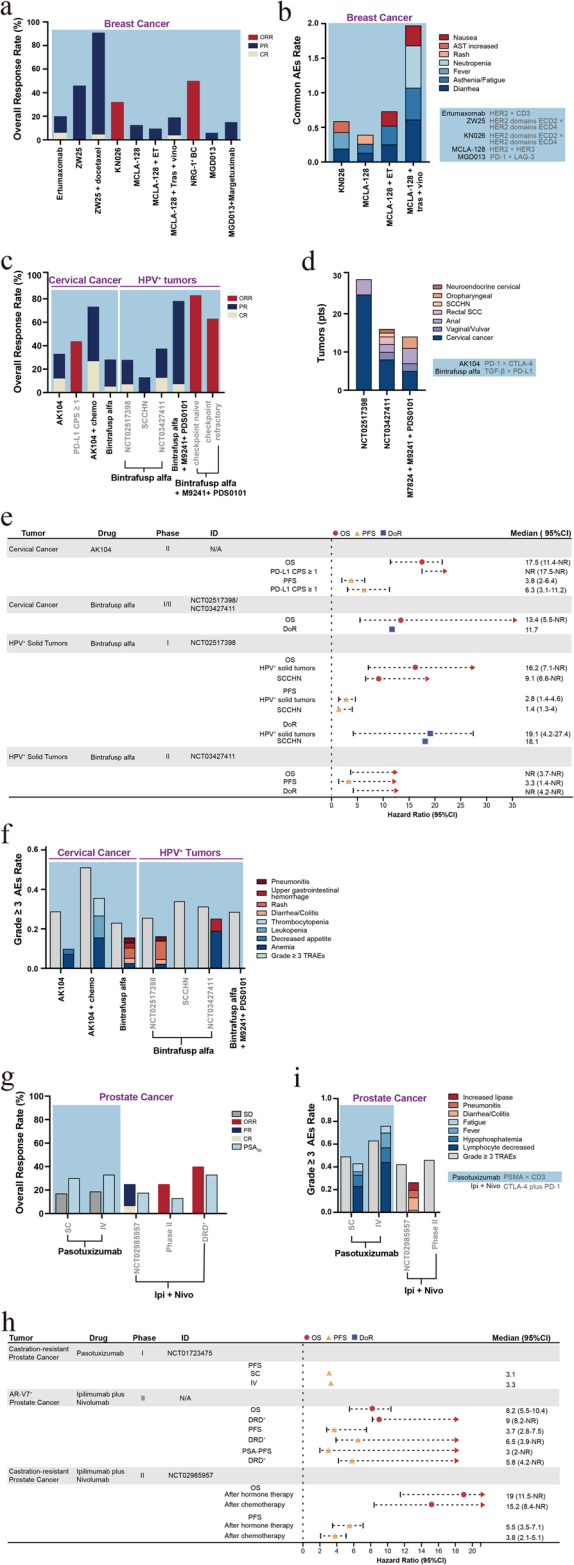
Efficacy, adverse events, and survival outcome of bispecific antibody treatment and ICI combination therapies in patients with sex-related tumors. A histogram of the antitumor activity (**a**) and compositions of major tertiary AEs (**b**) for BSABs in patients with breast cancer. A histogram of the antitumor activity (**c**) of BSABs for HPV + tumors and the tumor categories primarily recruited in several trials (**d**). ET, endocrine therapy; Tras, Trastuzumab (anti-HER2); vino, vinorelbine; M9241 (anti-IL-12); PDS0101, peptide vaccine targeting E6/E7 proteins. A forest plot charting the survival outcomes (**e**) and a histogram depicting the incidence of grade ≥ 3 TRAEs, as well as major compositions (**f**) of BSABs in patients with HPV-positive tumors. Histogram of antitumor activity (**g**) and a forest plot of survival outcomes (**h**) and incidence of grade ≥ 3 TRAEs, including major components (**i**) of BSABs and ICIs combination therapies in patients with prostate cancer. SD, stable disease; PSA 50 , percentage of people with a PSA decline rate of 50% or higher; DRD, DNA repair defects. The ORR, defined as a sum of CR and PR. A bar with a value of 0 means not mentioned in the article

In summary, the use of HER2/CD3 BSABs in treating patients with BC has resulted in a DCR of 33%, along with promising early antitumor activity from BSABs targeting the HER2 domains ECD2 and ECD4 in patients with metastatic BC who had failed multiple prior treatments (DCRs of 54% and 76.8%) [131, 169]. Furthermore, zanidatamab combined with docetaxel showed more significant antitumor activity than zanidatamab monotherapy (ORR, 86.4%; 6-month PFS rate, 90.9%) [168]. Additionally, the efficacy of HER2/HER3 BSABs in patients with NRG^+^ BC (CBR, 70%) was comparable to that obtained by BSABs with dual targeting of the HER2 epitope [170]. The combination of ET or trastuzumab with vinorelbine can also yields certain curative effects in patients with metastatic BC that had progressed following CDK4/6i treatment or experienced multiple treatment failures (CBRs of 45% and 77%) [172, 173]. Additionally, PD-1/LAG-3 BSABs have shown preliminary efficacy in triple-negative BC (DCR, 45%; PR, 6%). In combination with Margetuximab (a HER2 Mab), they have also demonstrated favorable therapeutic effects in breast cancer patients previously received anti-HER2 therapy (*n* = 4/30; ORR, 13.3%; Fig. 5a and Supplementary Table S6) [174]. Lastly, many studies have reported manageable and safe toxicities in patients with BC treated with BSABs, with AEs including diarrhea, fatigue, and nausea/vomiting (Fig. 5b and Supplementary Table S6).

### HPV^+^ tumors

Many patients with early-stage cervical cancer can be cured via radical surgery or chemoradiotherapy. However, patients with high-risk factors or those experiencing recurrence/metastasis have a poor prognosis. The application of platinum-based chemotherapy combined with bevacizumab has shown some improvement in patients with advanced cervical cancer (mOS, 17 months) [175]. More than 95% of cervical cancer cases are associated with human papillomavirus (HPV) infection, wherein the HPV integrates into the cellular genome to suppress the immune system and promote tumor survival and immune evasion. Furthermore, studies have shown that PD-L1 is widely expressed in the TME of cervical cancer, suggesting that immunotherapy is an effective approach for cervical [176]. HPV infection is one of the main risk factors for head and neck squamous cell carcinoma [177]. Pembrolizumab combined with a platinum agent and 5-fluorouracil is a first-line treatment option for patients with a combined positive score (CPS) > 1 or recurrent/metastatic head and neck squamous cell carcinoma (mOS, < 1 year). Additionally, nivolumab or pembrolizumab as second-line therapies in these patients exhibit an ORR of 13–16%, with an even lower OS [178–180].

#### Bispecific antibodies

Cadonilimab (AK104) is a BSAB that simultaneously targets the PD-1/CTLA-4 signaling pathways, with a propensity for selectively binding to TILs co-expressing PD-1 and CTLA-4 in the TME. Moreover, AK104 facilitates the internalization of PD-1 and CTLA-4, further reducing their expression on the cellular membrane. Additionally, compared to ICI combination therapies, AK104 demonstrates higher safety (source: https://www.akesobio.com/cn/media/akeso-news/211115-3/). This BSAB was investigated in patients with advanced cervical cancer who had not received immunosuppressive therapy. The study results revealed that regardless of tumor PD-L1 expression, AK104 achieved efficacy (ORR, 33.0%; CR, 12.0%; mOS, 17.5 months) and safety (grade ≥ 3 TRAEs, 28.8%) that were comparable to those of second-line therapies for recurrent/metastatic cervical cancer. Moreover, patients with PD-L1 positivity exhibited superior clinical benefits (ORR, 43.8%; 12-month OS rate, 64.4%) [181]. Furthermore, a phase II trial combining AK104 with platinum-based chemotherapy +/− bevacizumab as a first-line therapy revealed an ORR of 73.3% for AK104 (15 mg/kg) with platinum-based chemotherapy and an ORR of 92.3% for AK104 plus platinum-based chemotherapy combined with bevacizumab. However, an increased incidence of treatment-related toxicities was observed in the combined treatment compared to the trial of AK104 monotherapy (grade ≥ 3 TRAEs, 51.1% for AK104 combined treatment vs. 28.8% for AK104) [181, 182]. Moreover, AK104 has been granted FDA fast-track designation and orphan drug status as well as been recognized as a breakthrough therapy by the National Medical Products Administration in China.

 Research has indicated that the E6 and E7 oncoproteins of the HPV can activate the TGF-β promoter in cervical cancer cell lines [183]. Furthermore, studies have found that patients with HPV-related head and neck squamous cell carcinoma and oropharyngeal cancer exhibit overexpression of TGF-βR1 or enrichment of TGF-β genes, which correlates with the disease prognosis. Consequently, the TGF-β signaling pathway may serve as a potential target for HPV^+^ tumors [184, 185]. The TGF-β signaling pathway is crucial in various tumorigenesis processes within the TME, as well as enhances fibrosis, angiogenesis, and EMT to promote tumor resistance and metastasis. Furthermore, TGF-β facilitates Treg differentiation and suppresses their antitumor response. TGF-β has also been found to modulate the NK cell phenotype, inhibiting their cytotoxic activity [186, 187]. Preclinical investigations have indicated that bintrafusp alfa (a PD-L1/TGF-βRII BSAB) enhances the activation, cytotoxicity, and aggregation of NK cells in the TME, thereby alleviating the antitumor response. The mechanism of bintrafusp alfa involves several key effects including: (1) impeding or reversing TGF-β-induced EMT in tumor cells, (2) changing the NK and T cell phenotypes to augment their capacity to kill tumors, (3) eliciting ADCC to enhance tumor lysis, (4) diminishing Treg activity, and (5) increasing the sensitivity of tumors to chemotherapeutic agents [188]. Preclinical studies conducted on murine models have demonstrated the robust inhibitory effect of bintrafusp alfa on tumor growth and metastasis compared to that of TGF-β and PD-L1 inhibitors [189]. Preliminary clinical research has further indicated that bintrafusp alfa can improve the outcomes in patients with HPV^+^ malignancies, including those exhibiting ICI resistance [190]. A phase I/II trial of bintrafusp alfa treatment was conducted in patients having HPV^+^ solid tumors, including cervical cancer, anal cancer, and P16^+^ head and neck squamous cell carcinoma. Among the 59 enrolled patients, an ORR of 30.5% was achieved, with 53% experiencing tumor shrinkage. Moreover, in patients refractory to ICI therapy, the ORR was 10% (*n* = 2; CR, one patient), while the mPFS and mOS were 1.4 months and 3.4 months, respectively. Furthermore, bintrafusp alfa treatment was associated with skin toxicities (27.9%) related to the TGF-β pathway blockade, including cutaneous squamous cell carcinoma, basal cell carcinoma, hyperkeratosis, and mucosal bleeding. Nevertheless, these skin lesions improved after treatment. Early experimental observations have reported a higher ORR in patients with HPV^+^ tumors than in those with HPV-negative tumors (ORR, 33% vs. 5%). Moreover, bintrafusp alfa may elicit a stronger and more durable anti-tumor response (ORR, 28.2–33%; mDoR, 2.8–30.4 months) than PD-1 inhibitors (nivolumab or pembrolizumab; ORR, 12–24%) in the treatment of HPV^+^ tumors [191, 192]. In the case of the cervical cancer cohort, an ORR of 28.2% and an mOS of 13.4 months were observed, with 20% of the patients presenting with TRAEs ≥ grade 3. Bintrafusp alfa has also shown potential clinical activity in patients with recurrent or metastatic cervical cancer who have not received ICI therapy [193].

The triple combination therapy of bintrafusp alfa, PDS0101 (a peptide vaccine targeting E6/E7 proteins), and M9241 (an immunocytokine targeting IL-12) exhibited remarkable clinical activity (ORR, 71%) in patients with advanced HPV 16^+^ tumors. Additionally, the therapy showed clinical activity in patients who were untreated or refractory to ICIs (ORR, 83% [untreated] and 63% [ICI-refractory]) [194].

PD-1/CTLA-4 and PD-L1/TGF-β BSABs demonstrate comparable efficacy (ORR, 33% and 28.2%, respectively) in patients with advanced cervical cancer who have not received immunosuppressive therapy [181, 193]. Although patients with high PD-L1 expression may benefit from PD-1/CTLA-4 BSABs (ORR, 43.8%; 12-month OS rate, 64.4%), more significant benefits are obtained via the combination therapy of PD-1/CTLA-4 BSABs with chemotherapy (ORR, 73.3%) and PD-1/CTLA-4 BSABs with chemotherapy and bevacizumab (ORR, 92.3%) [182]. In cervical cancer, PD-1/CTLA-4 BSABs may confer a longer survival period than PD-L1/TGF-β BSABs (mOS, 17.5 vs. 13.4 months; Fig. 5c, e and Supplementary Table S7). The investigation of bintrafusp alfa in treating NSCLC, BTC, and colorectal cancer was terminated due to the limited clinical efficacy; however, promising results have emerged for its application in HPV^+^ tumors (ORR, 30.5%; mOS, 9.1–16.2 months) [191, 192], including cervical cancer, vaginal/vulvar cancer, and head and neck squamous cell carcinoma (Fig. 5d). Moreover, bintrafusp alfa has shown particularly favorable efficacy when administered in combination with PDS0101 and M9241 (ORR, 63–83%; Fig. 5c, e, and Supplementary Table S7) [194]. In the case of AEs, PD-1/CTLA-4 BSABs and PD-L1/TGF-β BSABs are associated with similar incidence rates of grade ≥ 3 TRAEs (28.8% vs. 21.1–31.1%, respectively). Furthermore, the main adverse reactions in relation to PD-1/CTLA-4 BSABs are fatigue and decreased appetite, while PD-L1/TGF-β BSABs are linked with adverse reactions such as fatigue, rash, and gastrointestinal bleeding (Fig. 5f and Supplementary Table S7) [181, 192, 193].

### Prostate cancer

Castration-resistant prostate cancer (CRPC) refers to the progression of prostate cancer after androgen suppression therapy with chemotherapy or surgery. CRPC is characterized by various driving factors and resistance mechanisms, ultimately leading to a poor prognosis [195]. Prostate cancer positive for androgen receptor variant 7 (AR-V7) exhibits primary resistance to androgens because of the lack of the ligand-binding domain of the androgen receptor. Furthermore, it is potentially associated with EMT, invasiveness, and cellular proliferation. Patients with AR-V7^+^ prostate cancer are also less responsive to taxane-based drugs, exhibiting shorter OS (mOS, 7–9 months) [196].

#### Bispecific antibodies

The TME of prostate cancer is considered immunosuppressive, with previous studies suggesting that inducing T cells to infiltrate the tumor optimizes the immunotherapy effect in prostate cancer. Research has revealed that increased prostate-specific membrane antigen (PSMA) levels serve as a marker for advanced metastatic prostate cancer. The elevated PSMA levels promote tumor proliferation and inhibit tumor apoptosis, exhibiting a negative correlation with the cancer prognosis. Therefore, PSMA is a promising target for prostate cancer treatment [197]. Pasotuxizumab is a PSMA/CD3-targeting BSAB, and preclinical studies have shown that pasotuxizumab induces T cells to lyse prostate cancer cells at a median effective concentration of 0.1–4 ng/ml, leading to delayed tumor growth, tumor shrinkage, and disease remission [198]. In trials investigating the treatment of metastatic CRPC (mCRPC) with pasotuxizumab, intravenous infusion (IV) has demonstrated favorable therapeutic prospects, wherein many patients experienced a decrease in prostate-specific antigen (PSA) levels (PSA_50_, IV vs. subcutaneous [SC]: 33.3% vs. 30%). Among them, 87.5% of patients had decreased PSA concentration, with an overall change of − 20.6% compared to baseline. Moreover, a nearly complete regression of the lymph node and bone metastases was observed in one patient. However, the IV cohort was linked with higher AE incidence than the SC cohort (grade ≥ 3 TRAEs, 63% vs. 49%). The AEs in the IV group primarily manifested as lymphocytopenia and hypophosphatemia, whereas the adverse reactions in the SC group presented as lymphocytopenia and fever [199].

#### ICI combination therapies

Research studies have revealed that treating mCRPC with ipilimumab can promote immune cell infiltration and induce compensatory activation of the PD-1/PD-L1 signaling pathway. Consequently, investigators propose that blocking multiple immune checkpoints may elicit better efficacy in patients with prostate cancer [200]. Considering this notion, the use of ICI combination therapies in AR-V7^+^ metastatic prostate cancer was initially explored. In a phase II trial, 15 patients with AR-V7^+^ prostate cancer were treated with a regimen of 3 mg/kg of nivolumab plus 1 mg/kg of Ipilimumab (q3w for four doses), followed by 3 mg/kg of nivolumab (q2w). The study findings demonstrated an ORR of 25% and an OS of 8.2 months. Additionally, patients with DNA repair defects (DRD^+^ tumors) were found to have better clinical responses and longer immune response durations than DRD^−^ tumors (ORR, 40% vs. 0%; PFS HR, 0.31; OS HR, 0.41) [201]. Moreover, another study investigated the application of 3 mg/kg of ipilimumab and 1 mg/kg of nivolumab in patients who had experienced hormonal treatment failure but had not received chemotherapy and showed disease progression after chemotherapy, revealing an ORR of 25% and 10% and an mOS of 15.2 and 19 months, respectively. These observations are encouraging when compared to the efficacy of ICI monotherapies (ORR, 0–5%; mOS, 9.6–11.2 months). Furthermore, the above study assessed the potential biomarkers in patients with CRPC who may benefit from ICI combination therapy. The analysis determined that tumors with certain characteristics, such as high TMB, homologous recombination deficiencies (HRD) or DRD, high PD-L1 expression levels, MSI-H, and CDK12 alterations, could benefit from ICI combination therapy. Based on these findings, expanded studies are currently underway [202].

PSMA/CD3 BSABs have been shown to reduce prostate cancer tumor and PSA levels in the early stage, with a change of − 20.6% in PSA levels compared to baseline and PSA reductions of > 50% in 30–33.3% of the patients [199]. ICI combination therapies have also exhibited favorable anti-tumor activity and long-term survival benefits in patients with hormone therapy-resistant or progressive prostate cancer (ORR, 10–40%; mOS, 8.2–19 months) [201, 202]. Furthermore, no significant difference was detected in the response rates between the N1I3 and N3I1 combination therapies in patients with prostate cancer, as demonstrated by their comparable ORRs (both 25%; Fig. 5g, h and Supplementary Table S8). Moreover, patients with prostate cancer exhibiting high TMB, HRD or DRD, high tumor PD-L1 expression levels, MSI-H, and CDK12 changes experience relatively greater benefits from ICI combination therapies [202]. The incidence of AEs associated with PSMA/CD3 BSABs is also higher than that of ICI combination therapies (grade ≥ 3 TRAEs, 49–63% vs. 42.2–46%). The AEs in PSMA/CD3 BSABs mainly present as decreased lymphocytes and fever, while those in ICI combination therapies primarily comprise colitis/diarrhea, pneumonia, and elevated lipase levels (Fig. 5i and Supplementary Table S8).

### Melanoma

Uveal melanoma (UM), originating from melanocytes, is the most prevalent intraocular malignancy. Approximately 50% of patients with UM develop metastatic disease, resulting in a poor survival rate (mOS, 6–12 months) [203]. UM expresses lower TMB and antigenicity than cutaneous melanoma, leading to a poor response to ICI therapy [204, 205]. Consequently, patients with high-risk or advanced metastatic melanoma have an unfavorable prognosis. The mPFS for patients with advanced melanoma is 8 months, with a low 5-year OS rate of 10% [206]. Nevertheless, ipilimumab administration has been suggested to improve the survival outcome of patients with unresectable or metastatic melanoma (3-year OS rate ≥ 20%) [207].

#### Bispecific antibodies

Pmel17/gp100 has been revealed to have significant transcription levels in melanoma, with a notable difference in the expression levels between melanoma cells and normal melanocytes [208]. Tebentafusp (MCGP100) can selectively recognize gp100 presented by the human leukocyte antigen (HLA)-A*02:01 on the cell membrane through a specific T-cell receptor (TCR) [209]. Tebentafusp has been reported to activate CD8^+^ T cells in a dose-dependent manner as well as to potently redirect and activate effector and memory CD8^+^ and CD4^+^ cells. This BSAB also secretes various cytokines (such as tumor necrosis factor-α [TNF-α] and IL-2), promoting the antitumor response [210]. Furthermore, tebentafusp eliminates melanoma cells by facilitating the induction of dendritic cell cross-presentation of melanoma antigens, thereby achieving continuous killing of the tumor cells [211]. A phase I/II trial of tebentafusp (dose: 20 µg, D1C1; 30 µg, D8 C1; 68 µg, D15C1) in patients with metastatic UM yielded an ORR of 5% and an mOS of 16.8 months. Common TRAEs included skin damage (associated with targeting melanoma) and CRS (caused by T-cell activation). The trial findings also suggested that patients with early rash occurrences experienced greater benefits and a longer mOS of 22.5 months [212]. In a study by Nathan et al., tebentafusp treatment was compared with pembrolizumab, ipilimumab, or dacarbazine monotherapy (control group) in patients with metastatic UM, focusing on their differences in OS. The tebentafusp group demonstrated a 1-year OS rate of 73%, while the control group achieved a rate of 59%. Furthermore, the estimated mOS was 21.7 and 16.0 months in the tebentafusp and control groups, respectively. Additionally, the tebentafusp group exhibited a significantly better mPFS than the control group (6-month PFS rate, 31% vs. 19%; HR for disease progression or death, 0.73; 95% CI [confidence interval], 0.58–0.94; *P* = 0.01). All these findings suggest that tebentafusp treatment can improve overall survival in patients with metastatic UM [213].

#### ICI combination therapies

Preclinical models have demonstrated that combining ipilimumab with nivolumab or pembrolizumab can potentiate specific T-cell infiltration in B16 melanoma, thereby enhancing the tumor immune response [13]. A phase II trial conducted by Postow et al. enrolled 142 patients with previously untreated and unresectable metastatic melanoma to investigate the outcomes of the combined treatment of ipilimumab (3 mg/kg) with nivolumab (1 mg/kg). The trial results showed that the ICI combination therapy demonstrated superior efficacy to ipilimumab monotherapy (ORR, 61% vs. 11%), even among patients with BRAF V600E mutations (ORR, 52% vs. 10%). Moreover, the efficacy of the ICI combination therapy (ipilimumab plus nivolumab) was independent of tumor PD-L1 expression. However, the combination group experienced a higher incidence of grade ≥ 3 TRAEs than the ipilimumab monotherapy group (54% vs. 24%), consisting mainly of colitis and diarrhea AEs. Furthermore, the ICI combination therapy significantly reduced the risk of disease progression and patient mortality compared with the ipilimumab monotherapy (HR, 0.40) [214]. Similarly, other studies and subsequent survival analyses have further demonstrated that the ICI combination therapy (ipilimumab plus nivolumab) yields sustained survival benefits in comparison with nivolumab monotherapy (3-year OS rate, 58% vs. 52%; 4-year OS rate, 53%). Additionally, patients who discontinued their treatment prematurely due to TRAEs in ICI combination therapy (36.4%) were still able to achieve longer survival from the ICI combination therapy (4-year OS rate, 46%) [215–218]. A study investigating the combination of nivolumab (3 mg/kg) plus ipilimumab (1 mg/kg) for advanced melanoma has revealed that this treatment regimen can provide enhanced safety without compromising the patients’ survival benefits. Furthermore, a 3-year follow-up of this study found that the incidence of grade ≥ 3 AEs was comparatively low (33.9% [N3I1, 3 mg/kg nivolumab plus 1 mg/kg ipilimumab] vs. 48.3% [N1I3, 1 mg/kg nivolumab plus 3 mg/kg ipilimumab]) [219]. Blank et al. employed ipilimumab plus nivolumab as adjuvant therapy for resectable stage III melanoma, with 78% of the patients achieving pCR. Moreover, a follow-up analysis after 25.6 months showed no recurrence. Furthermore, incorporating ipilimumab and nivolumab administration before and after surgery recruited more tumor-infiltrating T cells. However, this regimen is associated with a high occurrence rate of treatment-related toxicities, suggesting the need to explore measures to reduce this toxicity [220]. In light of this issue, a phase III trial by Weber et al. demonstrated that patients with stage IIIB–D or stage IV melanoma who were administered adjuvant therapy using nivolumab (480 mg, q4w) showed comparable PFS when treated with the combination of nivolumab (240 mg, q2w) plus ipilimumab (1 mg/kg, q6w). However, nivolumab monotherapy exhibited lower treatment toxicity than this combination treatment (grade ≥ 3 TRAEs, 12.8% vs. 32.6%) [221].

The therapeutic combination of relatlimab (BMS-986,016, a LAG-3 inhibitor) and nivolumab (a PD-1 inhibitor) is also being investigated in patients with melanoma. A phase I/IIa study enrolled patients who experienced disease progression after receiving immunotherapy, including PD-1/PD-L1 inhibitor immunotherapy. The study highlighted that the combined blockade of the LAG-3 and PD-1 signaling pathways produced promising efficacy (ORR, 11%). Furthermore, an enhanced therapeutic efficacy (ORR, 17%) was associated with LAG-3 expression (≥ 1%), irrespective of PD-L1 expression. The researchers also reported that the safety profile of this combination regimen was similar to that of nivolumab monotherapy, with no additional treatment-related toxicities [222]. Subsequently, Tawbi et al. conducted a phase II/III trial recruiting patients with untreated advanced melanoma to undergo a combined treatment with relatlimab and nivolumab. The trial results indicated that the combination therapy had superior efficacy compared to nivolumab monotherapy (ORR, 43% vs. 33%), along with improved survival benefits (mPFS, 10.1 vs. 4.6 months; mOS, NR vs. 34 months; 24-months OS rate, 63.7% vs. 58.3%). Subgroup analyses further revealed that ICI combination therapies (nivolumab plus relatlimab) attained superior outcomes compared to nivolumab monotherapy, regardless of the BRAF mutation status, PD-L1 expression, and LAG-3 expression. Additionally, only 18.9% of the patients treated with ICI combination therapies experienced grade 3/4 TRAEs, primarily characterized by pruritus, fatigue, and dermatitis [223–225]. Based on the above experimental results, the treatment regimen of nivolumab plus relatlimab was approved in the United States on March 2022 for treating unresectable or metastatic melanoma in adults and children aged ≥ 12 years and weighing ≥ 40 kg [226]. An indirect cross-comparison study suggested that inhibiting the LAG-3/PD-1 and CTLA-4/PD-1 signaling pathways yields similar PFS. However, the blockade of the LAG-3/PD-1 signaling pathway may lead to earlier survival advantages and reduced TRAE incidence [227].

**Fig. 6:**
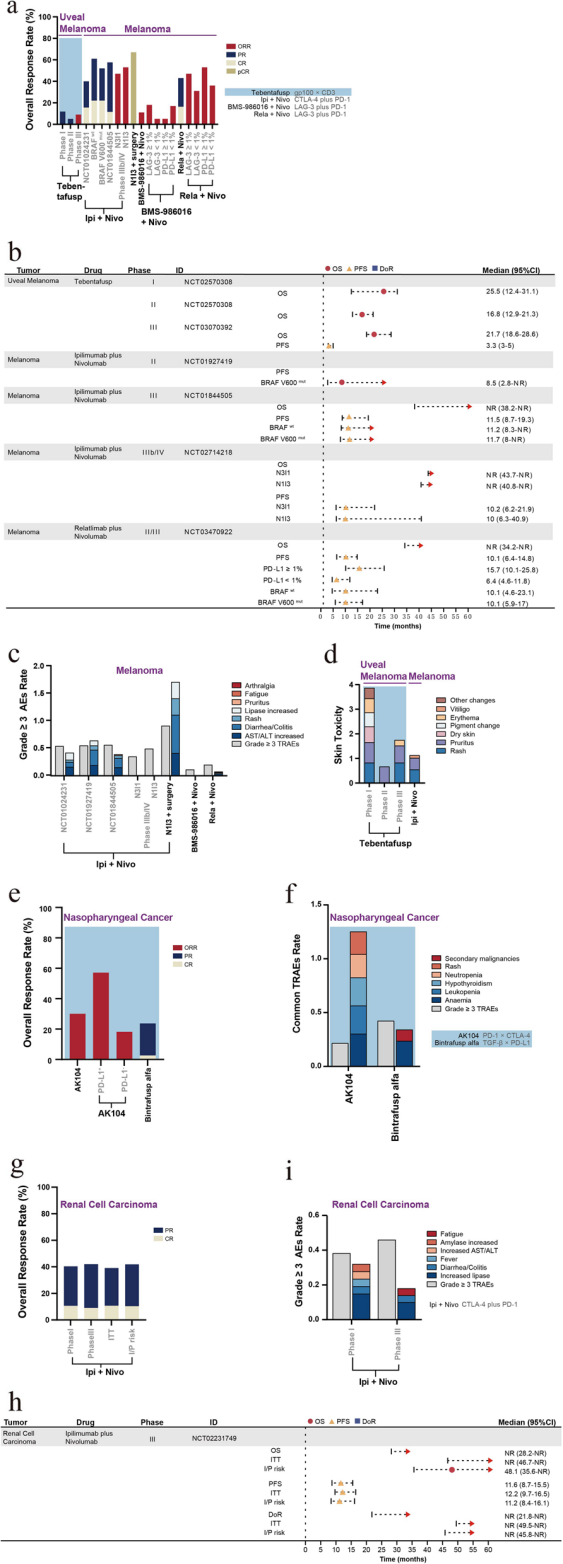
Efficacy, adverse events, and survival outcome of bispecific antibody treatment and ICI combination therapies in patients with Melanoma, nasopharyngeal cancer, and renal cell carcinoma. A histogram of antitumor activity (**a**), a forest plot of survival outcomes (**b**), incidence of grade ≥ 3 TRAEs, including major components (**c**) and skin toxicity (**d**) of BSABs and ICIs combination therapies in patients with melanoma. Rela, Relatlimab (anti- LAG-3). A histogram of antitumor activity (**e**) and common adverse effects components, including incidence of grade ≥ 3 TRAEs (**f**) of BSABs in patients with nasopharyngeal cancer. A histogram of antitumor activity (**g**), a forest plot of survival outcomes (**h**) and incidence of grade ≥ 3 TRAEs, including components (**i**) of ICIs combination therapies in patients with renal cell carcinoma. ITT, intent-to-treat patients; I/P risk, patients with intermediate/poor-risk disease. The ORR, defined as a sum of CR and PR. A bar with a value of 0 means not mentioned in the article

In summary, BSABs for melanoma are primarily employed in UMs characterized by low TMB and antigenicity. They provide superior survival benefits compared to ICI monotherapy (1-year OS rate, 73% vs. 59%; mOS, 21.7 vs. 16.0 months) [213]. Additionally, the combination of 1 mg/kg of nivolumab plus 3 mg/kg of ipilimumab has been approved for patients with unresectable or metastatic melanoma, conferring significant long-term survival benefits (ORR, 52–61%; 4-year OS rate, 53%), including in patients who discontinued their treatment due to AEs (4-year OS rate, 46%) [215–218]. Similarly, the blockade of the LAG-3/PD-1 signaling pathway elicits a certain degree of antitumor activity (ORR, 11–43%) [222–225], particularly in untreated patients (ORR, 43%). However, it provides a lower response rate than that achieved by blocking the PD-1/CTLA-4 signaling pathway (Fig. 6a, b and Supplementary Table S5). Although inhibiting the PD-1/LAG-3 signaling pathway does not result in a high response rate without the blocking of the PD-1/CTLA-4 signaling pathway (Fig. 6a, b and Supplementary Table S5), it significantly improves the treatment safety (Fig. 6c). In addition, the two combined approaches exhibited similar PFS (mPFS, 10-11.7 months for blockade of the PD-1/CTLA-4 signaling pathway and 6.4–15.7 months for blockade of the LAG-3/PD-1 signaling pathway). The major AEs associated with ICI combination therapies include elevated liver enzyme levels, colitis/diarrhea, and dermatological manifestations (Fig. 6c and Supplementary Table S5). These immune-related AEs can be managed via immunosuppressants or immunomodulators. Furthermore, BSABs can directly eliminate gp100-expressing melanocytes, causing more pronounced skin damage (mainly manifesting as rash and pruritus) than ICI combination therapies (Fig. 6d). In patients with resectable stage IIIB–D or stage IV melanoma, ICI combination therapies as neoadjuvant treatment are associated with increased AEs (grade ≥ 3 TRAEs, 32.6%; Fig. 6c and Supplementary Table S5) [220, 221].

### Nasopharyngeal cancer

Nasopharyngeal cancer (NPC) is highly prevalent in Southeast Asia and southern China. Currently, the main treatment options for NPCs are radiotherapy and chemotherapy. Although the 5-year OS rate exceeds 60% in patients with advanced NPC, approximately 30% experience recurrence following radiotherapy and chemotherapy, ultimately leading to a poor prognosis [228].

#### Bispecific antibodies

Epstein–Barr virus (EBV)-associated NPC exhibits upregulated PD-1/PD-L1 signaling pathway and dense penetration of TILs within the TME, thereby resulting in enhanced immune evasion. PD-1 inhibitors have shown clinical efficacy in patients with recurrent or metastatic NPC, with an ORR of approximately 20–30% [229]. Furthermore, PD-1 inhibitors stimulate IFN-β to promote NK cell activity via the mechanism of TNF-related apoptosis-inducing ligand [230]. Additionally, AK104 (a PD-1/CTLA-4 BSAB) treatment in patients with metastatic NPC who have failed second-line chemotherapy has demonstrated favorable antitumor activity (ORR, 30%; DCR, 70%), particularly in PD-L1-positive patients (ORR, 57.1%). AK104 also exhibited good safety, with only 21.7% of the patients experiencing grade ≥ 3 TRAEs [231].

Bintrafusp alfa (a PD-L1/TGF-β BSAB) has shown certain antitumor activity in patients with NPC who have failed platinum-based chemotherapy (ORR, 23.7%; 1-year OS rate, 57.5%). Moreover, patients who reported a decrease in EBV DNA levels at 4 weeks before the treatment had a higher ORR than those who did not experience such a decline (ORR, 40% vs. 6.3%). Additionally, the expression of PD-L1 and clearance rate of TGF-β in tissue and plasma, respectively, were not associated with NPC prognosis. Furthermore, 42.4% of the patients experienced grade ≥ 3 TRAEs, mainly consisting of anemia (23.7%) and secondary tumor occurrence (10.5%) [232].

A simple comparison of PD-1/CTLA-4 BSABs with bintrafusp alfa suggested that PD-1/CTLA-4 BSABs had increased efficacy in patients with recurrent or metastatic NPC who have failed second-line or above chemotherapy (ORR, 30% vs. 23.7%), particularly in patients with high PD-L1 expression (ORR, 57.1%). Furthermore, PD-1/CTLA-4 BSABs treatment was associated with a lower incidence of grade 3 AEs than bintrafusp alfa (30.4% vs. 42.4%; Fig. 6e, f and Supplementary Table S9).

### Renal cell carcinoma

Renal cell carcinoma (RCC) comprises approximately 2.4% of global cancer cases, wherein approximately 30% of these patients present with metastatic disease at diagnosis [233]. Studies have demonstrated that blocking the PD-1/PD-L1 signaling pathway confers survival benefits to patients with RCC (ORR, 25%; mPFS, 4.6 months; mOS, 25.0 months) [234].

#### ICI combination therapies

Hammers et al. conducted a phase I dose-finding trial in patients with advanced RCC and reported that the combination regimen of N3I1 led to lower toxicity than the N1I3 combination treatment (grade ≥ 3 TRAEs, 38.3% vs. 61.7%). The main AEs observed were increased lipase levels, colitis/diarrhea, and elevated liver enzyme concentration (Fig. 6i and Supplementary Table S10). Nevertheless, the N3I1 and N1I3 regimens showed similar survival benefits and a sustained therapeutic potential (ORR, 40.4% in both treatments; CR, 10.6% vs. 10%; 2-year OS rate, 67.3% vs. 69.6%, respectively; Fig. 6g, h and Supplementary Table S10) [235]. A phase III trial recruited patients with previously untreated advanced RCC to further verify the clinical efficacy of the N3I1 combination regimen. The study results also showed significantly better survival-risk benefits with the ICI combination therapy than with sunitinib monotherapy (ORR, 42% vs. 27%; 18-month OS rate, 75% vs. 60%), along with a lower incidence of AEs (grade ≥ 3 TRAEs, 46% vs. 63%). Additionally, these AEs were mainly composed of increased lipase levels, colitis/diarrhea, and fatigue (Fig. 6g, h, i and Supplementary Table S10) [236]. Subsequent analysis of the health-related quality of life (HRQoL) in patients with advanced RCC indicated that compared to sunitinib monotherapy, ICI combination therapies (nivolumab plus ipilimumab) improved the HRQoL and the Functional Assessment of Cancer Therapy Kidney Symptom Index-19 (FKSI-19) scores for disease-related symptoms, physical disease-related symptoms, and treatment side effects [237]. In a 4-year follow-up analysis of the previously mentioned phase III trial, ICI combination therapies (nivolumab plus ipilimumab) demonstrated more durable benefits than sunitinib in terms of OS and PFS in patients with intermediate- or poor-risk disease (mOS, 48.1 vs. 26.6 months; 4-year PFS rate, 32.7% vs. 12.3%) [238].

### Malignant pleural mesothelioma

Malignant pleural mesothelioma (MPM) is an aggressive tumor originating from the mesothelial cells of the pleura. This tumor is characterized by a short OS period, particularly in the unresectable stage. In terms of treatment outcome, approximately 10–20% of patients exhibit a response to platinum-based chemotherapy, with an mOS of 5.6–10.9 months [239].

#### ICI combination therapies

A study reported that nivolumab monotherapy and nivolumab in combination with ipilimumab have significant efficacy in patients with MPM (mOS, 11.9 and 15.9 months; 1-year OS rate, 49.2% and 58.1%, respectively) [240]. This finding indicates that immunotherapy may offer a novel therapeutic option for MPM. Another study enrolled patients with MPM who had progressed after platinum-based chemotherapy and treated them with nivolumab (240 mg, q2w) and ipilimumab (1 mg/kg, q6w for up to 4 cycles). The research revealed an ORR of 38%, a 12-month OS rate of 64%, and a predicted mOS of 12.7 months. Furthermore, the results indicated that 73% of PD-L1-positive patients experienced clinical benefits, significantly higher than the proportion of PD-L1-negative patients (32%) exhibiting treatment benefits. Consequently, the researchers suggest that PD-L1 may be a potential biomarker for the combined treatment of nivolumab and ipilimumab [241]. In a phase III study, ICI combination therapies (nivolumab plus ipilimumab) were compared with first-line chemotherapy in terms of OS changes in patients with MPM. Treatment-naïve patients in the combination therapy group received 3 mg/kg of nivolumab plus 1 mg/kg of ipilimumab for up to 2 years. The study results demonstrated similar ORRs between the combination treatment and chemotherapy groups (40% vs. 43%). However, ICI combination therapy showed significant OS benefits compared to chemotherapy (mOS, 18.1 vs. 14.1 months; 2-year OS rate, 41% vs. 27%), particularly in non-epithelioid MPM subtypes with a higher degree of malignancy (mOS, 18.1 vs. 8.8 months) [242]. A 3-year follow-up report indicated that ICI combination therapies (nivolumab plus ipilimumab) provided long-term survival benefits and sustained responses compared to chemotherapy (3-year OS rate, 23% vs. 15%; Supplementary Table S11). Furthermore, patients who discontinued treatment due to AEs also exhibited durable responses (mOS, 25.4 months), with 34% continuing to show a response at 3 years [243].

**Fig. 7:**
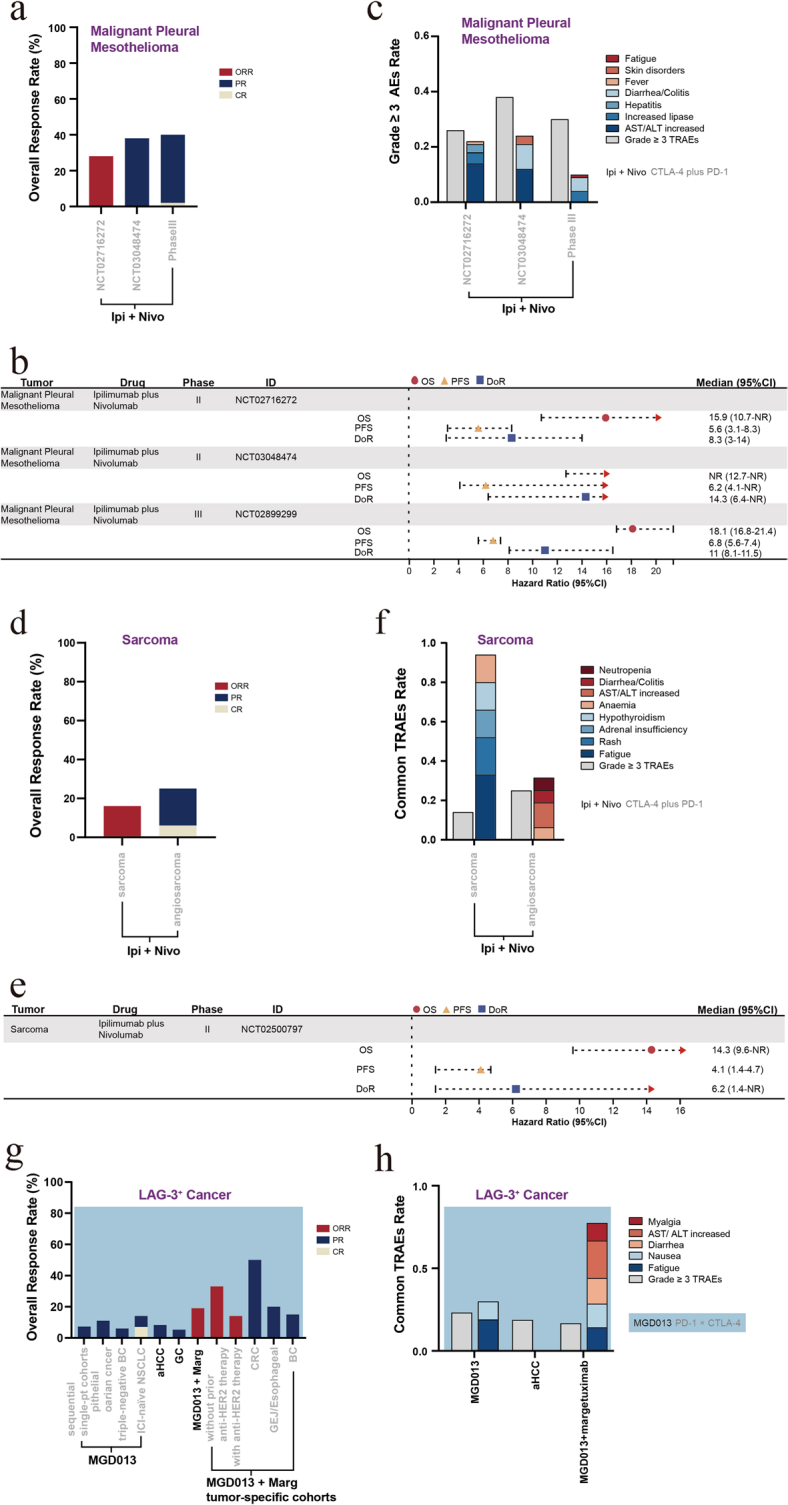
Efficacy, adverse events, and survival outcome of bispecific antibody treatment and ICI combination therapies in patients with Malignant pleural mesothelioma, sarcoma, and LAG-3-positive cancer. A histogram of antitumor activity (**a**), a forest plot of survival outcomes (**b**) and incidence of grade ≥ 3 TRAEs, including major adverse effects components (**c**) of ICIs combination therapies in patients with malignant pleural mesothelioma. A histogram of antitumor activity (**d**), a forest plot of survival outcomes (**e**) and common all-grade TRAEs components, including incidence of grade ≥ 3 TRAEs (**f**) of ICIs combination therapies in patients with sarcoma. A histogram of antitumor activity (**g**) and common TRAEs components, including incidence of grade ≥ 3 TRAEs (**h**) of BSABs in patients with LAG-3-positive cancer. MGD013, Tebotelimab; Marg, Margetuximab (anti-HER2); BC, breast cancer; NSCLC, non-small cell lung cancer; aHCC, advanced hepatocellular carcinoma; GC, gastric carcinoma; CRC, colorectal cancer. The ORR, defined as a sum of CR and PR. A bar with a value of 0 means not mentioned in the article

The ICI combination therapy consisting of 3 mg/kg of nivolumab and 1 mg/kg of ipilimumab has demonstrated substantial antitumor activity (ORR, 28–40%) in patients with MPM, resulting in prolonged survival (mOS, 18.1 months; 3-year OS rate, 23%; Fig. 7a, b and Supplementary Table S11) [241, 243]. The safety of ICI combination therapies in MPM is consistent with that observed in other tumors (grade ≥ 3 TRAEs, 26–30%). The most commonly reported AEs include elevated hepatic enzyme levels, colitis/diarrhea, and dermatological manifestations, although these side effects are generally manageable (Fig. 7c and Supplementary Table S11). In October 2020, the United States approved the first-line use of nivolumab in combination with ipilimumab for unresectable MPM, including for the non-epithelioid and epithelioid subtypes.

### Sarcoma

Sarcoma is a rare, heterogeneous malignant tumor derived from the mesenchymal tissue. The first-line therapy for advanced or metastatic soft tissue sarcoma is primarily doxorubicin, with one trial comparing doxorubicin or gemcitabine with docetaxel, which were found to be comparable in terms of efficacy (6-month PFS rate 46.3% vs. 46.4% for gemcitabine with docetaxel) [244]. Additionally, metastatic vascular sarcoma is associated with high mortality rates and poses therapeutic challenges. Although 18–89% of patients with metastatic vascular sarcoma show responsiveness to paclitaxel, these responses are not durable (mPFS, 4–9.5 months; 5-year OS rate, 30–40%) [245].

#### ICI combination therapies

The therapeutic options for metastatic sarcoma are limited, but ICIs have shown potential applicability. A phase II study investigated the efficacy of nivolumab monotherapy or nivolumab in combination with ipilimumab for patients with unresectable metastatic sarcoma. The results indicated that the ICI combination therapy had better anti-tumor activity in various sarcoma types (including vascular sarcoma) than nivolumab monotherapy (ORR, 16% vs. 5%; mOS, 14.3 vs. 10.7 months; 6-month CBR, 12% vs. 2%; 12-month CBR, 12% vs. 10%; Fig. 7d, e and Supplementary Table S12). However, 14% of the patients who underwent the ICI combination therapy experienced grade 3/4 TRAEs, predominantly anemia and hypotension (Fig. 7f and Supplementary Table S12), which was higher than the proportion of patients with grade ≥ 3 TRAEs who received monotherapy (7%) [246]. In a phase II trial of rare cancers, the use of nivolumab (240 mg, q2w) plus ipilimumab treatment for metastatic or unresectable vascular sarcoma was examined. The trial revealed an ORR of 25% (4/16 patients; CR, one patient), with a positive response observed in 60% (3/5) of patients with primary vascular sarcomas of the scalp or face (Fig. 7d, e and Supplementary Table S12). Approximately 12.5% of patients experienced grade ≥ 3 TRAEs, primarily comprising elevated liver enzyme levels and diarrhea (Fig. 7f and Supplementary Table S12). The researchers concluded that the combination therapy of nivolumab with ipilimumab in angiosarcomas requires further exploration [247].

### LAG-3 high-expression cancer

LAG-3 is expressed in activated T and NK cells. Similar to PD-1 expression, LAG-3 is preferentially expressed on Tregs within the TME, manifesting as T-cell exhaustion [15]. Moreover, LAG-3 exhibits high expression in various malignancies, including DLBCL, cervical cancer, head and neck squamous cell carcinoma, BC, gastric cancer, and anal cancer. In the analysis of NSCLC specimens, 92.3% of the patients demonstrated positive immunohistochemistry for LAG-3 and PD-1 dual expression, with 60% of the TILs co-expressing LAG-3 and PD-1 [248]. Thus, inhibiting the PD-1 and LAG-3 signaling pathways may help reverse T-cell immunosuppression, thereby enhancing the anti-tumor immune response.

#### Bispecific antibodies

Tebotelimab (MGD013) is a PD-1/LAG-3 BSAB possessing superior T-cell activating capacity compared to blocking the PD-1 or LAG-3 signaling pathways individually [249]. A phase I trial indicated that tebotelimab blocks both the PD-1 and LAG-3 signaling pathways, displaying preliminary anti-tumor activity, particularly in ovarian epithelial cancer (PR, 11.1%), triple-negative BC (PR, 6.5%), and ICI-naïve NSCLC (ORR, 14.2%; CR, 7.1%). Furthermore, the treatment exhibited favorable safety (grade ≥ 3 TRAEs, 22%), with fatigue and nausea being the primary AEs. Researchers have observed that tumors exhibit elevated expression level of LAG-3, while patients displaying increased expression in genes related to IFN-γ regulation often show more favorable clinical responses [174, 250]. Ren’s trial in advanced hepatocellular carcinoma demonstrated a similar safety profile for tebotelimab (grade ≥ 3 TRAEs, 18.8%) with some antitumor activity observed in both ICI-naïve and ICI-experienced patients (ORR, 13.3% and 3.3%; DCR, 50% and 46.7% ICI -naïve and ICI-experienced, respectively), primarily manifested as disease stabilization [251]. Similarly, tebotelimab combined with niraparib, a poly (ADP-ribose) polymerase inhibitor, exhibited limited anti-tumor activity in patients with metastatic advanced gastric cancer (ORR, 5.3%; DCR 52.6%) [252]. However, further investigations regarding these two trials have been terminated. Catenacci et al. highlighted that combining margetuximab (a HER2 Mab) with pembrolizumab (a PD-1 inhibitor) enhances T-cell anti-tumor responses in HER2^+^ tumors [253]. Studies have indicated that margetuximab application leads to an upregulation of LAG-3/PD-L1 expression on immune cells, along with enhanced margetuximab-induced tumor lysis in the presence of tebotelimab [254]. Consequently, some researchers suggest that the dual blockade of the PD-1 and LAG-3 signaling pathways can improve both the innate and adaptive immune responses against HER2-overexpressing tumors, thereby enhancing the efficacy of anti-HER2 therapies. Early results of the combined treatment of tebotelimab and margetuximab in advanced HER2^+^ tumors, including BC, bile duct cancer, esophageal adenocarcinoma, microsatellite-stable colon cancer, and GEJ cancer, have shown encouraging anti-tumor activity (ORR, 19%) and acceptable safety (grade ≥ 3 TRAEs, 16.7%). The AEs mainly manifest elevated liver enzyme levels, diarrhea, nausea, and myalgia etc. (Fig. 7g, h and Supplementary Table S13) [254].

## Discussion

Tumoral pathologies manifest a significant degree of heterogeneity, primarily attributed to the intricate pathogenic factors and the spectrum of genetic aberrations, which underscoring the urgency for personalized and precise cancer therapies. In recent years, the domain of tumor immunotherapy has witnessed accelerated advancements and become the standard treatment for various tumors. Both effector cell redirection and immunomodulatory agents are instrumental in the activation tumor-targeting immune cells, making them prevalent in clinical practice. Bispecific T-cell engagers (BiTEs), a primary type of T-cell redirecting BSABs, have been developed for various malignancies. These include CD3/CD19 and CD3/CD20 BSABs for B-cell lymphoma/leukemia; CD3/BCMA, CD3/FcRH5, and CD3/GPRC5D BSABs for MM; CD3/EpCAM BSABs for peritoneal carcinoma and malignant ascites, CD3/gp100 BSABs for UV; CD3/HER2 BSABs for HER2-positive tumors; and CD3/PSMA BSABs for prostate cancer, etc. (Fig. 1a).

BiTEs primarily target the TCR-CD3 complex and tumor-specific antigens typically overexpressed in tumors but expressed minimally or not at all in normal tissues. BiTEs steer cytotoxic T cells toward malignant tumors, promoting immunological synapse formation and cytokine release, resulting in tumor lysis and immune activation. Moreover, T-cell activation via this mechanism is independent of the tumor-specific antigens, facilitating the induction of this process even at low antibody doses or minimal tumor antigen expression [255, 256]. Furthermore, in contrast to monoclonal antibodies (Mabs), BSABs possess enhanced specificity, allowing the precise targeting of the tumor with minimized off-target toxicity. Unlike systemic immune modulation, the antitumor response elicited by BSABs is predominantly confined to the tumor vicinity, thereby curtails the occurrence of of AEs [5, 6]. In particular, BSABs can concurrently engage two distinct regulatory pathways simultaneously, thereby amplifying their antitumor effects. For example, bintrafusp alfa, a TGF-β/PD-L1 BSAB can recruit NK and CD8^+^ T cells to initiate a dual attack on tumors. Additionally, bintrafusp alfa facilitates the upregulation of major histocompatibility complex class I (MHC-I), MHC-II, and PD-L1 expression, promoting the stability of immunological synapses and triggering downstream signaling pathways [188, 249, 257]. These principles and discoveries pave the way of enhancing the clinical efficacy and safety of BSABs. Nonetheless, BiTEs have been mainly applied in hematologic malignancies, wherein they exhibit favorable clinical effectiveness. Compared to hematological malignancies, BiTEs have shown reduced response rates in solid tumors. This outcome discrepancy may be attributed to the physical barriers in the TME of solid tumors, oftentime called “cold” tumors that are characterized by a lack immune cell infiltration [258].

The primary focus of research on ICI combination therapies currently involves the blockade of the CTLA-4 and PD-1/PD-L1 signaling pathways. Several studies have found that CTLA-4 blockade may lead to the upregulation of PD-1 expression, whereas simultaneously inhibiting PD-1 and CTLA-4 can impede T-cell exhaustion [9]. PD-1 and CTLA-4 inhibitors can concurrently act on the same T cells, enhancing T-cell activation via the AKT/PI3K signaling pathway [259]. These inhibitors can also separately stimulate T cells in the TME and lymph nodes/tissues, promoting T-cell activation [10, 260]. Multiple studies have demonstrated that ICI combination therapies yield higher response rates than single ICIs [9]. Notably, the drug responses vary across different tumors. In HL [55], NSCLC [99], melanoma [219], RCC [236], colorectal cancer [157–159], MPM [241, 242], and sarcoma [246], the treatment regimen of nivolumab (3 mg/kg) in combination with ipilimumab (1 mg/kg) has shown promising outcome. Conversely, patients with gastric, esophageal, or GEJ adenocarcinomas [137] and HCCs [147, 148] benefit from a nivolumab (1 mg/kg) and ipilimumab (3 mg/kg) regimen, showing improved efficacy and survival. Other ICI combination therapies are being explored as well.

### Applications

BSABs and ICI combination therapies have demonstrated immense therapeutic potential in the realm of tumor immunotherapy. Specifically, BSABs offer therapeutic benefits for thoese patients who have failed Mab therapy or undergone tumor progression post CAR-T therapy. However, due to the limitations in developing BSAB targets, the overall clinical indications and research progress of BSABs are slower compared to ICIs. To date, ten BSABs have been approved for tumor treatment. These include blinatumomab (a CD19/CD3 BSAB) for treating B-ALL, epcoritamab (a CD20/CD3 BSAB) for R/R DLBCL, mosunetuzumab (a CD20/CD3 BSAB) for FL, teclistamab (a BCMA/CD3 BSAB) for MM, amivantamab (an EGFR/cMET BSAB) for NSCLC with EGFR ex20ins mutations, and cadonilimab (AK104, PD-1/CTLA-4 BSABs) for recurrent/metastatic cervical cancer. On the other hand, ICI combination therapies have been approved for the treatment of metastatic melanoma, advanced or metastatic RCC, MSI-H colorectal cancer that has progressed after chemotherapy, advanced HCC, driver-gene-negative NSCLC with PD-L1 expression ≥ 1%, and unresectable MPM.

Our study further underscores the substantial therapeutic potential of GPRC5D/CD3 BSABs in treating MM. Moreover, for patients with HER2-overexpressing G/GEJ cancer who have not responded to first-line therapies, as well as those with advanced BTC, KN026 and zanidatamab (BSABs targeting HER2 domains ECD2 and ECD4) present promising therapeutic options. These therapies have demonstrated an ORR of 56% and an mOS of 16.3 months in G/GEJ cancer, and an ORR of 47% in BTC) (Supplementary Table S4) [129]. Additionally, patients with advanced G/GEJ cancer may benefit from the regimen combining AK104 (a PD-1/CTLA-4 BSAB) with chemotherapy, as evidenced by the encouraging results (ORR, 65.9%; mOS, 17.41 months) [133]. Zenocutuzumab (a HER2/HER3 BSAB) is an orphan drug for NRG1^+^ pancreatic ductal adenocarcinoma (ORR, 39%) [96]. Patients with metastatic BC, previously treated with anti-HER2 therapies and exhibiting HER2 amplification and NRG1^+^, may benefit from zenocutuzumab treatment (DCR, 77%) [173]. Tebentafusp (a gp100/CD3 BSAB) confers survival benefits to patients with metastatic melanoma, leading to extended OS (mOS, 21.7 months) [213]. Patients with prostate cancer progression after hormone therapy or exhibiting hormone resistance or those having prostate cancer with high TMB or HRD/DRD, or MSI-H or accompanied with CDK12 changes or AR-V7^+^ characteristics can receive ICI combination therapies (ORR, 10–40%; mOS, 8.2–19 months) [202]. PD-L1 overexpression in patients with NPC who have failed second-line or above chemotherapy allows for the application of AK104 (a PD-1/CTLA-4 BSAB), yielding an ORR of 57.1% [231]. ICI combination therapies also demonstrate efficacy in treating sarcomas considered challenging to treat (ORR, 16–25%; mOS, 14.3 months) [246, 247].

LAG-3 and PD-1 are continuously co-expressed on TILs, and combined blockade of LAG-3 and PD-1 pathways has shown promise in improving the inhibitory tumor microenvironment [15, 16]. Several PD-1/LAG-3 BSABs have demonstrated greater T-cell activity and IFN-γ production compared to strategies involving either Mab or combination of two Mabs. This approach is advantageous for restoring the exhausted T-cell function within the tumor microenvironment while limiting the occurrence of severe systemic toxicities. The PD-1 × LAG-3 ICI combination therapies have been shown improve PFS compared to PD-1 Mabs in melanoma [223]. Additionally, the therapeutic response of PD-1/LAG-3 BSABs is generally associated with LAG-3 expression and the expression of genes involved in IFN-γ regulation, rather than the expression of PD-L1. These studies underscore the potential of anti-LAG-3 therapy to enhance the outcomes of solid tumors with high LAG-3 expression, particularly those exhibit suboptimal response or resistance to anti-PD-1 therapy, such as ovarian epithelial cancer and triple-negative breast cancer [250, 261, 262]. The study demonstrated an augmentation in the expression of LAG-3 and PD-1 on TILs and malignant B cells in patients with relapsed DLBCL following CAR-T therapy. Previous research investigating the combination of CD19 CAR-T and PD-1 Mab did not show improvement in therapeutic efficacy [263]. However, patients responding to tebotelimab demonstrated elevated levels of LAG-3 [250]. This observation may offer valuable insights for future combination of anti-LAG-3 and CAR-T therapy. Additionally, the combination of tebotelimab and margetuximab (a HER2 Mab) has shown enhanced therapeutic efficacy in refractory HER2^+^ tumors, even in patients previously treated with anti-HER2 therapy (*n* = 7/21; ORR, 33%). This includes breast cancer (ORR, 13.3%) and gastric adenocarcinoma (ORR, 22.2%) [250]. This approach provides new treatment strategies for patients who are resistant to HER2 Mabs. Researchers posit that PD-1/LAG-3 BSABs offer promising immunotherapeutic opportunities for patients resistant to PD-1/PD-L1 Mabs. However, further investigation is warranted to elucidate the identification of such patient populations. Consequently, additional exploration is necessary to deepen the understanding of PD-1/LAG-3 BSABs.

### Limitations

BSABs and ICI therapies have demonstrated high response rates in both preclinical and clinical trials. However, these response rates are not universal, as a subset of patients experiencing recurrence. Resistance to T-cell mediated immunotherapy is multifactorial, primarily involving intrinsic tumor properties-such as the status of tumor antigens and tumor heterogeneity, along with T-cell functionality and the dynamics of the tumor microenvironment.

In a trial involving R/R adult B-ALL treated with Blinatumomab, approximately 30% of the relapsed population exhibited loss of CD19 antigen [264, 265]. Furthermore, following the Mosunetuzumab administration, 27% (7/26 pts) of relapsed patients experienced a loss of CD20 expression in tumor cells [266]. The attenuation of tumor antigens may impede the targeted binding, thereby diminishing the therapeutic efficacy. Truger’s research, employing whole genome sequencing on penta-refractory MM patients who treated with AMG420 (a BCMA/CD3 BSAB), suggests that a homozygous deletion of the BCMA gene on chromosome 16p underlies the failure of BCMA-targeted T-cell immunotherapy. Recent studies suggest that that biallelic events (del/del and del/mut) serve as mechanisms for antigen escape subsequent to targeted tumor surface antigen therapy. The pressure from targeted therapy can potentially lead to irreversible antigen loss or its mutation. Therefore, it is posited that the early implementation of immunotherapeutic strategies might be beneficial in the patients with higher antigen mutation burden. Additionally, newly diagnosed MM has a lower frequency of deletions and mutations in genes encoding immunotargets compared to R/R MM [267, 268]. Liu found by CRISPR that the loss of CD123 core fucosylation impedes the interaction between CD123 and CD3, thereby diminishing the anti-tumor effect of CD123 x CD3 BSAB [269]. Additionally, Broeske et al’s investigation into biomarkers pertinent to Glofitamab treatment has elucidate a significant overexpression of MYC and a concurrent downregulation of TP53 in non-CR patients, particularly those with disease progression [44, 270]. Aberrant TP53 signaling can contribute to T-cell dysfunction in the TME of DLBCL through upregulation of PD-L1 and loss of MHC-II gene expression [271]. These findings suggest that a multi-antigen targeted antibodies or combination therapies may offer enhanced therapeutic benefits.

Prolonged antigen exposure or persistent receptor signaling have been implicated in the induction of T-cell exhaustion, thereby affecting T-cell based immunotherapy. T-cell exhaustion primarily manifests as the co-expression of inhibitory checkpoint molecules (such as PD-1/PD-L1) on T cells. This is accompanied by a progressive impairment of T-cell functions, evidenced by diminished cytokine production, reduced proliferative capacity, and attenuated cytotoxic activity, which undermine the efficacy of immunotherapy, highlighting the need for interventions that can reverse or mitigate T-cell exhaustion. Moreover, the TME harbors immune suppressive cells, immunosuppressive molecules, and extracellular matrix can potentially impact the infiltration of T cells recruited by BiTEs into the tumor [60, 73]. In preclinical investigations of Talquetamab, differential composition of the bone marrow microenvironment, encompassing the frequencies of effector cells (T cells) and immune suppressive cells (Tregs), have been found to influenced the antitumor activity of Talquetamab. Subsequent baseline-associated analyses conducted on study patients consistently showed that non-responsive individuals exhibit high expression of PD-1/TIM-3 and PD-1/CD38 on T cells in both peripheral blood and bone marrow [66, 272].

Multiple studies on Acute Myeloid Leukemia (AML) have observed significant expression of PD-L1 on CD34^+^ blasts, CD4^+^/8^+^ T cells, and Tregs, particularly in bone marrow samples from R/R patients [273]. Furthermore, researchers suggest a potential link between PD-L1 expression and cytokine release during targeted therapy. In resistant tumor cells to HER2/CD3 BSABs, a deficiency in the IFN-γ pathway has been identified to suppress T cell cytotoxicity-related genes [269]. Similarly, in preclinical investigations of CD20/CD3 BSABs, disrupting the IFNγ-CXCL10 axis results in the elimination of peripheral T cell recruitment [274].

ICI combination therapies aim to overcome the issue of low response rates of tumors to PD-1/PD-L1 inhibitors by targeting various immune regulatory pathways within the TME. However, they also carry an elevated risk of AEs. Researchers have reported an approximately threefold increase in toxicity incidence across all severity grades after the introduction of CTLA-4 inhibitors. These toxicities are characterized not only by an early onset but also prolong the duration of high-grade (grade 3/4) events, particularly with an increased risk of gastrointestinal toxicity incidence [275], and a heightened potential of inducing autoimmunity [276, 277].

### Future and challenges

Research into the resistance mechanisms of immunotherapy of BSABs is still limited. Researchers posit that the development of multi-antigen targeted antibodies, in combination with anti-PD-1/PD-L1 antibodies, as well as the targeting of co-stimulatory/inhibitory receptors, may further enhance the efficacy of anti-tumor therapy. Nora et al. discovered that treatment-free intervals could interrupt the sustained stimulation of T cells by antibodies, thereby amplifying T cell functionality and inducing transcriptional reprogramming [278]. Targeting the PD-1/PD-L1 signaling pathway or exploring the targeting of co-stimulatory or co-inhibitory receptors, has been shown to mitigate T-cell exhaustion [186, 279]. Studies have demonstrated that the blockade of the PD-1/PD-L1 pathway facilitates the lysis of AML cells by CD33/CD3 BSABs [280]. Preclinical studies emphasizing the inhibition of PD-1/PD-L1 in solid tumors underscore the potential to alleviate T-cell suppression within TME, thereby promoting the anti-tumor efficacy of BSABs [281]. Targeted immunotherapy for tumors selectively activates fewer tumor-specific T-cells, inducing sustained anti-tumor responses by immune memory cells. BSABs targeting CD40 and 4-1BB are currently under investigation, as they are believed to enhance T-cell activation [2, 282, 283]. Furthermore, demanding the search of BSABs targeting tumor immunomodulators with high affinity or high expression of tumor antigens is recommended. Moreover, the exploration of high affinity targets and additional forms of BSABs are pivotal factors in enhancing both the efficacy and safety of therapeutic therapy [2]. Researchers aim to address the treatment-related toxicity while amplifying therapeutic effects by appropriately adjusting the dosage ratio of ICI. Numerous clinical trials have employed a regimen of CTLA-4 antibodies administered once every 3 or 6 weeks for total 4 doses, alongside a continuous administration of PD-1/PD-L1 antibodies. Further studies are still essential to determine dosage regimens, and administration sequences that exhibit both efficacy and safety across various tumor types. The dynamic shifts in the TME of cancer patients during treatment present pose a significant challenge to maintain the efficacy of BSABs and ICI combination therapies. With an increasing array of targeted drugs and drug combinations, the selection of optimal treatment regimens becomes imperative. There is an urgent need for predictive biomarker exploration, enabling the targeting of individual immune characteristics. In conclusion, while the implementation of immunotherapy poses a significant challenge, we remain optimistic and anticipate the continued studies of BSABs will deepen the understanding of ICI combination therapies in the context of tumor immunotherapy. The progression of the knowledge will enhance the clinical efficacy and safety of these treatment strategies, and eventually benefits a broader spectrum of cancer patients.

### Supplementary Information

**Supplementary Material 1.**

## Data Availability

No datasets were generated or analysed during the current study.

## Abbreviations

BSABs: Bispecific antibodies
ICIs: Immune checkpoint inhibitors
PD-1: Programmed cell death protein-1
ICI combination therapies: ICI-based combination therapies
NK cells: Natural killer cells
TME: Tumor microenvironment
CTLA-4: Cytotoxic T-lymphocyte-associated antigen 4
LAG-3: Lymphocyte activation gene-3
Tregs: Regulatory T cells
TILs: Tumor-infiltrating lymphocytes
IL-2: Interleukin-2
NSCLC: Non-small cell lung cancer
Ig: Immunoglobulin
R/R: Relapsed or refractory
B-ALL: B-cell acute lymphoblastic leukemia
CR: Complete remission
mOS: Median overall survival
HSCT: Hematopoietic stem cell transplantation
ALL: Acute lymphoblastic leukemia
NHL: Non-Hodgkin's lymphoma
DLBCL: Diffuse large B-cell lymphoma
FL: Follicular lymphoma
PFS: Progression-free survival
Mabs: Monoclonal antibodies
HL: Hodgkin's lymphoma
mPFS: Median PFS
AE: Adverse events
CRS: Cytokine release syndrome
MTD: Maximum tolerated dose
ORR: Overall response rate
B-NHL: B-cell NHL
CMR: Complete metabolic response
mDoR: Median duration of response
D1C1: Day 1 of cycle 1
HRS: Reed–Sternberg
ADCC: Antibody-dependent cellular cytotoxicity
BV: Brentuximab vedotin
ASCT: Autologous stem cell transplantation
MM: Multiple myeloma
PCs: Plasma cells
BM: Bone marrow
IMiDs: Immunomodulatory drugs
PI: Proteasome inhibitors
BCMA: B-cell maturation antigen
BAFF: B cell-activating factor
FcRH5: Fc receptor-homolog 5
GPRC5D: G protein-coupled receptor family C group 5 member D
RP2D: Recommended phase II dose
VGPR: Very good partial response
EGFR: Epidermal growth factor receptor
ex20ins: Exon 20 insertion
TKIs: Tyrosine kinase inhibitors
SCLC: Small-cell lung cancer
IFN-γ: Interferon-γ
qw: Once per week
q2w: Once every 2 weeks
TGF-β: Transforming growth factor β
EMT: Epithelial-mesenchymal transition
TRAEs: Treatment-related AEs
DCR: Disease control rate
VEGFs: Vascular endothelial growth factors
CIK: Cytokine-induced killer
NRG1: Neuregulin-1
HER3: Human epidermal growth factor receptor 3
TMB: Tumor mutation burden
mut/Mb: Mutations per megabase
MPR: Major pathologic response
pCR: Pathologic complete remission
EpCAM: Epithelial cell adhesion molecule
HR: Hazard ratio
G/GEJ: Gastric/gastroesophageal junction
TGF-βRII: TGF-β receptor II
CEA: Carcinoembryonic antigen
ECD2: Extracellular domain 2
ESCC: Sophageal squamous cell carcinoma
MSI-H: Microsatellite instability-high
dMMR: Deficient mismatch repair
BTC: Biliary tract cancer
HCC: Hepatocellular carcinoma
mCRC: Metastatic colorectal cancer
pMMR: Proficient mismatch repair
MSS: Microsatellite stable
MGMT: O6-methylguanine-DNA methyltransferase
BC: Breast cancer
CBR: Clinical benefit rate
ER: Estrogen receptor
ET: Endocrine therapy
CDK4/6i: Cyclin-dependent kinase 4 and 6 inhibitor
SD: Stable disease
HPV: Human papillomavirus
CPS: Combined positive score
CRPC: Castration-resistant prostate cancer
AR-V7: Androgen receptor variant 7
PSMA: Prostate-specific membrane antigen
mCRPC: Metastatic CRPC
IV: Intravenous infusion
PSA: Prostate-specific antigen
SC: Subcutaneous
DRD: DNA repair defects
HRD: Homologous recombination deficiencies
UM: Uveal melanoma
HLA: Human leukocyte antigen
TCR: T-cell receptor
TNF-α: Tumor necrosis factor-α
N1I3: 1 Mg/kg nivolumab plus 3 mg/kg ipilimumab
NPC: Nasopharyngeal cancer
EBV: Epstein–Barr virus
RCC: Renal cell carcinoma
HRQoL: Health-related quality of life
FKSI-19: Functional assessment of cancer therapy kidney symptom index-19
MPM: Malignant pleural mesothelioma
BiTEs: Bispecific T-cell engagers
MHC-I: Major histocompatibility complex class I
