# Supplementary material for: Efficacy and safety of bispecific antibodies vs. immune checkpoint blockade combination therapy in cancer: a real-world comparison
Source: Mol Cancer. 2024 Apr 16;23:77. doi: 10.1186/s12943-024-01956-6 (PMC11020943; doi:10.1186/s12943-024-01956-6)
Supplement: Supplementary file 1 — Supplementary Material 1. [file 12943_2024_1956_MOESM1_ESM.docx]

**Table S1** Clinical trials of B-cell Lymphoma/Leukaemia

| Tumor | Drug | Target | Phase (ID) | Usage | Outcomes | Adverse Events |
| --- | --- | --- | --- | --- | --- | --- |
| Philadelphia-chromosome-negative, primary refractory or relapsed leukaemia | Blinatumomab | CD3 × CD19 | Phase II (NCT01466179) | 9 μg/day d1-7 and 28 μg/day w2-4 every 6 weeks (up to five cycles);  continuous intravenous infusion | CR 33%  allogeneic HSCT after CR 44%  mOS 6.1 mo (95% CI, 4∙2-7∙5)  mRFS 5.9 mo (95% CI, 4∙8–8∙3) | grade ≥ 3 AEs: 82%, febrile neutropenia 25%; neutropenia 16%; anaemia 14%; CRS 2%; neurologic events 13%  treatment-related deaths 3 pts |
| Philadelphia-chromosome-negative, primary refractory or relapsed leukaemia | Blinatumomab | CD3 × CD19 | Phase III  (NCT02013167) | 9 μg/day d1-7 and 28 μg/day w2-4 every 6 weeks (up to five cycles);  continuous intravenous infusion | CR 34%  mOS 7.7 mo (95% CI, 5.6 -9.6)  mPFS 7.3 mo (95% CI, 5.8-9.9) | grade ≥ 3 AEs: 86.5%, neutropenia 37.8%; infection 34.1%; elevated liver enzyme 12.7%; CRS 4.9%; neurologic events 9.4% |
| relapsed/refractory NHL | Blinatumomab | CD3 × CD19 | Phase I  (NCT00274742) | 60 µg/m^2^ (MTD), continuous intravenous infusion for 4 to 8w | ORR 69%, CR 37%; 55%, CR 36% (DLBCL); 80%, CR 40% (FL); 71%, CR 42% (MCL)  mPFS 508 days (95% CI, 207-1,129) | grade ≥ 3 AEs: lymphopenia 79%, neutropenia 17%;  serious neurologic events: 22%, encephalopathy 8%; aphasia 4%; headache 3% |
| relapsed/refractory diffuse large B-cell lymphoma | Blinatumomab | CD3 × CD19 | Phase II  (NCT01741792) | Stepwise: 9-28-112 µg/d with weekly dose increases  Flat: 112 µg/d continuous infusion over 8 weeks | ORR 42.9% (CR 19%)  SD 9.5%  mOS 5.0 mo (95% CI, 2.3 - NR)  mPFS 3.7 mo (95% CI, 1.4-7.7)  mDoR 11.6 mo (95% CI, 0.9 - NR) | grade ≥ 3 AEs:  thrombocytopenia 17%; leukopenia 17%  grade 3 neurologic events: 21.7%, encephalopathy 9%; aphasia 9% |
| aggressive relapsed/refractory B-NHL (83% diffuse large B-cell lymphoma) | Blinatumomab | CD3 × CD19 | phase II/III  (NCT02910063) | cycle 1: 9 µg/day w1, 28 µg/day w2, and 112 µg/day w3-8 in 10 weeks; cycle 2: 9 µg/day w1, 28 µg/day w2 and 112 µg/ w3-4 in 4 weeks | ORR 37% (CMR 22%)  mOS 4.9 mo (95% CI, 3.5-9.7)  mPFS 2.5 mo (95% CI, 2.3-4.9) | grade ≥ 3 AEs: 71%, neutropenia 10%; anemia 7%; confusional state 7%  neurologic events 56% (grade ≥ 3: 24%) |
| relapsed/refractory heavily pretreated B-NHL | Blinatumomab | CD3 × CD19 | phase I | Blinatumomab continuous infusion (level 1 and 2: 9 µg/day to 112 µg/day) from days 1-56 and lenalidomide (level 1: 10 mg and level 2: 20 mg daily) days 29-49 of a 56-day induction cycle | ORR: 83%, CR 50%  mPFS 8.3 mo (95% CI, 2.2-NR) | grade ≥ 3 AEs: lymphopenia 39%; hypophosphatemia 22%; hyponatremia 11%; neurotoxicity 5.5% |
| relapsed/refractory heavily pretreated B-NHL | Mosunetuzumab | CD3 × CD20 | phase I  (NCT02500407) | group A: 0.05-2.8 mg d1 q3w, up to 8/17 cycle  group B: 0.4/1.0/2.8-1.0/2.0/ 60 mg, d1/8/15, q3w, up to 8/17 cycle | aggressive B-NHL: ORR 34.9% (CR 19.4%); mDoR 22.8 mo (95% CI, 7.6 - NR); mPFS 1.4 mo (95% CI, 1.4-2.9)  indolent B-NHL: ORR 66.2% (CR 48.5%); DoR 20.4 mo (95% CI, 16-NR); mPFS 11.8 mo (95% CI, 8.4-NR) | CRS 27.4% (grade ≥ 3: 1.0%)  AEs: neutropenia 28.4%; hypophosphatemia 23.4%; fatigue 22.8%; diarrhea 21.8% |
| relapsed/refractory follicular lymphoma (grade 1-3a) | Mosunetuzumab | CD3 × CD20 | Phase II  (NCT02500407) | 1mg (D1C1), 2mg (D8C1), 60mg (D15C1, D1C2), 30mg (D1C3), 3weeks as a cycle, up to 8/17 cycle | ORR 72%, CR 60%  mDoR 22.8 mo (95% CI, 9.7-NR)  mPFS 17.9 mo (95% CI, 10.1-NR)  18-mo OS 89.6% (82.5%-96.6%) | CRS 40% (grade ≥ 3: 5.0%)  grade ≥ 3 AEs: neutropenia 27%; hypophosphatemia 17%; hyperglycaemia 8%; anaemia 8%  serious adverse events 47% |
| NHL (20 pts) and CLL (5 pts) | Odronextamab (REGN1979) | CD3 × CD20 | Phase I | flat doses ranging from 0.03-3.0 mg, qw for 4 doses, q4w for 5 doses | ORR in NHL 20%  SD 2pts with CLL | CRS 28% (grade ≥ 3: 8 %)  TRAEs: pyrexia 56%; IRR 40%; chills 36%; fatigue 24%; tachycardia 24%;  grade ≥ 3 TRAEs: 32%, IRR 12%; hypotension 8% |
| B-NHL | Odronextamab (REGN1979) | CD3 × CD20 | Phase I  (NCT02290951) | doses ranging from 0.1-320 mg, qw for 12 doses, q2w maintenance dosing | FL: RP2D 80 mg, at doses of ≥5 mg, ORR 91% (CR 72%)  DLBCL: RP2D 160 mg, 1) pts without prior CAR-T therapy ORR 39% (CR 24%), mDoR 10.3m; 2) pts who were refractory to prior CAR-T therapy ORR 33% (CR 27%) | CRS 62.2% (grade ≥ 3: 7.1%)  TRAEs: pyrexia 76.4%; chills 48.0%  grade ≥ 3 AEs: anaemia 25%; lymphopenia 19%; hypophosphataemia 19%; neutropenia 19%; thrombocytopenia 14%;  treatment-related deaths 4 pts |
| B-NHL | Epcoritamab | CD3 × CD20 | phase I/II  (NCT03625037) | doses ranging from 0.0128-60 mg, subcutaneous in 28-day cycles | RP2D 48 mg  ORR 88% (CR 38%)  FL: ORR 90% (CR 50%)  DLBCL: 1) ≥12 mg ORR 68% (CR48%), mPFS 9.1 mo (95% CI, 1.6-NR); 2) ≥48 mg ORR 91% (CR 55%), mPFS NR | CRS 59% (all grade 1-2); neurotoxicity 6% (grade 3: 3%) |
| relapsed/refractory NHL | Plamotamab (XmAb13676) | CD3 × CD20 | Phase I  (NCT02924402) | A: doses ranging from 20-125 µg/kg in 28-day cycle  B: a priming dose on C1D1, followed by escalated dose on subsequent weeks | ORR 43.3% (NHL); 38.2% (DLBCL); 80% (FL) | CRS 62.5% (grade ≥ 3: 5.0%)  no related neurotoxicity grade > 2 |
| relapsed/refractory NHL | Glofitamab | CD3 × CD20 | Phase I/Ib  (NCT03075696) | 7 days before the first dose of Glofitamab, all patients received 1,000 mg Obinutuzumab, Glofitamab was given in 14 or 21-day cycles. | ORR (CR) 53.8% (36.8%), aNHL 48% (33.1), DLBCL 41.4% (28.8%); trFL 55.2% (34.5); FL 70.5% (47.7%)  aNHL: mDoR 5.5 mo, mPFS 2.9 mo  FL: mDoR 10.8 mo, mPFS 11.8 mo  RP2D 2.5 mg (C1D1), 10 mg (C1D8), and 30 mg (C2D1) | CRS 50.3% (grade ≥ 3: 3.5%)  grade ≥ 3 AEs: 56.7%, neutropenia 25.1%; thrombocytopenia 8.2%; anemia 7.6% |
| relapsed/refractory DLBCL | Tebotelimab  (MGD013) | PD-1 × LAG-3 | Phase I | 600 mg, q2w | ORR 50% (CR 14%; PR 36%) | TRAEs 64.7% |
| relapsed/refractory classical Hodgkin lymphoma | AFM13 | CD30 × CD16A | Phase I  (NCT01221571) | 0.01/0.04/0.15/0.5/1.5/  4.5/7.0 mg/kg qw | ORR: 11.5% (PR 11.5%), 23% (≥ 1.5 mg/kg)  DCR 61.5%, 77% (≥ 1.5 mg/kg) | grade ≥ 3 AEs: 28.6%, pneumonia 14.3%; anemia 7.2%; hemolytic anemia 3.6%; thrombocytopenia 3.6%; pyrexia 3.6%; hypoalbuminemia 3.6%; T-cell lymphoma 3.6% |
| relapsed/refractory classical Hodgkin lymphoma | AFM13 | CD30 × CD16A | phase II  (NCT02321592) | A: 1.5 mg/kg 3x/week for 8w  B: 1.5 mg/kg 3x/week for 2w, followed by 7.0 mg/kg, qw for 6w  C: 7 mg/kg qw | ORR 16.6%, CR 4.2%  DCR 41.7%  PFS 5.5 mo (95% CI, 2.6 -11.0)  12-mo PFS 12.6%  12-mo OS 62% | serious adverse reaction: one grade 4 and one grade 2 infusion related reaction |
| relapsed/refractory classical Hodgkin lymphoma | AFM13 | CD30 × CD16A | Phase Ib  (NCT02665650) | cohort 1: AFM 13, 0.3 mg/kg qw (w2,3), 0.5 mg/kg qw (w4-9), 0.5 mg/kg q3w (w10-25)  cohort 2: AFM 13, 1.5 mg/kg qw (w2,3), 1.5 mg/kg qw (w4-9), 1.5 mg/kg q3w (w10-25)  cohort 3: AFM 13, 9 mg/kg qw (w2,3), 7 mg/kg qw (w4-9), 7 mg/kg q3w (w10-25)  both combine with Pembrolizumab 200 mg q3w (w1-52) | ORR: 83%, CMR 37%; 85%, CMR 37% (pts refractory to BV)  mDoR: 9.9 mo (cohort 1, 95%CI, 8.4-NR); 10.4 mo (cohort 2, 95%CI, 2.8-10.4); 9 mo (cohort 3, 95%CI, 8.4-NR) | grade ≥ 3 TRAEs for AFM13: 23%, IRR 13%; gastritis 3%; hypotension 3%; increased AST 3%; nausea 3%; neutropenia 3%; vomiting 3% |
| Hematologic Malignancies  (HL 31 pts, B-NHL 15 pts, T-NHL 11 pts, MM 7 pts, PMBL 1 pts) | Ipilimumab plus Nivolumab | CTLA-4 plus PD-1 | Phase Ib  (CheckMate 039) (NCT01592370) | 3 mg/kg Nivolumab plus 1 mg/kg Ipilimumab q3w for 4 doses, followed by 3 mg/kg Nivolumab q2w for up to 2 years | ORR: 74%, CR 19% (HL), 20%, CR 0% (B-NHL), 9%, CR 0% (T-NHL)  mDoR NR (HL, range, 0-13.4)  mPFS NR (95%CI, 7.2-NR)  mOS NR | TRAEs: fatigue 26%; pyrexia 23%; diarrhea 18% (29% grade ≥ 3) |
| relapsed/refractory classical Hodgkin lymphoma | Ipilimumab plus Nivolumab | CTLA-4 plus PD-1 | Phase I  (NCT01896999) | A: Ipilimumab q6w with brentuximab vedotin q3w  B: Nivolumab 3 mg/kg, q3w with brentuximab vedotin, q3w  C: Nivolumab 3 mg/kg, q3w plus Ipilimumab 1 mg/kg, q12w with brentuximab vedotin 1.8 mg/kg, q3w | ORR:76%, CR 57% (A); 89%, CR 61% (B); 82%, CR 73% (C)  1-year PFS: 61% (A); 70% (B); 80% (C)  mPFS 1.84 years (pts with PR, 95% CI, 0.74–NR)  1-year RFS: 59% (A); 77% (B); 87% (C)  2-year OS: C＞B＞A | grade ≥ 3 TRAEs: 50%, lipase increased 10%; AST/ALT increased 10%; vomiting 9%; white blood cell decreased 9%; fatigue 9% (C, A 43%, B 16%) |

NHL, non-Hodgkin lymphoma; aNHL, aggressive NHL; B-NHL, B cell NHL; DLBCL, diffuse large B-cell lymphoma; PMBL, primary mediastinal B cell lymphoma; CLL, chronic lymphocyte leukemia; FL, follicular lymphoma; trFL, transformed follicular lymphoma; MCL, mantle cell lymphoma; MM, multiple myeloma; MTD, maximum tolerated dose; RP2D, recommended phase II dose; D1C1, d1 on cycle 1; ORR, overall response rate; CR, complete response; PR, partial response; CMR, complete metabolic response; SD, stable disease; DCR, disease control rate; OS, overall survival; PFS, progression-free survival; RFS, recurrence-free survival; DoR, duration of response; NR, not reached; mo, months; HSCT, hematopoietic stem cell transplantation; AEs, adverse events; TRAEs, treatment-related adverse events; IRR, infusion related reaction; CRS, cytokine release syndrome.

**Table S2** Clinical trials of Multiple Myeloma

| Tumor | Drug | Target | Phase (ID) | Usage | Outcomes | Adverse Events |
| --- | --- | --- | --- | --- | --- | --- |
| relapsed/refractory MM | AMG 420 | BCMA × CD3 | Phase I  (NCT02514239) | doses ranging from 0.2-800 μg/d w1-4, in 6 weeks up to 10 cycles | ORR 31%, CR 21%  DoR 8.4 mo (2.5-≥15.5 mo)  mOS 32 mo (95% CI, 4 -59)  mPFS 23.5 mo  MTD: 400 μg/d (ORR 70%, MRD-negative CR 50%) | CRS 38% (grade ≥ 3: 2.3%)  serious AEs: 48%, infections 34%; polyneuropathy 4.8%;  no grade ≥ 3 CNS toxicities |
| relapsed/refractory MM | AMG 701 | BCMA × CD3 | Phase I/II  (NCT03287908) | 3 cohorts: 1) dose 5-45 μg; 2) 0.14-1.2 mg; 3) 1.6-12 mg, qw in 4 weeks | ORR: 36% (3-12 mg), sCR n=4; CR n=1; VGPR n=6; PR n=6  mDoR 3.8 mo (95% CI, 1.9-7.4) | CRS 61% (grade ≥ 3: 7%)  AEs: anemia 43%; diarrhea 31%; fatigue 25%; fever 25% neutropenia 23%; thrombocytopenia 20%  treatment-related neurotoxicity 6 pts |
| relapsed/refractory MM | PF-06863135 | BCMA × CD3 | Phase I  (NCT03269136) | IV: 50 μg/kg, IV, qw  SC: 80, 130, 215, and 360 μg/kg, SC, qw | IV: CBR 41%, minimal response n=1; SD n=6  SC: ORR 33%, PR n=2; VGPR n=2; SD n=7 | IV: TRAEs 59%, CRS 24%; thrombocytopenia 24%; anemia 18%; pyrexia 18% (grade ≥ 3 TRAEs: 18%)  SC: CRS 61% |
| relapsed/refractory MM | REGN5458 | BCMA × CD3 | Phase I  (NCT03761108) | 3−96 mg, IV, qw, maintenance phase IV, q2w | ORR: 35.6% (60% at highest dose), CR 31.3%, VGPR 81.3%  DoR: 43.8% >4 mo; 18.8% >8 m | TRAEs: CRS 37.8%; fatigue 17.8%; nausea 17.8%; myalgias 13.3 (grade ≥ 3 TRAEs: 28.9%)  grade ≥ 3 treatment-related neurological events 1 pts |
| relapsed/refractory MM | Teclistamab | BCMA × CD3 | Phase I  (NCT03145181) | 0.3-720 µg/kg, IV  20-3000 µg/kg, SC | ORR 65% (RP2D), CR 40%  ORR 61% (in triple-class refractory pts)  6-mo PFS 67% (RP2D)  RP2D: 1500 µg/kg, SC | CRS 57%  grade ≥ 3 TEAEs: 85%, neutropenia 48%; anaemia 33%; thrombocytopenia 23%; leukopenia 15%; |
| relapsed/refractory MM (77.6% had triple-class refractory disease) | Teclistamab | BCMA × CD3 | Phase I, SC cohort  (NCT03145181)  Phase II  (NCT04557098) | 1.5 mg/kg, SC, qw after received first step-up doses of 0.06 mg/kg and 0.3 mg/kg (separated by 2-4 days and were completed 2-4 days before 1.5 mg/kg, SC) | ORR: 63%, CR 39.4%, MRD-negativity 46%  DoR 18.4 mo (95% CI, 14.9 to NR)  mPFS 11.3 mo (95% CI, 8.8 to 17.1)  mOS 18.3 mo (95% CI, 15.1-NR) | CRS 72.1% (grade ≥ 3: 0.6%)  grade ≥ 3 AEs: neutropenia 64.2%; infections 44.8%; anemia 37.0%; neurotoxic 14.5%; (immune effector cell–associated neurotoxicity syndrome 3.0%) |
| relapsed/refractory MM | TNB-383B | BCMA × CD3 | Phase I  (NCT03933735) | 0.025-40 mg, IV, q3w | ORR 52% (≥ 5.4 mg), CR 3 pts, VGPR 3 pts, PR 6 pts  mDoR 9 weeks (95% CI, 3-21) | grade ≥ 3 AEs: anemia 16%; thrombocytopenia 13%  drug-related AEs: CRS 21%; headache 13% |
| relapsed/refractory MM | Cevostamab (BFCR4350A) | FcRH5 × CD3 | Phase I  (NCT03275103) | 0.05-3.6mg (C1D1), 0.15-132mg (C1D8、D1), IV, q3w | ORR 51.7% (≥ 3.6/20 mg), sCR10.3%, CR 10.3%, VGPR 13.8% | CRS 74.5%  grade ≥ 3 TRAEs: 39.2%, lymphopenia 11.8%; neutropenia 9.8%; anemia 5.9%; thrombocytopenia 5.9% |
| relapsed/refractory MM (79% triple-class refractory) | Talquetamab  (JNJ-64407564) | GPRC5D × CD3 | Phase I  (NCT03399799) | IV: 0.5-180 µg/kg  SC: 5-800 µg/kg | IV: ORR 78% (20-180 µg/kg)  SC: ORR 67% (135-405 µg/kg); 70%, ≥ VGPR 57% (405 µg/kg); 71%, ≥ VGPR 53% (800 µg/kg)  6-mo DoR 67% (405 µg/kg)  RP2D: 405 µg/kg qw, SC (origin), 800 µg/kg q2w, SC | CRS 78% (RP2D)  ≥ grade 3 TRAEs: lymphopenia 37%; anemia 27%; neutropenia 25% |

MTD, maximum tolerated dose; RP2D, recommended phase II dose; D1C1, day1 on cycle 1; SC, subcutaneous injection; IV, intravenous injection; ORR, overall response rate; CR, complete response; sCR, stringent CR; PR, partial response; VGPR, very good PR; CMR, complete metabolic response; SD, stable disease; DCR, disease control rate; CBR, clinical benefit rate; OS, overall survival; PFS, progression-free survival; DoR, duration of response; NR, not reached; mo, months; MRD, Minimal residual disease; AEs, adverse events; TRAEs, treatment-related adverse events; TEAEs, treatment-emergent AEs; CRS, cytokine release syndrome; CNS, central nervous system.

**Table S3** Clinical trials of Lung Cancer

| Tumor | Drug | Target | Phase (ID) | Usage | Outcomes | Adverse Events |
| --- | --- | --- | --- | --- | --- | --- |
| EGFR Exon20ins NSCLC | Amivantamab | EGFR × cMet | Phase I  NCT02609776 | 1050 mg (1,400 mg, ≥ 80 kg) qw up to w4, q2w start at w5 | ORR 40%  mDoR 11.1 mo  mPFS 8.3 mo  mOS 22.8 mo | AEs associated with EGFR inhibition: rash 86%; paronychia 45%; stomatitis 21%; pruritus 17%; diarrhea 12%  AEs associated with MET inhibition: hypoalbuminemia 27%; peripheral edema 18% |
| advanced NSCLC | Bintrafusp alfa (M7824) | TGF-β × PD-L1 | Phase I  NCT02517398 | 500 mg/1200 mg (RP2D), q2w | ORR: 17.5%, 25.0% (RP2D), 85.7% (PD-L1 ≥80%)  mOS 15.6 mo (RP2D) | TRAEs: 47.4%, hyperthyroidism 10.5%; hypothyroidism 15.8%; keratoacanthoma 10.5%; nausea 10.5%; rash maculopapular 15.8%; vomiting 10.5% |
| NRG-1^+^ cancers  (**NSCLC 41 pts**; pancreas cancer 18 pts; BC 5 pts; cholangiocarcinoma 3 pts; colorectal cancer 2 pts; other tumors 4 pts) | Zenocutuzumab (MCLA-128) | HER2 × HER3 | phase I/ II  (NCT02912949) | 750 mg, q2w in 4-week cycles | ORR 34% (**NSCLC 35%**; pancreas cancer 39%; BC 50%; cholangiocarcinoma 33.3%)  mDOR 9.1 mo | grade ≥ 3 events were reported in <5% |
| advanced solid tumors | AK112 | PD-1 × VEGF-A | Phase I  (NCT04047290) | 0.3-30 mg/kg, IV, q2w | ORR 23.5% (≥ 3 mg/kg)  DCR 64.7% (≥ 3 mg/kg) | TRAEs: 55.2%, arthralgia 17%; diarrhea 14%; rash 10%; fatigue 10.3% (grade ≥ 3: 10.3%) |
| advanced NSCLC | AK112 | PD-1 × VEGF-A | phase Ib/II  (NCT04900363) | A: 10 mg/kg q3w  B: 20 mg/kg q2w  C: 20 mg/kg q3w  D: 30 mg/kg q3w | ORR/DCR: 22.2%/88.9% (A); 44.0%/92.0% (B); 37.9%/93.1% (C); 100%/100% (D); 42.9%/ 92.9% (>10 mg/kg q3w); 56.3%/100% (TPS ≥ 1%); 23.5%/ 76.5% (TPS < 1%) | TRAEs: proteinuria 17.0%; hypertension 16.0%; lipase increase 12.8%; alanine aminotransferase increase 12.8%; blood urea increase 10.6%; apolipoprotein E increase 10.6%; hyperglycaemia 10.6% (grade ≥ 3: 10.6%) |
| advanced NSCLC with negative driver genes | AK112 | PD-1 × VEGF-A | phase II  (NCT04736823) | 10mg/kg or 20mg/kg AK112 combination with chemotherapy | pts with wide-type EGFR/ALK: ORR 76.9% (PR 76.9%), 77.8% (squamous NSCLC); DCR 100.0%, 100% (squamous NSCLC); 6-mo PFS 86.2%, 83.3% (squamous NSCLC)  pts with EGFR mutations: ORR 68.4% (PR 68.4%); DCR 94.7%; mDoR 5.5 mo; mPFS 8.3 mo  pts progressed after anti-PD-1/L1 and platinum-based chemotherapy: ORR 40% (PR 40%); DCR 80% 6-mo PFS 71.1% | TEAE: 86.5%, AST/ALT increased; epistaxis; anemia; vomiting; nausea; rash; leukopenia; thrombocytopenia; and neutropenia (grade ≥ 3: 28.6%) |
| metastatic NSCLC | KN046 | CTLA-4 × PD-1 | phase II | A: 3 mg/kg, q2w  B: 5 mg/kg q2w | ORR/DCR: 10.7%/82.1% (A), 15.6%/62.5% (B)  mPFS 3.7 mo (range, 2.9-7.3); 7.3 mo (squamous NSCLC, range, 3.7-NR)  3, 6 and 9-mo PFS: 64.1%, 36.6% and 34.2%; 80.0%, 55.9% and 46.6% (squamous NSCLC)  3, 6 and 9-mo OS: 91.4%, 86.9% and 81.0%; 100.0%, 88.2% and 88.2% (squamous NSCLC) | TRAEs: AST/ALT increased 23.8%; infusion related reaction 25.4%; anemia 22.2%; rash 13, 20.6%; hyperglycemia 19.0%; abnormal hepatic function 15.9%; hypothyroidism 15.9%; asthenia 12.7%; pruritus 11.1% (grade ≥ 3: 33.3%) |
| advanced NSCLC | Ipilimumab plus Nivolumab | CTLA-4 plus PD-1 | phase I  (CheckMate 012)  (NCT01454102) | A: 3 mg/kg Nivolumab q2w plus 1 mg/kg Ipilimumab q12w  B: 3 mg/kg Nivolumab q2w plus 1 mg/kg Ipilimumab q6w | ORR: 47% (A); 38% (B)  mPFS: 8.1 mo (A, 95%CI, 5.6-13.6); 3.9 mo (B, 95%CI, 2.6-13.2)  1-year OS: NR (A); 69% (B) | grade ≥ 3 TRAEs in A cohort: 37%, increased lipase 8%; pneumonitis 5%; adrenal insufficiency 3%; colitis 3%  grade ≥ 3 TRAEs in B cohort: 33%, adrenal insufficiency 5%; colitis 5%; pneumonitis 3% |
| advanced NSCLC | Ipilimumab plus Nivolumab | CTLA-4 plus PD-1 | phase III  (NCT02477826) | A: 3 mg/kg Nivolumab q2w plus 1 mg/kg Ipilimumab q6w  B: platinum doublet chemotherapy based on tumor histologic type q3w for up to 4 cycles | ORR: 45.3% (A); 26.9% (B)  mPFS: 7.2 mo (A, 95%CI, 5.5-13.2); 5.5 mo (B)  1-year PFS: 42.6% (A); 13.2% (B)  Pts with PD-L1 ≥ 1%: mOS 17.1 mo (A, 95%CI, 15-20.2), 14.9 mo (B); 5-year OS 24% (A), 14% (B); mDoR 24.5 mo (A), 6.7 mo (B)  Pts with PD-L1 ＜1%: mOS 17.4 mo (A, 95%CI, 13.2-22), 12.2 mo (B); 5-year OS 19% (A), 7% (B); mDoR 19.4 mo (A), 4.8 mo (B) | grade ≥ 3 TRAEs in A cohort: 31.2%, diarrhea 17.2%, rash 17%, pruritus 14.4%; serious event 17.7%; event leading to discontinuation 12.0%; rash 1.6%; diarrhea 1.6%; anemia 1.6%  grade ≥ 3 TRAEs in B cohort: 36.1% |
| advanced NSCLC | Tremelimumab plus Durvalumab | CTLA-4 plus PD-L1 | phase III  (NCT02453282) | A: 20 mg/kg Durvalumab q4w  B: 20 mg/kg Durvalumab q4w plus 1 mg/kg Tremelimumab q4w, up to 4 doses  C: platinum-based doublet chemotherapy | mOS 11.9 mo (B, 95%CI, 9-17.7)  24-mo OS 35.4% (B)  mPFS: 3.9 mo (B, 95%CI, 2.8-5); 5.4m (C)  pts with PD-L1 expression ≥25%: mOS 16.3 mo (A), 12.9 mo (C); 24-mo OS 38.3% (A), 22.7% (C)  pts with bTMB ≥20 mut/mb: mOS 21.9 mo (B, 95%CI, 11.4-32.8); 10.0 mo (C) | TRAEs in B cohort: fatigue 12.7%; diarrhea 12.7%; pruritus 12.7%; rash 10.5%; decreased appetite 8.6%; nausea 7.5%  grade ≥ 3 TRAEs: 14.9% (A); 22.9% (B); 33.8% (C) |
| advanced NSCLC | Tiragolumab plus Atezolizumab | TIGIT plus PD-L1 | phase II  (NCT03563716) | TA: 600 mg Tiragolumab plus 1200 mg Atezolizumab on d1 of every 3-week cycle  PA: placebo plus 1200 mg Atezolizumab d1 of every 3-week cycle | ORR: 37.3% (TA); 16.2% (PA)  mPFS: 5.4 mo (TA); 3.6 mo (PA) | TRAEs, grade ≥ 3: 80.6%, 14.9% (TA); 72%, 19.1% (PA)  AEs leading to treatment withdrawal: 7.5% (TA); 10.3% (PA) |
| advanced/ recurrent NSCLC | Ipilimumab plus Nivolumab | CTLA-4 plus PD-1 | phase III  (CheckMate 9LA)  (NCT03215706) | A: 360 mg Nivolumab q3w plus 1 mg/kg Ipilimumab q6w combined with histology-based, platinum doublet chemotherapy q3w, for 2 cycles  B: chemotherapy alone q3w, for 4 cycles | ORR: 38.2%, CR 2% (A), 24.9%, CR 1% (B)  mDoR: 11.3 mo (A); 5.6 mo (B)  mOS: 15.6 mo (A, 95%CI, 13.9-20); 10.9 mo (B)  12-mo OS: 63% (A); 47% (B)  2-year OS: 38% (A); 26% (B)  2-year OS: 27% (A); 19% (B) | grade ≥ 3 TRAEs: neutropenia 7%; anaemia 6%; increased lipase 6%; diarrhoea 4%; asthenia 1% |
| operable NSCLC | Ipilimumab plus Nivolumab | CTLA-4 plus PD-1 | phase II  (NCT03158129) | A: 3 mg/kg neoadjuvant Nivolumab q14d (d1/15/29)  B: 3 mg/kg Nivolumab q14d (d1/15/29) plus 1 mg/kg Ipilimumab d1, followed by surgical resection | ORR 19% both  MPR: 24%, pCR 10% (A); 50%, pCR 38% (B) | grade ≥ 3 TRAEs: 13% (A); 10% (B) |
| advanced SCLC after first-line platinum-based chemotherapy | Ipilimumab plus Nivolumab | CTLA-4 plus PD-1 | phase I/ II  (CheckMate 032)  (NCT01928394) | A: 3 mg/kg Nivolumab q2w  B: 1 mg/kg Nivolumab plus 3 mg/kg Ipilimumab q3w for 4 cycles, followed by 3 mg/kg Nivolumab q2w | initial treatment arms: ORR 11% (A), 25% (B); DCR 36% (A), 49% (B); mOS 4.1 mo (A), 7.9 mo (B); 1-year OS 30% (A), 42% (B)  long-term follow-up arms: ORR 11.6% (A, CR 1.4%), 21.9% (B, CR 2.1%); mPFS 1.4 mo (A), 1.5 mo (B, 95%CI, 1.4-2.2); mOS 5.7 mo (A), 4.7 mo (B, 95%CI, 3.1-8.3); mOS in TMB high pts: 6.6 mo (A); 10.7 mo (B, 95%CI, 1.8-23.6);1-year OS: 30.5% (A); 30.2% (B); 2-year OS: 17.9% (A); 16.9% (B) | TRAEs, grade ≥ 3: 79%, 12.9% (A); 68.8%, 37.5% (B)  grade ≥ 3 TRAEs in B cohort: AST/ALT increased 10.4%; diarrhea/colitis 9.4%; lipase increased 5.2%; amylase increased 4.2%; rash 4.1%; pneumonitis 3.1% |

NSCLC, non-small cell lung cancer; SCLC, small cell lung cancer; BC, breast cancer; NRG-1, Neuregulin-1; RP2D, recommended phase II dose; ORR, overall response rate; CR, complete response; MPR, major pathologic response; DCR, disease control rate; OS, overall survival; PFS, progression-free survival; DoR, duration of response; NR, not reached; mo, months; TPS, tumor cell proportion score; bTMB, blood tumor mutation burden; mut/mb, mutations per megabase; AEs, adverse events; TRAEs, treatment-related adverse events; TEAEs, treatment-emergent AEs.

**Table S4** Clinical trials of the Digestive System Tumors

| Tumor | Drug | Target | Phase (ID) | Usage | Outcomes | Adverse Events |
| --- | --- | --- | --- | --- | --- | --- |
| peritoneal carcinoma | Catumaxomab | EpCAM × CD3 | Phase I/II | day 0: 10 µg; day 3: 10 or 20 µg; day 7: 30, 50, or 100 µg; day 10: 50, 100, or 200 µg | ORR: 23.5%, CR 1pts, PR 3pts  mOS 16.7 mo  MTD 10/20/50/200 µg (d0/3/7/10) | TRAEs: fever; vomiting; abdominal pain; skin toxicity; nausea |
| peritoneal carcinoma and gastric cancer | Catumaxomab | EpCAM × CD3 | Phase II  (NCT01504256) | A: Catumaxomab 10/20/50/150 µg (d0/3/7/10), 7 days after flowing by 6 cycles of FLOT  B: FLOT alone | mCR 27%  mPFS 6.7 mo  mOS 13.2 mo | grade ≥ 3 TRAEs: nausea 15%; infection 23%; abdominal pain 31%; elevated liver enzymes (GGT 31%, bilirubin 23%) |
| epithelial ovarian cancer who recived cytoreductive surgery | Catumaxomab | EpCAM × CD3 | Phase II | 10 µg intraoperatively; 10/20/50/150 µg (d7/10/13/16)  postoperatively | 24-mo OS 85%  24-mo PFS 56% | postoperative omplication rate 51%  grade ≥ 3 AEs: 56%, abdominal pain 12%; pleural effusion 12%; GGT increase 10% |
| relapsed/refractory epithelial ovarian cancer | Catumaxomab | EpCAM × CD3 | Phase IIa  (NCT00189345) | low: 10/10/10/10 µg (d0/3/7/10)  high: 10/20/50/100 µg (d0/3/7/10) | high dose: SD 23%; mPFS 70 days; mOS 185 days  low dose: SD 9%; mPFS 68 days; mOS 137 days | grade ≥ 3 AEs 7%  TEAEs: gastrointestinal and injection-site reactions |
| esophageal adenocarcinoma | Bintrafusp alfa  (M7824) | PD-L1 × TGF-β | phase I  (NCT02517398) | 1200 mg q2w | ORR: 20%, 13.3% (investigator-assessed)  DCR 33.3%  mDoR 4.3 mo (95%CI, 1.3-8.3)  mPFS 1.3 mo (95%CI, 1-5.3)  6-mo PFS 25.9%  mOS 3.4 mo (95%CI, 2.5-10.3)  6-mo OS 39.7%; 12-mo OS 32.1% | TRAEs: 63.3%  grade ≥ 3 TRAEs: 23.3%, anemia 6.7%, Bowen’s disease 3.3%, cancer pain 3.3%, gastritis hemorrhagic 3.3%, hypophysitis 3.3%, hypopituitarism 3.3%, rash 3.3%, SCC of skin 3.3% |
| advanced esophageal squamous cell carcinoma | Ipilimumab plus Nivolumab | CTLA-4 plus PD-1 | phase III  (CheckMate-648)  (NCT03143153) | A: 240 mg Nivolumab q2w plus chemotherapy (fluorouracil + cisplatin q4w)  B: 3 mg/kg Nivolumab q2w plus 1 mg/kg Ipilimumab q6w  C: chemotherapy | ORR: 47% (A); 28% (B); 27% (C)  ≥12-mo DoR: 39% (A); 48% (B); 23% (C)  mPFS: 5.8 mo (A); 2.9 mo (B, 95%CI, 2.7-4.2); 5.6 mo (C)  24-mo PFS: 11% (A); 12% (B); 4% (C)  mOS: 12.8 mo (A); 12.7 mo (B, 95%CI, 11.3-15.5); 10.7 mo (C)  24-mo OS: 29% (A); 32% (B); 19% (C)  pts with PD-L1 CPS ≥ 1%: mPFS 4 mo (B, 95%CI, 2.3-4.4); mOS 13.1 mo (B, 95%CI, 11.2-17.4) | grade ≥ 3 TRAEs: 49% (A); 33% (B); 36% (C)  select TRAEs: skin 34%; endocrine 27%; hepatic 13%; gastrointestinal 12%; pulmonary 8%; renal 2% |
| gastrointestinal adenocarcinomas (colorectal 28 pts, pancreatic 6 pts, other tumors 5 pts) | MEDI-565 | CEA × CD3 | phase I  (NCT01284231) | A: 0.75–20 μg, d1-5 in 28-day cycles  B: 60 μg–3 mg | DCR 28% (SD 28%)  MTD 5 mg  mPFS 1.9 mo (range, 0-9.7)  mOS 5.5 (range, 0.1-27.7) | grade ≥ 3 TRAEs: 12.3%, seen in 5 pts: hypoxia; diarrhea; CRS; increased alanine aminotransferase; hypertension |
| HER2^+^ GC/GEJ | KN026 | HER2 domains ECD2 × HER2 domains ECD4 | preliminary efficacy (NCT03925974) | 10 mg/kg qw, 20 mg/kg q2w or 30 mg/kg q3w | HER2 high expression: ORR 55.6%; DCR 72.2%; 9-mo PFS 60.4%  prior-HER2 therapy: ORR 44.4%; DCR 66.7%; mPFS 5.6 mo; mOS 11 mo  HER2 low expression: ORR and DCR 22.2%; mPFS 1.4 mo; mOS 9.6 mo | TRAEs: aspartate aminotransferase increased 5.8%; rash 19.4%; anaemia 16.1%; alanine aminotransferase increased 12.9%; weight decreased 12.9%  grade ≥ 3 trTEAEs: infusion related reaction 3.2%; blood pressure increased 3.2%; ureteral stricture with hydronephrosis 3.2% |
| HER2^+^ GC/GEJ | KN026 | HER2 domains ECD2 × HER2 domains ECD4 | phase II  (NCT03925974) | 10 mg/kg qw, 20 mg/kg q2w or 30 mg/kg q3w | HER2 high expression: ORR 56% (PR 50%); mDoR 9.7 mo (95%CI, 4.2-NR); mPFS 8.3 mo (95%CI, 4.2-11.4); mOS 16.3 mo (95%CI, 11-NR)  HER2 low expression: ORR 14%; mDoR 6.2 mo (95%CI, 3.2-NR); mPFS 1.4 mo (95%CI, 1.4-4.1); mOS 9.6 mo (95%CI, 3.5-14.9) | TRAEs: 82%, increased aspartate aminotransferase 27%; increased alanine aminotransferase 20%rash 16%; anemia 16%; infusion-related reaction 16% |
| HER2^+^ GC/GEJ | Zanidatamab (ZW25) | HER2 domains ECD2 × HER2 domains ECD4 | phase Ib/ II  (NCT04276493) | A: ZW25 30 mg/kg; B: ZW25 1800 mg (2400 mg weight ≥ 70 kg), both with TIS 200 mg, IV and capecitabine/oxaliplatin (CAPOX) q3W | ORR 72.7% (CR 3.0%; PR 69.7%)  mPFS 10.9 mo (95% CI, 6.9-NR) | grade ≥ 3 TEAEs 72.7%  grade ≥ 3 trTEAEs 60.6% |
| unresectable advanced GC/GEJ | Cadonilimab (AK104) | PD-1 × CTLA-4 | phase Ib/II  (NCT03852251) | AK104 (4 mg/kg, 6 mg/kg, 10 mg/kg, q2w or 10 mg/kg, 15mg/kg q3w) + chemo (mXELOX q2w or XELOX q3w) | ORR 65.9%  DCR 92%  mDoR 6.9 mo (95%CI, 4.6-11.2)  mPFS 7.1 mo (95%CI, 5.55-10.48)  mOS 17.4 mo (95%CI, 12.35-NR), PD-L1 (CPS＜1) 14.6 mo, PD-L1 (CPS ≥ 1) 17.4 mo | TRAEs: 97.9%, platelet count decreased 60.4%; white blood cell count decreased 58.3%; neutrophil count decreased 56.3%; anaemia 47.9%; nausea 30.2%; vomiting 30.2%; aspartate aminotransferase increased 30.2% (grade ≥ 3: 62.5%) |
| advanced or metastatic gastric, esophageal, or GEJ adenocarcinoma | Ipilimumab plus Nivolumab | CTLA-4 plus PD-1 | phase I/II  (CheckMate-032) | A: 3 mg/kg Nivolumab q2w, followed by 3 mg/kg Nivolumab q2w  N1I3: 1 mg/kg Nivolumab plus 3 mg/kg Ipilimumab q3w for 4 cycles, followed by 3 mg/kg Nivolumab q2w  N3I1: 3 mg/kg Nivolumab plus 1 mg/kg Ipilimumab q3w for 4 cycles, followed by 3 mg/kg Nivolumab q2w | ORR: 12% (A); 24% (N1I3); 8% (N3I1)  DCR: 32% (A); 41% (N1I3); 37% (N3I1)  mDoR: 7.1 mo (A); 7.9 (N1I3, 95%CI, 2.8-NR); NR, (N3I1, 95%CI, 2.5-NR)  mPFS: 1.4 mo (A); 1.4 mo (N1I3, 95%CI, 1.2-3.8); 1.6 mo (N3I1, 95%CI, 1.4-2.6)  12-mo PFS: 8% (A); 17% (N1I3); 10% (N3I1)  mOS: 6.2 mo (A); 6.9 mo (N1I3, 95%CI, 3.7-11.5); 4.8 mo (N3I1, 95%CI, 3-8.4)  12-mo OS: 39% (A); 35% (N1I3); 24% (N3I1) | TRAEs: fatigue; pruritus; rash; diarrhea; decreased appetite; increased ALT and AST levels  grade ≥ 3 TRAEs: 17% (A); 47% (N1I3); 27% (N3I1) |
| advanced or metastatic gastric, esophageal, or GEJ adenocarcinoma | Ipilimumab plus Nivolumab | CTLA-4 plus PD-1 | phase III  (CheckMate-649) (NCT02872116) | A: 360 mg q3w/240 mg q2w Nivolumab plus chemotherapy (CapeOX/FOLFOX)  B: 1 mg/kg Nivolumab plus 3 mg/kg Ipilimumab q3w for 4 cycles, followed by 240 mg Nivolumab q2w | ORR: 58%, CR 11% (A); 23%, CR 6% (B);  mDoR: 8.5 mo (A); 13.8 mo (B, 95%CI, 9.4-17.7)  mPFS: 7.7 mo (A); 2.8 mo (B, 95%CI, 2.6-3.6)  24-mo PFS: 16% (A), 8% (B)  mOS: 13.8 mo (A); 11.7 mo (B, 95%CI, 9.6-13.5)  12-mo OS: 49% (B)  24-mo OS: 31% (A); 25% (B)  pts with PD-L1 CPS ≥ 5: ORR: 60%, CR 13% (A), 27%, CR 5% (B); mDoR: 9.7 mo (A), 13.2 mo (B, 95%CI, 8.3-18.3); PFS 8.1 mo (A), 2.8 mo (B, 95%CI, 2.6-4); 24-mo PFS 19% (A), 10% (B); mOS: 14.4 mo (A); 11.2 mo (B, 95%CI, 9.2-13.4) | grade ≥ 3 TRAEs: 60% (A); 38% (B)  The most common grade 3/4 TRAEs: neutropaenia 15% (A); increased lipase 7% (B) |
| resectable GC/GEJ adenocarcinoma | Ipilimumab plus Nivolumab | CTLA-4 plus PD-1 | phase II  (NCT04006262) | 240 mg Nivolumab q2w for 6 times plus 1 mg/kg Ipilimumab q6w for 2 times, followed by surgery and adjuvant 480 mg Nivolumab q4w for 9 times | pCR: 58.6%  at 24 months 97% were alive and free of recurrence/progression | grade ≥ 3 TRAEs: 19%, colitis/ileitis; hepatitis |
| unresectable hepatocellular carcinoma | Cadonilimab (AK104) | PD-1 × CTLA-4 | phase II  (NCT04444167) | AK104 (6 mg/kg q2w or 15 mg/kg q3w) + lenvatinib 8 mg (weight ≥60 kg, 12 mg) | ORR 44.4%  DCR: 77.8% (PR 8 pts, SD 6 pts) | TRAEs: increased AST 36.7%; increased ALT 36.7%; decreased platelet count 33.3%; decreased neutrophil count 30.0%; increased blood bilirubin 26.7% (grade ≥ 3: 26.7%) |
| advanced hepatocellular carcinoma | Ipilimumab plus Nivolumab | CTLA-4 plus PD-1 | phase I/II  (CheckMate 040)  (NCT01658878) | N1I3: 1 mg/kg Nivolumab plus 3 mg/kg Ipilimumab q3w for 4 doses, followed by 240 mg Nivolumab q2w  N3I1: 3 mg/kg Nivolumab plus 1 mg/kg Ipilimumab q3w for 4 doses, followed by 240 mg Nivolumab q2w  N3I1-q6w: 3 mg/kg Nivolumab q2w plus 1 mg/kg Ipilimumab q6w | ORR: 32% (N1I3); 31% (N3I1); 31% (N3I1-q6w)  mDoR: 17.5 mo (N1I3, 95%CI, 4.6-47^+^); 22.2 mo (N3I1, 95%CI, 4.2-44^+^); 16.6 mo (N3I1q6w, 95%CI, 4.1-49^+^)  mOS: 22.2 mo (N1I3, 95%CI, 9.4-NR); 12.5 mo (N3I1, 95%CI, 7.6-16.4); 12.7 mo (N3I1-q6w, 95%CI, 7.4-30.5)  OS rate in N1I3: 12-mo 61%; 24-mo 46%; 36-mo 42% | TRAEs: 94% (N1I3); 71% (N3I1); 79% (N3I1-q6w)  grade ≥ 3 TRAEs: 53%, AST/ALT increased 24%, lipase increased 12%, rash 8%, diarrhea 4% (N1I3); 29% AST/ALT increased 14%, lipase increased 6%, rash 4%, diarrhea 2% (N3I1); 31%, lipase increased 8%, AST/ALT increased 4%, diarrhea 2%  1-log increase in HBV DNA or HCV RNA from baseline: 9% of HBV-infected pts; 10% of HCV-infected patients |
| advanced hepatocellular carcinoma | Tremelimumab plus Durvalumab | CTLA-4 plus PD-L1 | phase I/II  (NCT02519348) | T300D: 300 mg Tremelimumab plus 1,500 mg Durvalumab 1 dose, followed by 1,500 mg Durvalumab q4w  D: 1,500 mg Durvalumab q4w  T: 75 mg Tremelimumab q4w  T75D: 75 mg Tremelimumab q4w plus 1,500 mg Durvalumab q4w, for 4 doses, followed by 1,500 mg Durvalumab q4w | ORR: 24.0%, CR 1.3% (T300D); 10.6% (D); 7.2% (T); 9.5%, CR 2.4% (T75D)  mPFS: 2.2 mo (T300D, 95%CI, 1.9-5.4); 2.1 mo (D); 2.7m (T); 1.9 mo (T75D, 95%CI, 1.8-2.5)  mOS: 18.7 mo (T300D, 95%CI, 10.8-27.3); 13.6 mo (D); 15.1 mo (T); 11.3 mo (T75D, 95%CI, 8.4-15) | grade ≥ 3 TRAEs: 37.8%, AST/ALT increased 16.3%, lipase increased 6.8%, amylase increased 6.8%, rash 4.1%, diarrhea 1.4% (T300D); 20.8% (D); 43.5% (T); 24.4%, AST/ALT increased 10.9%, lipase increased 4.9%, diarrhea 1.2%, amylase increased 1.2% (T75D) |
| advanced hepatocellular carcinoma | Tremelimumab plus Durvalumab | CTLA-4 plus PD-L1 | phase III  (NCT03298451) | T300D: 300 mg Tremelimumab plus 1,500 mg Durvalumab 1 dose, followed by 1,500 mg Durvalumab q4w  D: 1,500 mg Durvalumab q4w  S: 400 mg sorafenib bid | ORR: 20.1% (T300D); 17% (D); 5.1% (S)  mPFS: 3.8 mo (T300D, 95%CI, 3.7-5.3); 3.7 mo (D); 4.1 mo (S)  mOS: 16.4 mo (T300D, 95%CI, 14.2-19.6); 16.6 mo (D); 13.8 mo (S)  24/36-mo OS: 40.5%/ 30.7% (T300D); 39.6%/24.7% (D); 32.6%/20.2% (S) | grade ≥ 3 TRAEs: 34% (T300D); 17% (D); 37.7% (S) |
| biliary tract cancer | Zanidatamab (ZW25) | HER2 domains ECD2 × HER2 domains ECD4 | phase I  (NCT02892123) | 10 mg/kg qw; 15 mg/kg qw; 20 mg/kg q2w | ORR 47%  DCR 65%  mDoR 6.6 mo (95%CI, 3.2-NR) | TRAE: 70%, diarrhea 45%; infusion-related reactions 30% |
| metastatic dMMR/ MSI-H colorectal cancer | Ipilimumab plus Nivolumab | CTLA-4 plus PD-1 | phase II  (CheckMate-142)  (NCT02060188) | 3 mg/kg Nivolumab plus 1 mg/kg Ipilimumab q3w for 4 doses, followed by 3 mg/kg Nivolumab q2w | ORR 55%  DCR 80%  9-mo PFS 76%  9-mo OS 87%  follow-up 64 mo: ORR 65%; mPFS NR (95%CI, 32.8-NR); 48-mo PFS 54%; 48-mo OS 71% | grade ≥ 3 TRAEs: 32%  TRAEs leading to discontinuation: 13% |
| pMMR/MSS metastatic colorectal cancer with MGMT silencing | Ipilimumab plus Nivolumab | CTLA-4 plus PD-1 | phase II  MAYA study | two priming cycles of oral temozolomide 150 mg/sqm qd, d1-5, q4w (first treatment part) followed 1 mg/kg Ipilimumab q8w plus 480 mg Nivolumab q4w (second treatment part) | ORR 45%  mDOR 4.8 mo  mPFS 7.0 mo (95% CI, 5.5-8.3)  8-mo PFS 36%; 12- mo PFS 24%; 18-mo PFS 20%  mOS 18.4 mo (95% CI, 14.9-NR) | grade ≥ 3 AEs: skin rash 6%; colitis 3%; hypophysitis 3%  Immune-related AEs: skin rash 18%; colitis 18%; hypothyroidism 21%; hyperthyroidism 9%; hypophysitis 6%; adrenal insufficiency 3% |
| NRG-1^+^ cancers  (NSCLC 41 pts; **pancreas cancer 18 pts**; BC 5 pts; cholangiocarcinoma 3 pts; colorectal cancer 2 pts; other tumors 4 pts) | Zenocutuzumab (MCLA-128) | HER2 × HER3 | phase I/ II  (NCT02912949) | 750 mg, q2w in 4-week cycles | ORR 34% (NSCLC 35%; **pancreas cancer 39%**; BC 50%; cholangiocarcinoma 33.3%)  mDOR 9.1 mo | grade ≥ 3 events were reported in <5% |

NSCLC, non-small cell lung cancer; NRG-1, Neuregulin-1; GC/GEJ, gastric carcinoma/gastro-oesophageal junction adenocarcinoma; MTD, maximum tolerated dose; ORR, overall response rate; CR, complete response; mCR, macroscopic complete remission; SD, stable disease; DCR, disease control rate; OS, overall survival; PFS, progression-free survival; DoR, duration of response; NR, not reached; mo, months; PD-L1 CPS, PD-L1 combined positive score; AEs, adverse events; TRAEs, treatment-related adverse events; TEAEs, treatment-emergent AEs; trTEAEs, treatment-related TEAES; GGT,γ-glutamyltransferase.

**Table S5** Clinical trials of Breast Cancer

| Tumor | Drug | Target | Phase (ID) | Usage | Outcomes | Adverse Events |
| --- | --- | --- | --- | --- | --- | --- |
| HER2/neu^+^ tumors progressing after standard therapy | Ertumaxomab | HER2 × CD3 | Phase I  (NCT01569412) | ascending doses (10 - 500 μg), qw, w1-4 then treatment-free, w5-7 | CBR: 27%, PR 1pts (BC); SD 2pts (rectum cancer, head and neck cancer) | AEs: fatigue 100%; tumor-pain 93%; cephalgia 86%; chills 79%; nausea 62%; fever 50%; emesis 50% |
| metastasis breast cancer | Ertumaxomab | HER2 × CD3 | Phase I | Three ascending doses (10-200μg) on d1, 7±1, and 13±1 | CBR: 33% (CR 1pts; PR 2 pts; SD 2 pts)  MTD 100 μg | AEs: fever 94%; rigors 47%; headache 35%; nausea 29%; vomiting 29%  grade ≥ 3 TEAEs: lymphocytopenia 76%; elevation of liver enzymes 47% |
| HER2^+^ cancer (BC 17 pts, GE 11 pts and other cancers 5 pts) | Zanidatamab (ZW25) | HER2 domains ECD2 × HER2 domains ECD4 | phase I  (NCT02892123) | 10 mg/kg qw; 15 mg/kg qw; 20 mg/kg q2w | DCR: 54%, PR 46% (BC); 57%, PR 43% (GE); 33% (other cancers)  RP2D: 10 mg/kg, qw or 20 mg/kg, q2w | AEs: diarrhea; infusion reaction |
| pre-treated advanced HER2^+^ breast cancer | Zanidatamab (ZW25) | HER2 domains ECD2 × HER2 domains ECD4 | phase Ib/ II  (NCT04276493) | A: zani 30 mg/kg + docetaxel 75 mg/m^2^, q3W  B: zani 1800 mg + docetaxel 75 mg/m^2^, q3W | ORR: 86.4%, CR 4.5%; 88.9, CR 11.1% (A); 84.6%, CR 0 (B)  6-mo PFS 90.9% | trTEAEs: diarrhea 56.0%; decreased neutrophil count 52.0% (grade ≥ 3: 68.0%) |
| HER2^+^ metastasis breast cancer | KN026 | HER2 domains ECD2 × HER2 domains ECD4 | phase I  (NCT03619681) | ascending dose of 5 mg/kg qw, 10 mg/kg qw, 20 mg/kg q2w or 30 mg/kg q3w | ORR 32.1% (RP2D)  DCR 76.8% (RP2D)  RP2D: 20 mg/kg, q2w or 30 mg/kg, q3w | TRAEs: pyrexia 23.8%; diarrhea 19.0%; aspartate aminotransferase increased 15.9%; neutrophil count decreased 11.1%; white blood cell count decreased 11.1% |
| HER2^+^ metastasis breast cancer | Zenocutuzumab (MCLA-128) | HER2 × HER3 | phase I/ II  (NCT02912949) | 40 - 900 mg, q3w | ORR 12.5%  CBR 70%  RP2D 750 mg, q3w | AEs: infusion related reactions 40%; diarrhea 13%; rash 13%; fatigue 13% |
| metastasis breast cancer | Zenocutuzumab (MCLA-128) | HER2 × HER3 | phase II  (NCT03321981) | A pts with HR^+^, HER2 low mBC and progressed on a CDK4/6i: MCLA-128 (750 mg), q3w combined with last ET  B pts with HER2 amplified mBC and progressed on anti-HER2 ADCs: 750 mg MCLA-128 plus 8mg/kg trastuzumab plus 25mg/m2 vinotelbine d1,8 | A: DCR 45% (unconfirmed PR 2pts, SD 19 pts)  B: DCR 77% (CR 1 pts; PR 4 pts) | AEs: asthenia/fatigue 27%; diarrhea 25%; nausea 21% (A)  AEs: diarrhea 61%; neutropenia 61%; asthenia/fatigue 46%; nausea 29% (B) |
| NRG-1^+^ cancers  (NSCLC 41 pts; pancreas cancer 18 pts; BC 5 pts; cholangiocarcinoma 3 pts; colorectal cancer 2 pts; other tumors 4 pts) | Zenocutuzumab (MCLA-128) | HER2 × HER3 | phase I/ II  (NCT02912949) | 750 mg, q2w in 4-week cycles | ORR 34% (NSCLC 35%; pancreas cancer 39%; BC 50%; cholangiocarcinoma 33.3%)  mDOR 9.1 mo | grade ≥ 3 events were reported in <5% |
| advanced LAG-3 high expression cancer (triple negative breast cancer) | Tebotelimab (MGD013) | PD-1 × LAG-3 | Phase I  (NCT03219268) | sequential single-pt cohorts: 1-1200 mg, q2w  tumor-specific expansion cohorts: 600mg, q2w | sequential single-pt cohorts: DCR 58.5% (PR 7.3%, SD 51.2%)  tumor-specific expansion cohorts: DCR 45%, PR 6% | TRAEs: 70.5%, fatigue 19%; nausea 11% (grade ≥ 3: 23.2%) |
| HER2^+^ tumors (breast cancer) | Tebotelimab (MGD013) | PD-1 × LAG-3 | phase I  (NCT03219268) | Tebotelimab (300/600 mg) plus Margetuximab 15 mg/kg, q3w | ORR: 15% (breast cancer); 13.3% (breast cancer with anti-HER2 therapy) | TRAEs: 73.8% (grade ≥ 3: 16.7%) |

NSCLC, non-small cell lung cancer; BC, breast cancer; GE, gastric/esophageal adenocarcinoma; NRG-1, Neuregulin-1; MTD, maximum tolerated dose; RP2D, recommended phase II dose; ORR, overall response rate; CR, complete response; PR, partial response; SD, stable disease; DCR, disease control rate; CBR, clinical benefit rate; OS, overall survival; PFS, progression-free survival; DoR, duration of response; NR, not reached; mo, months; CDK4/6i, cyclin-dependent kinase 4 and 6 inhibitors; AEs, adverse events; TRAEs, treatment-related adverse events; TEAEs, treatment-emergent AEs; trTEAEs, treatment-related TEAES.

**Table S6** Clinical trials of HPV^+^ Tumors

| Tumor | Drug | Target | Phase (ID) | Usage | Outcomes | Adverse Events |
| --- | --- | --- | --- | --- | --- | --- |
| advanced cervical cancer | Cadonilimab (AK104) | PD-1 × CTLA-4 | Phase II | 6 mg/kg, q2w | ORR 33.0% (CR 12%, PR 21%)  12m-DoR 52.9%  mPFS 3.8 mo (95%CI, 2-6.4)  mOS 17.5 mo (95%CI, 11.4-NR)  12m-OS 64.4%  pts with PD-L1 CPS≥ 1: ORR 43.8%; mPFS 6.3 mo (95%CI, 3.1-11.2); mOS NR (95%CI, 17.5-NR) | grade ≥ 3 TRAEs: 28.8%, anemia 7.2%; decreased appetite 2.7% |
| recurrent/metastasis cervical cancer | Cadonilimab (AK104) | PD-1 × CTLA-4 | Phase II  (NCT04868708) | A-15: AK104 15 mg/kg + PTX 175mg/m2 + DDP 50 mg/m2/CBP AUC 5, q3w;  A-10: AK104 10 mg/kg + PTX 175mg/m2 + DDP 50 mg/m2/CBP AUC 5, q3w;  B-10: AK104 10 mg/kg + PTX 175 mg/m2 + DDP 50 mg/m2/CBP AUC 5 + bev 15 mg/kg, q3w | ORR in A-15: 73.3% (CR 26.7%, PR 46.7%)  ORR in A-10: 68.8% (CR 6.3%; PR 62.5%)  ORR in B-10: 92.3% (CR 7.7%; PR 84.6%) | grade ≥ 3 TRAEs: 51.1%, anemia 15.6%; white blood cell count decreased 11.1%; neutrophil count decreased 8.9%; platelet count decreased 8.9% |
| recurrent/metastatic HPV^+^ cervical cancer | Bintrafusp alfa (M7824) | PD-L1 × TGF-β | phase I  (NCT02517398)  phase II  (NCT03427411) | phase I dose escalation: 0.3-30 mg/kg q2w  phase II: 1200 mg q2w | ORR 28.2%  mDoR 11.7 mo  mOS 13.4 mo (95%CI, 5.5-NR)  24-mo OS 33.2% | TRAEs: 84.6%  grade ≥ 3 TRAEs: 23.1%, anemia; colitis; gastroparesis; upper gastrointestinal hemorrhage; keratoacanthoma; cystitis noninfective; hematuria, pneumonitis; rash macular; asymptomatic hypokalemia related to the above grade 3 gastroparesis |
| HPV^+^ advanced solid tumors  (pancreatic 5 pts; cervix uteri 4 pts; adenoid cystic carcinoma 2 pts; anal 2 pts; colorectal 2 pts; other tumors 4 pts) | Bintrafusp alfa  (M7824) | PD-L1 × TGF-β | phase I  (NCT02517398) | 0.3-10 mg/1 mg/3 mg/ 10 mg/20 mg/kg, q2w | pancreatic: PR 1 pts, SD 3 pts;  cervix uteri: CR 1 pts, SD 1 pts;  anal: PR 1 pts;  other tumors: SD 2 pts | TRAEs: 47.4%, hyperthyroidism 10.5%; hypothyroidism 15.8%; keratoacanthoma 10.5%; nausea 10.5%; rash maculopapular 15.8%; vomiting 10.5% (grade ≥ 3: 21.1%) |
| HPV^+^ advanced solid tumors  (cervical 25 pts, anal 4 pts) | Bintrafusp alfa  (M7824) | PD-L1 × TGF-β | phase I  (NCT02517398)  dose-escalation cohort and expansion part | 1200 mg, q2w | ORR 27.9% (CR 7%, PR 20.9%)  DCR 41.9%  mDoR 19.1 mo (95%CI, 4.2-27.4)  mPFS 2.8 mo (95%CI, 1.4-4.6)  12-mo PFS 26.2%; 18-mo PFS 23.3%  mOS 16.2 mo (95%CI, 7.1-NR)  12-mo OS 56.5%; 18-mo OS 48.8% | TRAEs: 81.4%, pruritus  23.3%; keratoacanthoma 20.9%; dermatitis acneiform 16.3%; hypothyroidism 16.3%; rash maculopapular 14.0% (grade ≥ 3: 25.6%) |
| HPV^+^ advanced solid tumors  (cervical 8 pts; anal 2 pts; rectal SCC 2 pts; other tumors 3 pts) | Bintrafusp alfa  (M7824) | PD-L1 × TGF-β | phase II  (NCT03427411) | 1200 mg, q2w | ORR 37.5% (CR 12.5%, PR 25%)  DCR 50%  mDoR NR (95%CI, 4.2-NR)  mPFS 3.3 mo (95%CI, 1.4-NR)  12-mo PFS 29.2%  mOS NR (95%CI, 3.7-NR)  12-mo OS 72.1% | TRAEs: 87.4%, pruritus  31.3%; dermatitis acneiform 31.3%; anemia 31.3%; fatigue 31.3%; rash maculopapular 18.8%; mucosal bleeding 18.8%; hypothyroidism 12.5%; stomatitis 12.5%; Influenza-­like illness 12.5% (grade ≥ 3: 31.3%) |
| SCCHN progressed/ recurred after platinum therapy | Bintrafusp alfa  (M7824) | PD-L1 × TGF-β | phase I  (NCT02517398) | 1200 mg, q2w | ORR: 16%, PR 16%; 12% (PD-L1^+^); 17% (PD-L1^-^)  DCR 34%  mDoR 18.1 mo  mPFS 1.4 mo (95% CI, 1.3-4)  6-mo PFS 28%; 12-mo PFS 21%; 18-mo PFS 21%  mOS 9.1 mo (6.6-NR)  12-mo OS 48%; 18-mo OS 44% | grade ≥ 3 TRAEs: 34%, rash maculopapular 6%, increased liver enzymes, anemia 3%, diabetic ketoacidosis 3%, colitis 3%, hyperglycemia 3%, hyperthyroidism 3% |
| HPV 16^+^ relapsed/refractory advanced cancer  (cervical 5 pts; vaginal/vulvar 2 pts; anal 4 pts; oropharyngeal 3 pts) | Bintrafusp alfa  (M7824) | PD-L1 × TGF-β | phase II  (NCT04287868) | Bintrafusp alfa at 1200 mg q2w + M9241 at 16.8 mcg/kg q4w + PDS0101 0.5 ml q4w | ORR 71% (n=14, CR 1 pts, PR 9 pts); 83% (pts with checkpoint naïve); 63% (pts with checkpoint refractory) | grade ≥ 3 TRAEs: 43.3%, anemia 30%; hematuria 10% |

SCC, squamous cell carcinoma; SCCHN, SCC of the head and neck; ORR, overall response rate; CR, complete response; PR, partial response; SD, stable disease; DCR, disease control rate; OS, overall survival; PFS, progression-free survival; DoR, duration of response; NR, not reached; mo, months; AEs, adverse events; TRAEs, treatment-related adverse events.

**Table S7** Clinical trials of Prostate Cancer

| Tumor | Drug | Target | Phase (ID) | Usage | Outcomes | Adverse Events |
| --- | --- | --- | --- | --- | --- | --- |
| metastasis castration-resistant prostate cancer | Pasotuxizumab | PSMA × CD3 | Phase I  (NCT01723475) | SC: 0.5 μg, qd  IV: 5 μg, qd | SC: 30% reductions in PSA >50%; SD 17%; mPFS 92 days (range, 43-505)  IV: 33.3% reductions in PSA >50%; SD 18.8%; mPFS 98 days (range, 68-500) | grade ≥ 3 TRAEs: 49%, lymphocyte count decreased 23%, hypophosphatemia 10%, fatigue 7% (SC); 63%, lymphocyte count decreased 44%, fever 13%, hypophosphatemia 13%, fatigue 6%, CRS 6% (IV) |
| AR-V7^+^ metastatic prostate cancer | Ipilimumab plus Nivolumab | CTLA-4 plus PD-1 | phase II | 3 mg/kg Nivolumab plus 1 mg/kg Ipilimumab q3w for 4 doses, followed by 3 mg/kg Nivolumab q2w | PSA_50_ 13%  ORR 25%  mPSA-PFS 3.0 mo (95% CI, 2-NR)  mPFS 3.7 mo (95% CI, 2.8-7.5)  mOS 8.2 mo (95% CI, 5.5-10.4)  pts with DRD^-/+^ (DRD^-^ vs DRD^+^): PSA_50_ 0% vs 33.3%; ORR 0% vs 40%; mPSA-PFS 2.1 vs 5.8 mo (95% CI,4.2-NR); mPFS 2.8 vs 6.5 mo (95% CI, 3.9-NR); mOS: 7.23 vs 9.0 mo (95% CI, 8.2-NR) | grade ≥ 3 TRAEs: 46%, fatigue; diarrhea/colitis; elevated lipase |
| metastatic castration-resistant prostate cancer | Ipilimumab plus Nivolumab | CTLA-4 plus PD-1 | phase II  (CheckMate 650)  (NCT02985957) | 1 mg/kg Nivolumab plus 3 mg/kg Ipilimumab q3w for 4 doses, followed by 480 mg Nivolumab q4w | A (pts who progressed after hormone therapy and had not received chemotherapy): ORR 25%; mPFS 5.5 mo (95%CI, 3.5-7.1); mOS 19 mo (95%CI, 11.5-NR); PSA_50_ 17.6%  B (pts who progressed after chemotherapy): ORR 10%; mPFS 3.8 mo (95%CI, 2.1-5.1); mOS 15.2 mo (95%CI, 8.4-NR); PSA_50_ 10% | TRAEs: 93.3%, diarrhea, pneumonitis, increased lipase (A); 95.6%, diarrhea, colitis (B)  grade ≥ 3 TRAEs: 42.2% (A); 53.3% (B) |

AR-V7, androgen receptor splice variant 7; SC, subcutaneous injection; IV, intravenous injection; ORR, overall response rate; OS, overall survival; PFS, progression-free survival; DoR, duration of response; NR, not reached; mo, months; DRD, DNA repair defects; PSA_50_, percentage of people with a PSA decline rate of 50% or higher; TRAEs, treatment-related adverse events.

**Table S8** Clinical trials of Melanoma

| Tumor | Drug | Target | Phase (ID) | Usage | Outcomes | Adverse Events |
| --- | --- | --- | --- | --- | --- | --- |
| metastasis uveal melanoma | Tebentafusp | gp100 × CD3 | Phase I  (NCT02570308) | 20 μg C1D1, 30 μg C1D8, 54/64/73/68μg C1D15 | ORR 11.9%  mDoR 8.5 mo (range, 3.7-8.5)  mPFS 4.6 mo (range, 0.7-25.9)  mOS 25.5 mo (95% CI, 12.4-31.1)  1y-OS 67%  RP2D: 20 μg C1D1, 30 μg C1D8, 68 μg C1D15 | CRS 90%  skin toxicity: 67%, rash 83%; pruritus 83%; dry skin 64%; pigment change 57%; erythema 57%; edema 57%; other changes 43% |
| metastasis uveal melanoma | Tebentafusp | gp100 × CD3 | Phase II  (NCT02570308) | 20 μg C1D1, 30 μg C1D8, 68μg C1D15 | ORR 5%  mDoR 8.7 mo  mPFS 2.8 mo  mOS 16.8 mo (95% CI, 12.9-21.3)  12-mo OS 62%  RP2D 20/30/68 μg | CRS 86%  TRAEs: pyrexia 80%; pruritus 67%; chills 64% |
| metastasis uveal melanoma | Tebentafusp | gp100 × CD3 | phase III  (NCT03070392) | 20 μg d1, 30 μg d8, 68 μg qw thereafter | ORR 9%  mDoR 9.9 mo  mPFS 3.3 mo (95% CI, 3.0-5.0)  mOS 21.7 mo (95% CI, 18.6-28.6)  1-year OS 73% | TRAEs: pyrexia 76%; chills 47%; hypotension 38%  skin-related adverse events: rash 83%; pruritus 69%; and erythema 23% |
| metastatic melanoma | Ipilimumab plus Nivolumab | CTLA-4 plus PD-1 | phase I  (NCT01024231) | concurrent regimen: 0.3-10 mg/kg Nivolumab and 1/3/10 mg/kg Ipilimumab q3w, for 4 doses, followed by Nivolumab q3w, for 4 doses  sequenced regimen: Ipilimumab plus 1/3 mg/kg Nivolumab q2w, for up to 48 doses | ORR 40%, CR 15.6% (in concurrent regimen), 53% (N1I3), 20% (in sequenced regimen) | TRAEs: 93%, rash 55%; pruritus 47%; fatigue 38%; diarrhea 34%  grade ≥ 3 TRAEs: 53%, elevated AST/ALT 15%, elevated levels of lipase 13%, diarrhea/colitis 9%, rash 4% (in concurrent regimen), 18% (in sequenced regimen)  skin toxicity in concurrent regimen: rash 55%, pruritus 47%, vitiligo 11% |
| metastatic melanoma | Ipilimumab plus Nivolumab | CTLA-4 plus PD-1 | phase II  (NCT01927419) | A: 3 mg/kg Ipilimumab plus 1 mg/kg Nivolumab q3w for 4 doses followed by 3 mg/kg Nivolumab  B: 3 mg/kg Ipilimumab plus placebo q3w for 4 doses followed by placebo | BRAF wild-type: ORR 61% (A), 11% (B); CR 22% (A), 0% (B); mPFS 4.4 mo (B)  BRAF mutation–positive: ORR 52% (A), 10% (B); CR 22% (A), 0% (B); mPFS 8.5 mo (A); 2.7 mo (B)  2-years OS: 63.8% (A), 53.6% (B) | A cohort: grade ≥ 3 TRAEs 54%, diarrhea/colitis 28%, elevated AST/ALT 18%, elevated levels of lipase 9%, rash 8%  B cohort: grade ≥ 3 TRAEs 24% |
| metastatic melanoma | Ipilimumab plus Nivolumab | CTLA-4 plus PD-1 | phase III  (NCT01844505) | A: 3 mg/kg Nivolumab q2w  B: 1 mg/kg Nivolumab plus 3 mg/kg Ipilimumab q3w for 4 doses, followed 3 mg/kg Nivolumab q2w for 3 cycle and beyond  C: 3 mg/kg Ipilimumab q3w for 4 doses | ORR: 43.7% (A); 57.6% (B); 19.05 (C)  CR: 8.9% (A); 11.5% (B); 2.2% (C)  mPFS: 6.9 mo (A); 11.5 mo (B, 95%CI, 8.7-19.3); 2.9 mo (C)  mOS: 36.9 mo (A); 19.9 mo (C)  3-years OS: 52% (A); 58% (B); 34% (C)  4-years OS: 53% (B)  5-years OS: 44% (A); 52% (B); 26% (C) | TRAEs in B cohort: 95.5%, diarrhea 44.1%; fatigue 35.1%; pruritus 33.2%  grade ≥ 3 TRAEs: 16.3% (A); 55.0% (B); 16.3% (C) |
| melanoma with previously untreated unresectable stage III/IV | Ipilimumab plus Nivolumab | CTLA-4 plus PD-1 | Phase IIIb/IV  (NCT02714218) | A: 3 mg/kg Nivolumab plus 1 mg/kg Ipilimumab, q3w for 4 doses, followed by Nivolumab 480 mg q4w  B: 1 mg/kg Nivolumab plus 3 mg/kg Ipilimumab, q3w for 4 doses, followed by Nivolumab 480 mg q4w | ORR: 47% (A); 53% (B), stratified HR 0.80 (95%CI, 0.53–1.21)  mPFS: 10.2 mo (A, 95%CI, 6.2-21.9); 10.0 mo (B, 95%CI, 6.3-40.9), stratified HR 1.13 (95%CI, 0.85–1.50)  mOS: NR (A, 95%CI, 43.7-NR); NR (B, 95%CI, 40.8-NR), stratified HR 1.03 (95%CI, 0.75–1.41)  3-years PFS: 38% (A); 43% (B)  3-years OS: 59% (A); 61% (B) | TRAEs in A cohort: diarrhea 27%; fatigue 26%; pruritus 26% (grade ≥ 3: 33.9%)  TRAEs in B cohort: diarrhea 31%; pruritus 29%; rash 27% (grade ≥ 3: 48.3%) |
| melanoma with stage IIIB (70%) or IIIC (30%) | Ipilimumab plus Nivolumab | CTLA-4 plus PD-1 | phase Ib  (NCT02437279) | adjuvant arm: 1 mg/kg Nivolumab plus 3 mg/kg Ipilimumab, 4 courses after surgery  neoadjuvant arm: 1 mg/kg Nivolumab plus 3 mg/kg Ipilimumab, two courses before surgery and two courses postsurgery | neoadjuvant arm: pathological response 78% (n=7/9, pCR 67%) | TRAEs: 100%, elevated ALT 85%; elevated AST 70%; diarrhea 60%; increased GGT 55%; elevated lipase 55%; fatigue 50%; rash 50% |
| melanoma with resected stage IIIB-D or IV | Ipilimumab plus Nivolumab | CTLA-4 plus PD-1 | Phase III  (CheckMate 915) | A: 240 mg Nivolumab q2w 1 mg/kg plus Ipilimumab q6w  B: 480 mg Nivolumab q4w for ≤ 1 year  all after surgery | recurrence events: 35.5% (A); 37.6% (B)  24-mo PFS: 64.6% (A); 63.2% (B)  pts with PD-L1 expression < 1%: recurrence events 45.6% (A), 47.3% (B); mPFS: 33.2 mo (A); 25.3 mo (B)  24-mo OS: 89.8% (A), 91.8% (B) | grade ≥ 3 TRAEs: 32.6% (A); 12.8% (B)  treatment-related deaths: 0.4% (A); 0% (B) |
| metastatic melanoma | BMS-986016 plus Nivolumab | LAG-3 plus PD-1 | phase I/IIa  (NCT01968109) | 80 mg BMS-986016 plus 240 mg Nivolumab q2w | ORR: 11.5%, 18% (LAG-3 ≥ 1%), 5.0% (LAG-3 < 1%), 5.0% (PD-L1 ≥ 1%), 17% (PD-L1< 1%)  DCR: 49%  mDoR NR (range, 0.1-39.3^+^ mo) | TRAEs: 41% (grade ≥ 3: 4.4%) |
| metastatic melanoma who had previously untreated | Relatlimab plus Nivolumab | LAG-3 plus PD-1 | phase II/ III  (NCT03470922) | A: 160 mg Relatlimab plus 480 mg Nivolumab q4w  B: 480 mg Nivolumab q4w | ORR: 43%, CR 16.3% (A); 33%, CR 14.2% (B)  mOS: NR (A, 95%CI, 34.2-NR); 34 mo (B)  12-mo OS: 77.0% (A); 71.6% (B)  24-mo OS: 63.7% (A); 58.3% (B)  mPFS: 10.2 mo (A, 95%CI, 6.5-14.8); 4.6 mo (B); PD-L1≥ 1% 15.7 mo (A, 95% CI, 10.1-25.8), 14.7 mo (B); PD-L1< 1% 6.4 mo (A, 95% CI, 4.6-11.8), 2.9 mo (B) | TRAEs: 81.1%, pruritus 23.4%; fatigue 23.1%; rash 15.5%; arthralgia 14.4%; diarrhea 13.5%; vitiligo 10.4% (grade ≥ 3: 18.9%, A)  discontinuations due to disease progression: 36.3% (A); 46% (B) |

MTD, maximum tolerated dose; RP2D, recommended phase II dose; D1C1, day1 on cycle 1; ORR, overall response rate; CR, complete response; pCR, pathological CR; OS, overall survival; PFS, progression-free survival; DoR, duration of response; NR, not reached; mo, months; TRAEs, treatment-related adverse events; CRS, cytokine release syndrome.

**Table S9** Clinical trials of Nasopharyngeal Cancer

| Tumor | Drug | Target | Phase (ID) | Usage | Outcomes | Adverse Events |
| --- | --- | --- | --- | --- | --- | --- |
| metastatic nasopharyngeal cancer | Cadonilimab (AK104) | PD-1 × CTLA-4 | Phase II  (NCT04220307) | 6 mg/kg, q2w | ORR: 30%, 57.1% (PD-L1 positive); 18.2% (PD-L1 negative)  DCR 70% | TRAEs: anaemia 30.4%; white blood cell count decreased 26.1%; hypothyroidism 26.1%; neutrophil count decreased 21.7%; rash 21.7% (grade ≥ 3: 21.7%) |
| recurrent/metastatic nasopharyngeal cancer | Bintrafusp alfa (M7824) | PD-L1 × TGF-β | phase II  (NCT04396886) | 1200 mg, q2w | ORR 23.7%  1-year OS 57.5%  1-year PFS 23% | grade ≥ 3 TRAEs: 42.4%, anemia 23.7%; secondary malignancies 10.5% |

ORR, overall response rate; DCR, disease control rate; OS, overall survival; PFS, progression-free survival; TRAEs, treatment-related adverse events.

**Table S10** Clinical trials of Renal Cell Carcinoma

| Tumor | Drug | Target | Phase (ID) | Usage | Outcomes | Adverse Events |
| --- | --- | --- | --- | --- | --- | --- |
| metastatic renal cell carcinoma | Ipilimumab plus Nivolumab | CTLA-4 plus PD-1 | phase I  (CheckMate 016) | N3I1: 3 mg/kg Nivolumab plus 1 mg/kg Ipilimumab q3w for 4 doses, followed by 3 mg/kg Nivolumab q2w  N1I3: 1 mg/kg Nivolumab plus 3 mg/kg Ipilimumab q3w for 4 doses, followed by 3 mg/kg Nivolumab q2w | ORR: 40.4%, CR 10.6% (N3I1); 40.4%, PR 40.4% (N1I3)  mPFS: 7.7 mo (N3I1); 9.4 mo (N1I3)  2-year OS: 67.3% (N3I1); 69.6% (N1I3) | grade ≥ 3 TRAEs (N3I1): 38.3%, increased lipase 14.9%; diarrhea 4.3%; pyrexia 4.3%; increased AST/ALT 4.3%; amylase increased 4.3%  grade ≥ 3 TRAEs (N1I3): 61.7%, diarrhea/colitis 29.8%; increased lipase 27.7%; increased AST/ALT 34.1%; amylase increased 6.4% |
| metastatic renal cell carcinoma | Ipilimumab plus Nivolumab | CTLA-4 plus PD-1 | phase III  (CheckMate 214);  (NCT02231749) | A: 3 mg/kg Nivolumab plus 1 mg/kg Ipilimumab q3w for 4 doses, followed by 3 mg/kg Nivolumab q2w  B: 50 mg sunitinib q4w (6-week cycle) | ORR: 42%, CR 9% (A); 39.1%, CR 10.7% (A, ITT); 41.9%, CR 10.4% (A, I/P risk); 27%, CR 1% (B); 32.4% (B, ITT); 26.8% (B, I/P risk);  mPFS: 11.6 mo (A); 8.4 mo (B)  mDoR NR (A, 95%CI, 8.7-15.5)  mPFS 11.6 mo (A, 95%CI, 8.7-15.5)  18-mo OS: 75% (A), 60% (B); 4-year OS: 53.4% (A, ITT), 43.3% (B, ITT), 50% (A, I/P risk), 35.8% (B, I/P risk)  mOS: NR (A, 95%CI, 28.2-NR); NR (A, ITT, 95%CI, 46.7-NR); 48.1 mo (A, I/P risk, 95%CI, 35.6-NR) 26 mo (B) | grade ≥ 3 TRAEs: 46%(A); 63% (B)  TRAEs leading to discontinuation: 22% (A); 12% (B) |

ORR, overall response rate; CR, complete response; PR, partial response; OS, overall survival; PFS, progression-free survival; DoR, duration of response; NR, not reached; mo, months; ITT, intent-to-treat patients; I/P risk, patients with intermediate/poor-risk disease; TRAEs, treatment-related adverse events.

**Table S11** Clinical trials of Malignant Pleural Mesothelioma

| Tumor | Drug | Target | Phase (ID) | Usage | Outcomes | Adverse Events |
| --- | --- | --- | --- | --- | --- | --- |
| recurrent malignant pleural mesothelioma | Ipilimumab plus Nivolumab | CTLA-4 plus PD-1 | phase II  (NCT02716272) | A: 3 mg/kg Nivolumab q2w  B: 3 mg/kg Nivolumab q2w plus 1 mg/kg Ipilimumab q6w | ORR: 19% (A); 28% (B)  12-week DCR: 44% (A); 50% (B)  mDoR: 7.4 mo (A); 8.3 mo (B, 95%CI, 3-14)  mPFS: 4.0 mo (A); 5.6 mo (B, 95%CI, 3.1-8.3)  1-year PFS: 15.9% (A); 22.6% (B)  mOS: 11.9 mo (A); 15.9 mo (B, 95%CI, 10.7-NR)  1-year OS: 49.2% (A); 58.1% (B) | grade ≥ 3 TRAEs: 14% (A), 26% (B), asthenia, asymptomatic increase in aspartate aminotransferase or alanine aminotransferase, asymptomatic lipase increase |
| recurrent malignant pleural mesothelioma | Ipilimumab plus Nivolumab | CTLA-4 plus PD-1 | phase II  (NCT03048474) | 240 mg Nivolumab q2w plus 1 mg/kg Ipilimumab q6w up to 4 times, up to 2 years | 12-week DCR: 67% (PR 29%; SD 38%)  6-mo DCR: 50% (PR 38%; SD 12%)  mDoR: 14.3 mo (95%CI, 6.4-NR)  mPFS: 6.2 mo (95%CI, 4.1-NR)  mOS NR (95%CI, 12.7-NR)  1-year OS: 64% | TRAEs: 94%, infusionrelated reactions 49%; pruritus 31%; fatigue 26%; dry skin 23%; anorexia 20%; diarrhoea 20%; nausea 17%; increased aspartate transaminase 14% (grade ≥ 3: 38%) |
| recurrent malignant pleural mesothelioma | Ipilimumab plus Nivolumab | CTLA-4 plus PD-1 | phase III  (CheckMate 743)  (NCT02899299) | A: 3 mg/kg Nivolumab q2w plus 1 mg/kg Ipilimumab q6w for up to 2 years  B: platinum plus 500 mg/m² pemetrexed chemotherapy plus 75 mg/m² cisplatin or 5 mg/mL carboplatin q3w, for up to six cycles | ORR: 40%, CR 2% (A); 43%, CR 0% (B)  DCR: 77% (A); 85% (B)  mDoR: 11.0 mo (A, 95%CI, 8.1-16.5); 6.7 mo (B)  mPFS: 6.8 mo (A, 95%CI, 5.6-7.4); 7.2 mo (B)  mOS: 18.1 mo (A, 95%CI, 16.8-21.4); 14.1 mo (B)  1-year OS: 68% (A); 58% (B)  2-year OS: 41% (A); 27% (B)  3-year OS: 23% (A); 15% (B) | TRAEs in A cohort: 79%, diarrhoea 17%; pruritus 15%; rash 13%; fatigue 13%; hypothyroidism 11%; nausea 10%  grade ≥ 3 TRAEs: 30% (A); 32% (B) |

ORR, overall response rate; CR, complete response; DCR, disease control rate; OS, overall survival; PFS, progression-free survival; DoR, duration of response; NR, not reached; mo, months; TRAEs, treatment-related adverse events.

**Table S12** Clinical trials of Sarcoma

| Tumor | Drug | Target | Phase (ID) | Usage | Outcomes | Adverse Events |
| --- | --- | --- | --- | --- | --- | --- |
| metastatic sarcoma | Ipilimumab plus Nivolumab | CTLA-4 plus PD-1 | phase II  (NCT02500797) | A: 3 mg/kg Nivolumab q2w  B: 3 mg/kg Nivolumab plus 1 mg/kg Ipilimumab q3w for 4 doses, followed by 3 mg/kg q2w for up to 2 years. | ORR: 5% (A); 16% (B)  mDoR: 6.2 mo (B, 95%CI, 1.4-NR)  6/12-mo CBR: 10%/ 2% (A); 12%/12% (B)  mPFS: 1.7 mo (A); 4.1 mo (B, 95%CI, 1.4-4.7)  mOS: 10.7 mo (A); 14.3 mo (B, 95%CI, 9.6-NR) | TRAEs in cohort B: fatigue 33%, skin rash 19%, adrenal insufficiency 14%; hypothyroidism 14%; pain 14%; anaemia 14% (grade ≥ 3: 14%) |
| metastatic or unresectable angiosarcoma | Ipilimumab plus Nivolumab | CTLA-4 plus PD-1 | phase II  (NCT02834013) | 1 mg/kg Ipilimumab q6w plus 240 mg Nivolumab q2w | ORR: 25%  6-mo PFS: 38%  9-mo OS: 60%  mOS: NR (median follow-up 12.1 mo) | grade ≥ 3 irAEs: 12.5%, AST/ALT increase; diarrhea |

ORR, overall response rate; OS, overall survival; PFS, progression-free survival; DoR, duration of response; NR, not reached; mo, months; TRAEs, treatment-related adverse events; irAEs, immune-related AE.

**Table S13** Clinical trials of LAG-3^+^ Cancer

| Tumor | Drug | Target | Phase (ID) | Usage | Outcomes | Adverse Events |
| --- | --- | --- | --- | --- | --- | --- |
| advanced LAG-3 high expression cancer | Tebotelimab (MGD013) | PD-1 × LAG-3 | Phase I  (NCT03219268) | sequential single-pt cohorts: 1-1200 mg, q2w  tumor-specific expansion cohorts: 600mg, q2w | sequential single-pt cohorts: DCR 58.5% (PR 7.3%, SD 51.2%)  tumor-specific expansion cohorts: DCR 46.7%, CR 0.6%, PR 6.6%; ORR 11%, PR 11% (epithelial ovarian cancer); ORR 6%, PR 6% (triple negative breast cancer); ORR 14%, CR 7% (ICI-naïve NSCLC) | TRAEs: 70.5%, fatigue 19%; nausea 11% (grade ≥ 3: 23.2%) |
| HER2^+^ tumors | Tebotelimab (MGD013) | PD-1 × LAG-3 | phase I  (NCT03219268) | Tebotelimab (300/600 mg) plus Margetuximab 15 mg/kg, q3w | ORR: 19% (CR 1 pts, cholangiocarcinoma; PR 11 pts, 5 breast cancer, 4 colorectal cancer, 2 gastroesophageal junction carcinoma, 1 esophageal adenocarcinoma, 1 endometrial cancer); 33% (without prior anti-HER2 therapy); 14% (with anti-HER2 therapy); 50% (colorectal cancer, n=4/8); 20% (gastroesophageal junction carcinoma, n=2/10); 15% (breast cancer, n=5/33);  mDOR 16.7 mo (95%CI, 11.04-NR)  RP2D 600 mg | TRAEs: 73.8%, diarrhea 15.5%; nausea 14.3%; fatigue 14.3%; AST increased 12%; rash 12%; ALT increased 10.7%; myalgia 10.7% (grade ≥ 3: 16.7%) |
| aHCC | Tebotelimab (MGD013) | PD-1 × LAG-3 | phase I/II  (NCT04212221) | dose escalation: 120/240/400/600 mg, q2w every 28-day cycle  expansion cohort: 600mg, q2w every 28-day cycle | ORR: 8.3%  ICI-naïve patient: ORR 13.3%, DCR 50%  mPFS 2.4 mo  ICI-experienced patient: ORR 3.3%, DCR 46.7%  mPFS 3.1 mo  RP2D 600 mg | grade ≥ 3 TRAEs: 18.8%, hepatic function abnormal 4.3%; amylase increased 2.9%; AST increased 2.9%;  irAEs: 43.5% |
| locally advanced or metastatic GC | Tebotelimab (MGD013) | PD-1 × LAG-3 | phase I  (NCT04178460) | dose escalation: 120/300/600 mg, q2w every 28-day cycle  expansion cohort: 600mg, q2w every 28-day cycle | ORR 5.3%  DCR 52.6%  OS 2.7 mo  PFS 6.5 mo | TEAEs: nausea 63.0%; anemia 59.3%; decreased appetite 51.9%; platelet count decreased 37.0%; hypoalbuminemia 33.3%; vomiting 33.3%; AST increased 29.6%;, constipation 29.6%; weight decreased 29.6% (grade ≥ 3: 66.7%) |

NSCLC, non-small cell lung cancer; aHCC, advanced hepatocellular carcinoma; GC, gastric carcinoma; RP2D, recommended phase II dose; ORR, overall response rate; CR, complete response; PR, partial response; SD, stable disease; DCR, disease control rate; OS, overall survival; PFS, progression-free survival; DoR, duration of response; NR, not reached; mo, months; TRAEs, treatment-related adverse event.
